# Mutant cohesin affects RNA polymerase II regulation in Cornelia de Lange syndrome

**DOI:** 10.1038/srep16803

**Published:** 2015-11-19

**Authors:** Linda Mannini, Fabien C. Lamaze, Francesco Cucco, Clelia Amato, Valentina Quarantotti, Ilaria M Rizzo, Ian D Krantz, Steve Bilodeau, Antonio Musio

**Affiliations:** 1 Istituto di Ricerca Genetica e Biomedica, Consiglio Nazionale delle Ricerche, Pisa, Italy; 2 Centre de recherche sur le cancer de l’Université Laval, Québec, Canada; 3 Centre de recherche du CHU de Québec (Hôtel-Dieu de Québec), Québec, Canada; 4 Division of Human Genetics, The Children’s Hospital of Philadelphia and the Perelman School of Medicine at the University of Pennsylvania, Philadelphia, USA; 5 Département de biologie moléculaire, biochimie médicale et pathologie, Faculté de Médecine, Université Laval, Québec, Canada

## Abstract

In addition to its role in sister chromatid cohesion, genome stability and integrity, the cohesin complex is involved in gene transcription. Mutations in core cohesin subunits *SMC1A, SMC3* and *RAD21,* or their regulators *NIPBL* and *HDAC8,* cause Cornelia de Lange syndrome (CdLS). Recent evidence reveals that gene expression dysregulation could be the underlying mechanism for CdLS. These findings raise intriguing questions regarding the potential role of cohesin-mediated transcriptional control and pathogenesis. Here, we identified numerous dysregulated genes occupied by cohesin by combining the transcriptome of CdLS cell lines carrying mutations in *SMC1A* gene and ChIP-Seq data. Genome-wide analyses show that genes changing in expression are enriched for cohesin-binding. In addition, our results indicate that mutant cohesin impairs both RNA polymerase II (Pol II) transcription initiation at promoters and elongation in the gene body. These findings highlight the pivotal role of cohesin in transcriptional regulation and provide an explanation for the typical gene dysregulation observed in CdLS patients.

Cohesin, a tripartite complex, mediates cohesion of sister chromatids after DNA replication in order to ensure faithful chromosome segregation. Cohesin consists of four subunits, two SMC (Structural Maintenance of Chromosome) proteins, SMC1A and SMC3, and two non-SMC, RAD21 and either SA1 or SA2. Cohesin activity is tightly regulated in association with many additional factors^1^. The loading of cohesin onto chromatin requires the complex NIPBL (a homolog of *Drosophila* Nipped-B)-MAU2^2^,^3^,^4^ and the establishment of bridges between sister chromatids requires ESCO2 (Eco1 in yeast)^5^,^6^,^7^ whereas cohesin removal from chromatin is dependent upon WAPL^8^,^9^. Beyond sister chromatid cohesion, cohesin is also involved in additional biological processes such as DNA damage response, genome surveillance and stability and regulation of gene expression^1^,^10^,^11^,^12^.

Cohesin influences gene expression in a cohesion-independent manner and this activity is sensitive to cohesin dosage. In fact, moderate reduction of cohesin levels affects gene transcription without influencing chromosome segregation^13^. Experimental evidence suggests a role for cohesin in genome organization through long-range interactions with regulatory elements associated with CTCF^14^,^15^,^16^,^17^ or with enhancers and promoters^18^,^19^,^20^,^21^. Indeed, cohesin depletion increases pausing at cohesin-binding genes and decreases gene body transcription, suggesting that cohesin facilitates transition of paused Pol II to elongation^22^.

Mutations in five genes belonging to the cohesin pathway, namely *SMC1A, SMC3, RAD21, NIPBL* and *HDAC8*, have been identified in Cornelia de Lange syndrome (CdLS, OMIM 122470, 300590, 610759, 300882 and 614701) patients^23^,^24^,^25^,^26^,^27^,^28^, a rare developmental disorder characterized by typical facial features, cognitive impairment, growth delay and birth defects including upper extremity anomalies, with a broad variability in phenotypic expression^29^. CdLS cell lines show no evident cohesion defects^30^,^31^ and similar observations have been reported in CdLS model organisms^32^,^33^, arguing that alterations in cohesion are not the etiopathogenetic basis for CdLS. Instead, CdLS cell lines exhibit altered transcriptional profiles, suggesting that CdLS is the result of gene expression dysregulation by a cohesin-dependent mechanism^34^. However, the underlying molecular mechanism is not completely defined. To better understand the effect of cohesin on transcriptional mechanisms, we investigated how the gene transcription machinery was affected by *SMC1A* mutations. First, we identified differentially expressed genes between normal and *SMC1A*-mutated human lymphoblastoid cell lines by using microarrays. Second, we performed genome-wide analyses (chromatin immunoprecipitation coupled with massively parallel DNA sequencing (ChIP-seq) to identify genomic regions occupied by cohesin in normal human lymphoblastoid cells. Next, we compared SMC1A genome-wide occupancy data with gene expression data from CdLS cell lines. Lastly, we investigated the recruitment of Pol II onto twelve differentially expressed genes in CdLS cells carrying *SMC1A* mutations. Our results indicate that cohesin-binding genes are preferentially dysregulated in CdLS and indicate that S*MC1A* mutations affect Pol II and phosphorylated Pol II activity leading to gene expression dysregulation typical of CdLS.

## Results

### Transcriptome analysis reveals that mutations in *SMC1A* lead to gene expression dysregulation

Lymphoblastoid cell lines derived from *SMC1A*-mutated CdLS probands and age-, gender-, and race-compatible healthy controls (Supplementary Table 1) were used to investigate specific gene expression profiles in CdLS. The transcriptome analysis showed that 1,264 probe sets (=1,187 non-redundant genes) were differentially expressed in CdLS (*p* ≤ 0.05, Supplementary Table 2 and Supplementary Table 3). Of the 1,187 dysregulated genes, 571 (48%) were upregulated and 616 (52%) were downregulated. Most of them (79%) showed small fold changes ranging from 1.96 to 1.06 and from −1.07 to −2, respectively, whereas the highest fold changes were 11.66 and −36.36 respectively. Differential expression was validated in a subset of sixteen genes by quantitative RT-PCR experiments (Supplementary Fig. 1).

Gene Spring GX 11.0 software was used to single out any particular functional class (Supplementary Table 4). Automated analysis showed that differentially expressed genes were implicated in metabolic pathways related to glucose and lipid synthesis such as glycolysis, gluconeogenesis, tricarboxylic acid cycle (TCA), cholesterol biosynthesis and acetylcholine synthesis. The NOTCH pathway, which is essential for neuronal and cardiac development^35^, and the EGF-EGFR pathway, which promotes cell proliferation, differentiation, and migration^36^ were affected. In addition, *HOXD1* and *HOXD9*, implicated in morphogenetic and developmental processes of multiple structures including the limbs and nervous system^37^,^38^ and *BMP2* involved in heart development^39^, were also dysregulated in CdLS cell lines. Several genes that encoded factors involved in transcription were also found, including co-activators (*MED24* and *MED25*) and transcription factors (*HES5* and *HES6*) that were downregulated in CdLS when compared to control cell lines. Lastly, components of the Negative Elongation Factor (NELF) complex (*COBRA1* and *WHSC2)* and the *SUPT5H* subunit of the DRB Sensitivity-Inducing Factor (DSIF) were differentially expressed. Altogether, these data suggest that *SMC1A* mutations lead to gene transcription changes in biochemical pathways altered in CdLS.

### Cohesin occupies active genes and correlates with transcription dysregulation in CdLS

**Figure 1:**
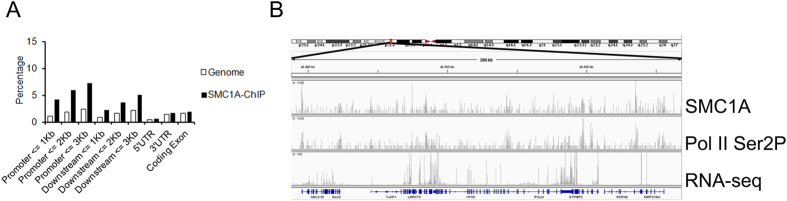
Cohesin distribution in human genome. (**A**) Cohesin binding at active genes in normal human lymphoblastoid cells represented as percentage of sites detected at promoter, downstream, 5′UTR, 3′UTR and coding exons. Cohesin peaks were aligned to RefSeq gene annotations by the use of CEAS tool. We compared binding of cohesin to a selected region to the average genome-wide binding (**B**) Genomic binding of SMC1A cohesin subunit at a selected region of chromosome 6 as determined by ChIP-sequencing. The Pol II Ser 2P binding profile and the RNA-seq data are also shown. This region was chosen to illustrate typical features of cohesin and Pol II binding patterns throughout the human genome.

To gain insight into the mechanism underlying gene transcription dysregulation, we aimed to determine whether genes that change in expression are enriched for cohesin-binding genes. For this, we used ChIP followed by massive parallel DNA sequencing (ChIP-seq) with an antibody against the SMC1A cohesin subunit (in AG14981, CdL060 and CdL074 cell lines) and RNA-seq (in AG14981 and AG09390 cell lines). Genome-wide analysis of the sequenced tags defined 11860 occupied regions for SMC1A (P-value of 10−4 and FDR ≤ 0.05). In mammalian cells, cohesin-binding sites range widely from about 5,000 to more than 100,000 depending on cell type, antibody, and bioinformatic tools^19^,^20^,^40^,^41^. Their association with transcriptional elements was determined using the cis-regulatory element annotation system (CEAS). Data showed that cohesin binds more frequently to promoter and downstream regions than to average genomic regions whereas no enrichment was found within coding exons (Fig. 1A). In addition, cohesin strongly overlaps RNA-seq and Pol II (Fig. 1B). In addition, RNA-seq data revealed that 56% of SMC1A-binding sites were at active transcripts (data not shown), indicating a preferential binding of SMC1A to active genes. To determine the importance of cohesin in gene regulation, we compared SMC1A-occupied genes with genes found to be dysregulated in CdLS cell lines. We found that 979 (11%) of active genes were differentially expressed (397 downregulated and 582 upregulated) and most of them (60%) were occupied by cohesin. On the contrary, of the genes that did not change in expression only 29% were occupied by cohesin (Table 1). These results indicate that cohesin directly controls expression changes of genes associated with CdLS.

### Cohesin controls gene expression levels by reducing Pol II recruitment

**Figure 2:**
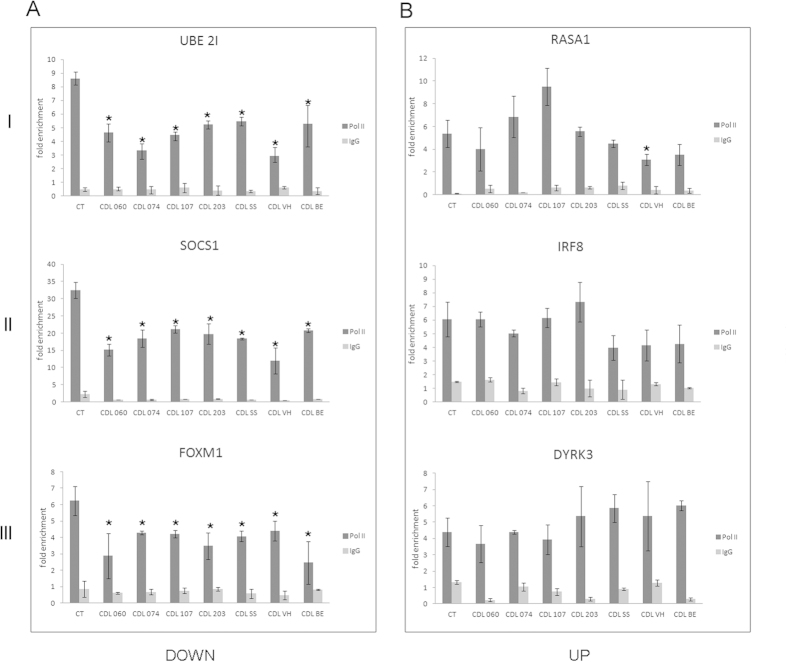
Analysis of Pol II recruitment at the promoter regions by ChIP-qPCR using anti-Pol II antibody (CTD domain). (**A**) Pol II recruitment decreases in CdLS cell lines compared to control cell lines at the promoter regions of downregulated genes in *SMC1A*-mutated CdLS cell lines (**B**) Pol II enrichment does not significantly change in CdLS cell lines at the promoter regions of upregulated genes. I: genes virtually active in all cell types, II: genes preferentially expressed in lymphoblastoid cells, and III: our best targets based on ChIP-Seq enrichment. Since no difference was found, control cell line data were pooled. IgG was used as negative control. Results represent three independent ChIP assays normalized to genomic regions without Pol II enrichment. The average values of the experiments and the relative standard errors are shown. *p < 0.05.

Pol II activity is precisely controlled during transcription. Its recruitment requires the assembly of general transcription factors (GTFs) at the core promoter to form the pre-initiation complex (PIC)^42^,^43^. In order to investigate whether mutant cohesin affects the engagement of Pol II, we analyzed Pol II occupancy at the promoter regions of dysregulated genes. We selected twelve differentially expressed genes, six downregulated and six upregulated, occupied by cohesin. The genes belonged to three specific categories: 1) genes virtually active in all cell types (*UBE2I, RASA1, CHCHD2, RALY*), genes preferentially expressed in lymphoblastoid cells (*SOCS1, IRF8, ILF3, IL2RB*), and 3) our best targets based on ChIP-Seq enrichment (i.e., sites with a high fold enrichment, *FOXM1, DYRK3, DNM2, RAB13*). In addition, we selected two promoter regions of genes whose expression was not affected by *SMC1A* mutations as well as two gene desert regions as further controls. Results showed that Pol II occupancy was significantly reduced (*p* < 0.05) at promoter regions of downregulated genes in CdLS cell lines irrespective of *SMC1A* mutation (Fig. 2A and Supplementary Fig. 2A). On the contrary, no difference was found in Pol II occupancy at upregulated genes as well as in genes whose expression was not affected by *SMC1A* mutations and in gene desert regions (Fig. 2B, Supplementary Fig. 2B and Supplementary Fig. 3A,B). Altogether, these results indicate that mutant cohesin affects transcriptional initiation at downregulated genes.

### *SMC1A* mutations reduces elongating Pol II

**Figure 3:**
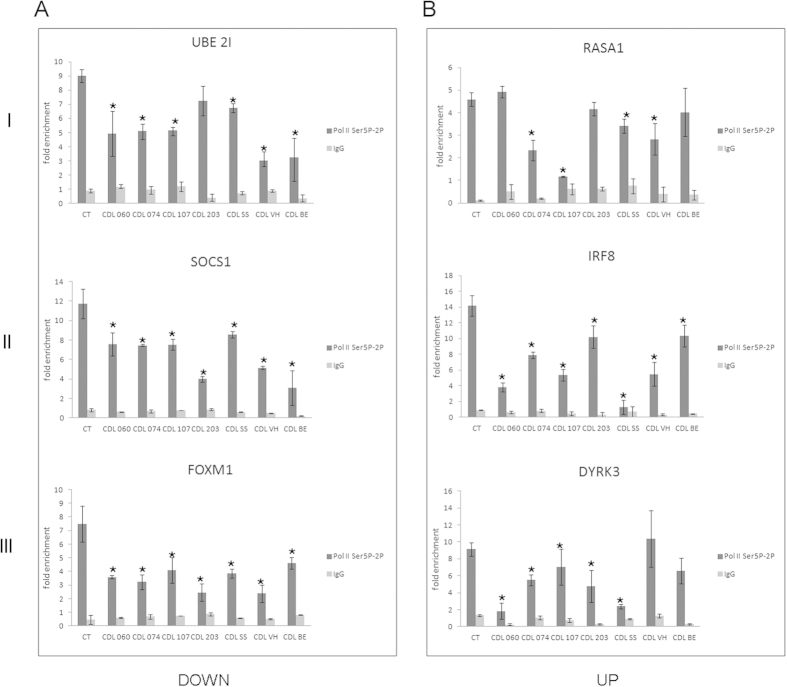
Analysis of the phosphorylated Pol II occupancy using anti-Pol II Ser5P-Ser2P antibody. (**A**) Elongating form of Pol II decreases in the CdLS cell lines compared to control cell lines at the promoter of downregulated genes and (**B**) upregulated genes. I: genes virtually active in all cell types, II: genes preferentially expressed in lymphoblastoid cells, and III: our best targets based on ChIP-Seq enrichment. Since no difference was found, control cell line data were pooled. IgG was used as negative control. Results represent three independent ChIP assays normalized to a genomic region without Pol II enrichment. The average values of the experiments and the relative standard errors are shown. *p < 0.05.

**Figure 4:**
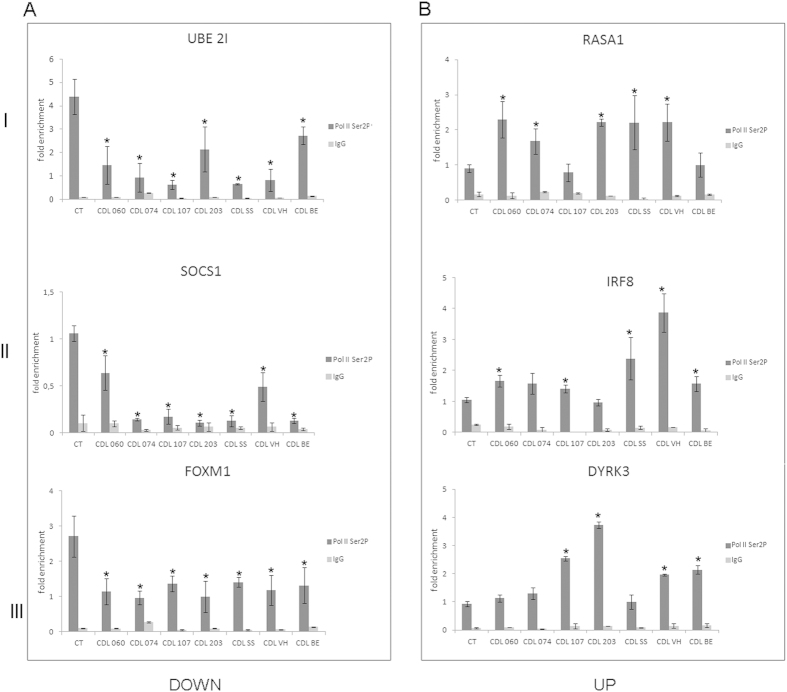
Analysis of the elongating Pol II occupancy at gene body. (**A**) Elongating form of Pol II decreases in CdLS compared to control cell lines at the body gene of downregulated genes whereas (**B**) it decreases at upregulated genes. I: genes virtually active in all cell types, II: genes preferentially expressed in lymphoblastoid cells, and III: our best targets based on ChIP-Seq enrichment. Since no difference was found, control cell line data were pooled. IgG was used as negative control. Results represent three independent ChIP assays normalized to a genomic regions without Pol II enrichment. The average values of the experiments and the relative standard errors are shown. *p < 0.05.

The processivity of Pol II depends on phosphorylation of the C-terminal repeat domain (CTD). In particular, the transition between initiation-pausing and productive elongation is marked by phosphorylation on Ser5 and Ser2 respectively^43^. We asked whether mutant cohesin influences the recruitment of phoshorylated Pol II. Therefore, we analyzed the occupancy of the Pol II by ChIP-qPCR with an antibody recognizing different phosphorylated forms (both Ser5 and Ser2, Supplementary Fig. 4) of Pol II. In addition, we performed ChIP-qPCR experiments using an antibody specific for Ser2P to enrich for the elongating form of Pol II. To validate the specificity of Ser2P antibody, we performed ChIP-seq analysis in AG14981 control lymphoblastoid cells. Results showed that Ser2P antibody worked properly; indeed, genome-wide distribution showed that Ser2P is highly enriched at gene bodies and at downstream regions of genes (around 1000 bp, Supplementary Fig. 5). ChIP-qPCR results showed a reduced level of phosphorylated Pol II at the promoter regions of all classes of downregulated genes (Fig. 3A and Supplementary Fig. 6A). Similar results were obtained for Ser2P (Supplementary Fig. 7A). The analysis performed in the upregulated genes showed that the occupancy of phosphorylated forms of Pol II was also reduced in the majority of CdLS cell lines (Fig. 3B, Supplementary Fig. 6B and Supplementary Fig. 7B). In order to provide a more comprehensive analysis, we also analyzed Pol II occupancy at the gene body regions. Data showed that the occupancy of Pol II Ser2P at the gene body regions decreased at downregulated genes (Fig. 4A) whereas it increased at upregulated genes in most CdLS cell lines (Fig. 4B).

**Figure 5:**
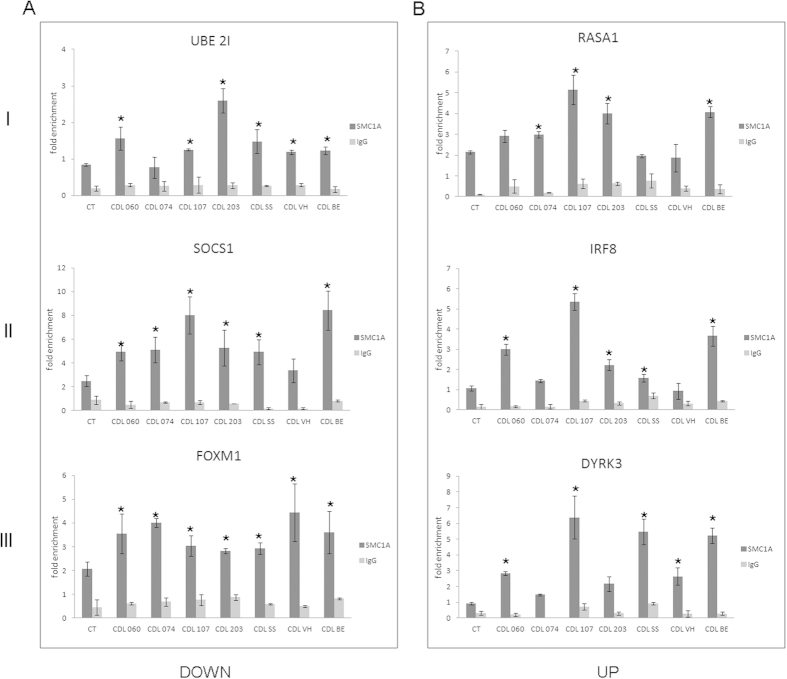
Analysis of SMC1A occupancy at the promoter regions. (**A**) Cohesin binding increases in the CdLS cell lines compared to control cell lines at the promoter of downregulated genes and (**B**) upregulated genes. I: genes virtually active in all cell types, II: genes preferentially expressed in lymphoblastoid cells, and III: our best targets based on ChIP-Seq enrichment. Since no difference was found, control cell line data were pooled. IgG was used as negative control. Results represent three independent ChIP assays and the average values of the experiments and the relative standard errors are shown. *p < 0.05.

Next, we investigated whether *SMC1A* mutations could affect cohesin occupancy to chromatin. We analyzed the occupancy of cohesin at the promoter regions in *SMC1A*-mutated CdLS and control cell lines by ChIP assay. Results showed that cohesin levels increased significantly (*p* < 0.05) at both down- and upregulated genes in most CdLS cell lines (Fig. 5 and Supplementary Fig. 8). Indeed, cohesin presence increased twofold at the promoter of genes virtually active in all cell types *UBE2I, RASA1, RALY* and *CHCHD2*, in genes preferentially expressed in lymphoblastoid cells *SOCS1, ILF3,IRF8* and *IL2RB* by two- to threefold, and in best signal genes *FOXM1, DNM2, DYRK3* and *RAB13* ranging from 1.5- to fourfold (Fig. 5A,B, and Supplementary Fig. 8A,B).

Altogether, these findings support the notion that cohesin controls the expression of cohesin-binding genes and suggest that mutant cohesin impairs the occupancy of Pol II elongating form.

## Discussion

This study provides compelling evidence that mutant cohesin affects Pol II regulation in CdLS. In fact, by combining the transcriptome of CdLS cell lines carrying mutations in *SCM1A* gene with genome-wide data, we found that altered transcriptional initiation and elongation caused transcription dysregulation in genes whose SMC1A binding was increased.

Cohesin is a protein complex evolutionarily conserved from yeast to humans and is involved in a plethora of biological processes, including sister chromatid cohesion, DNA recombination, DNA repair, genome stability surveillance and gene expression regulation^10^,^44^. Cohesin binds the chromatin near the transcription start site of a number of genes and many reports show that cohesin co-localizes with CTCF in mammalian cells, where they play multiple roles in chromatin organization^14^,^15^,^16^,^17^,^18^,^19^,^20^,^21^.

Mutations in cohesin or its regulatory genes are associated with human diseases collectively known as cohesinopathies. CdLS is the most prevalent of the cohesinopathies, with a frequency of about 1:10000. CdLS is caused by mutations in five genes, *NIPBL, SMC1A, SMC3, RAD21* and *HDAC8*, which take into account about 80% of CdLS cases. Many studies have investigated the role of *NIPBL* in both CdLS pathogenesis and transcription regulation^33^,^34^,^45^,^46^. About 70% of CdLS patients have heterozygous mutations in *NIPBL*, the majority being truncating (haploinsufficient), and these types of mutations are often associated with severe phenotypes^29^. Cell lines derived from CdLS patients and mouse models do not show evidence of defects in chromosome cohesion^30^,^31^,^33^. On the other hand, these studies show that *NIPBL* mutations affect gene transcription associated with decreased cohesin loading at differentially expressed genes^34^. Despite ample evidence indicating cooperation between cohesin and NIPBL in gene regulation^18^,^32^,^45^, information regarding the role of core cohesin members in the pathogenesis of CdLS is lacking. Mutations in *SMC1A* have been found in about 5% of CdLS cases and the clinical picture of *SMC1A*-mutated probands is characterized by a mild-to-moderate phenotype^29^. To gain further insight into this issue, we analyzed the transcription profile of CdLS lymphoblastoid cell lines derived from patients harboring mutations in different domains of *SMC1A*. We identified 1,187 dysregulated genes in CdLS. Of these, 616 (52%) were downregulated and 571 (48%) were upregulated. This finding suggests that cohesin can play a dual role in transcription, as activator and repressor, and is consistent with prior observations that cohesin increases expression of some genes and decreases expression of others^13^,^22^,^34^. In *SMC1A*-mutated CdLS cell lines, most of the dysregulated genes (79%) had a fold change ranging from −2 to 1.96. These results indicate that *SMC1A* mutations cause mild alterations in expression of multiple genes, suggesting that collectively, modest cumulative perturbations in gene expression levels contribute to the CdLS phenotype.

Among dysregulated genes identified in *NIPBL* mutant CdLS lines, 59.6% were upregulated and 40.4% downregulated^34^. In addition, among those dyregulated genes^34^, only 115 (with FDR >0.05) genes (9%) were in common with those that we identified in *SMC1A* mutant cells (data not shown). Remarkably, the expression of the *HOXD1, HOXD9* and *BMP2* (see Results section) which are markedly affected in *SMC1A* mutant cells was unchanged in *NIPBL* cell lines. Conversely, the downregulation of *NFATC2* and *PAPSS2*, that could be used as biomarkers for CdLS, which occurs in *NIPBL*-negative cells^34^ was not seen in our *SMC1A* mutant cells. These findings could explain the different phenotypes observed in *NIPBL* and *SMC1A* patients. This notion is supported by the recent observation that *NIPBL* mutations affect gene transcription in two different ways: interfering with the loading of cohesin and its binding to the promoter of active genes independently from the cohesin^45^. Instead, our observations reveal that mutant cohesin influences both Pol II transcription initiation and transition to the elongating form. Therefore, though transcription dysregulation is a hallmark of CdLS cell lines, *NIPBL* and *SMC1A* could have distinct (or partially overlapping) roles in the alteration of gene expression and in CdLS pathogenesis.

The transcriptome profile analysis allowed us to identify specific pathways that are dysregulated in CdLS. Many dysregulated genes were involved in glucose and lipid synthesis, glycolysis and gluconeogenesis, the TCA cycle, cholesterol biosynthesis, adipogenesis and acetylcholine synthesis. It is worth noting that these metabolic pathways have been previously implicated by different approaches^34^,^47^. This finding suggests that the impairment of these coordinated pathways could alter the energy production needed to support fundamental cellular activities. In addition, among the dysregulated genes, we identified several genes that if mutated, produce phenotypes that overlap with CdLS. These include heart development (*BMP2*), limb morphogenesis (*HOXD1* and *HOXD9*), neural proliferation (*HES6, ITCH, HEY1, SRC, LFNG, HES5*) and differentiation (*ROBO1, PDPK1, MAP2K1, MAP4K1, INPPL1, GRB10, RAB5A, RIN1, STAM2, MAP3K3, EIF4E, SOS2, SH3KBP1*)^35^,^36^,^37^,^38^,^39^. Altogether, these specific biochemical pathways provide conceivable causal evidence for some of the phenotypic features seen in CdLS.

The mechanism underlying gene dysregulation observed in CdLS is poorly understood. To address this, we first identified the genomic sites occupied by cohesin at actively transcribed genes in healthy lymphoblastoid cells by genome-wide analyses. We found that cohesin has a greater propensity for localizing at promoter and downstream regions confirming previous results^34^,^48^. In addition, we showed cohesin association with dysregulated genes in CdLS cells, for the first time correlating changes in gene expression with genome distribution of cohesin in a pathological condition.

To understand how mutant cohesin affects gene transcription, we analyzed Pol II recruitment at both the promoter and body regions of selected dysregulated genes in CdLS and control cell lines. Our results showed that Pol II and phosphorylated Pol II occupancy were decreased at the promoter regions of both downregulated and upregulated genes. Instead, the analysis of Ser2P occupancy at gene bodies revealed that the elongating Pol II binding levels continued to be decreased in downregulated genes but increased in upregulated genes. These findings suggest that mutant cohesin impairs Pol II activity at initiation of transcription. This notion is further supported by the observation that in *Drosophila,* cohesin depletion affects Pol II occupancy and the transition to elongation^22^. It is reasonable to deduce that mutant cohesin and cohesin depletion act by different mechanisms to regulate gene expression, though the end result is similar. Many studies indicate that cohesin facilitates enhancer-promoter communication, and subsequently the transition of promoter-proximal paused Pol II to elongation^12^,^22^,^49^. To date, no mutations leading to SMC1A null protein have been identified, arguing that such mutations are incompatible with life. All the identified *SMC1A* mutations are missense or small in-frame deletions^29^ and they do not affect mRNA stability, protein levels or formation of the cohesin complex^31^,^47^. However, mutated proteins show an increased affinity for chromatin compared to wild-type, making the cohesin-DNA binding more stable^31^. Consistent with this view, we found that cohesin occupancy increased significantly at the promoter regions of dysregulated genes in CdLS cell lines. We speculate that mutant cohesin in CdLS cell lines leads to a steric/physical obstruction that limits the recruitment of Pol II and in turn causes a decrease in Pol II occupancy and gene downregulation. As a consequence, mutant cohesin influenced Pol II elongation activity in down- and upregulated genes, suggesting that cohesin causes global disturbance in transcription when mutated. This notion is further supported by the finding that mutations in *AFF4* gene are associated with CHOPS syndrome which phenotypically overlaps CdLS^48^. AFF4 is a component of the superelongation complex^50^, and mutations result in transcriptional elongation abnormalities leading to gene transcription dysregulation. Furthermore, genome-wide data showed that altered distribution of AFF4 binding sites affects cohesin binding in CHOPS syndrome^48^, providing a common pathogenetic mechanism in both CHOPS and CdLS. The role of cohesin in gene upregulation is less clear. Our data indicates that mutant cohesin had no effect on Pol II recruitment at the promoter regions, while it impaired the occupancy of the elongating form of Pol II at upregulated genes. We suppose that gene upregulation is caused by altered expression of factors that act broadly on many or all genes, such as basal transcription factors. Further work will address how cohesin interacts with Pol II to modulate gene expression.

In summary, we have found that cohesin-binding genes are enriched in dysregulated genes identified in CdLS. In addition, our data indicate that mutant cohesin affects the occupancy of both Pol II and the transition to the Pol II elongating form, providing a molecular mechanism for the typical altered transcription profiles observed in CdLS.

## Methods

### Cell culture

Lymphoblastoid cell lines (LCLs) were grown in RPMI 1640 medium supplemented with 10% fetal bovine serum, 100 U/ml penicillin, 0.1 mg/ml streptomycin, and 1% L-glutamine. We used CdLS lymphoblastoid cell lines derived from patients carrying mutations in the *SMC1A* gene previously described by our laboratories^26^,^47^,^51^ (Supplementary Table 1). This study was conducted according to the principles expressed in the Declaration of Helsinki. All patients were enrolled under an IRB-approved protocol of informed consent at The Children’s Hospital of Philadelphia. In addition, we used four cell lines purchased from Coriell Cell Repositories as control samples (Supplementary Table 1).

### Library preparation and ChIP sequencing

DNA recovered from the ChIP procedure was quantified using the Qubit 2.0 Fluorometer (Invitrogen) and the quality was tested by the Agilent 2100 Bioanalyzer (Agilent Technologies). The DNA was then processed, including end repair, adaptor ligation, and size selection, using an Ovation® Ultralow System V2 1–16 (Nugen) sample prep kit following the manufacturer’s instructions. Final DNA libraries were validated and processed with Illumina cBot for cluster generation on the flowcell, following the manufacturer’s instructions and sequenced on single-end 50 bp mode at the on HiSeq2500 (Illumina) at a depth of approximately 30–50 million sequences per sample. The CASAVA 1.8.2 version of the Illumina pipeline was used to process raw data for both format conversion and de-multiplexing.

### ChIP-seq analysis

Raw sequence files were subjected to quality control analysis using FastQC ( http://www.bioinformatics.babraham.ac.uk/projects/fastqc/). In order to avoid low-quality data, adapters were removed by Cutadapt^52^ and lower quality bases were trimmed by ERNE^53^ so that the quality criteria of quality value per base (Phred score) was at least 35 and the read length was at least 30 bp. The quality-checked reads were mapped to the human reference genome GRCh37/hg19 using Bowtie 2.0.2^54^. Only uniquely mapping reads were used for the peak calling by MACS2^55^ with 0.05 FDR used as a cut-off value, and with standard parameters for shifting model calculations. MACS2 was also used for all comparisons between input track as control and each one of the data sets as treatment. Binding site overlaps between different sample sets were obtained using custom UNIX shell scripting. Genome-wide analysis of enrichment of chromosomal features and chromosomal distribution of ChIP regions were determined using the CEAS package^56^.

### Library preparation and RNA sequencing

‘TruSeq Stranded mRNA Sample Prep kit’ (Illumina) was used for library preparation following the manufacturer’s instructions, starting with 1–2 μg of good quality RNA (R.I.N. >7) as input. The poly-A mRNA was fragmented for 3 min at 94 °C and every purification step was performed using 1X Agencourt AMPure XP beads.

Both RNA samples and final libraries were quantified by using the Qubit 2.0 Fluorometer (Invitrogen) and quality tested by Agilent 2100 Bioanalyzer RNA Nano assay (Agilent technologies). Libraries were then processed with Illumina cBot for cluster generation on the flowcell, following the manufacturer’s instructions and sequenced on single-end mode on HiSeq2500 (Illumina). The CASAVA 1.8.2 version of the Illumina pipeline was used to process raw data for both format conversion and de-multiplexing.

### RNA-Seq analysis

Raw sequence files were subjected to quality control analysis using FastQC ( http://www.bioinformatics.babraham.ac.uk/projects/fastqc/). In order to avoid low-quality data, adapters were removed by Cutadapt and lower quality bases were trimmed by ERNE. For the analysis of differentially expressed genes, the quality-checked reads were processed using the TopHat version 2.0.0 package (Bowtie 2 version 2.2.0) as FASTQ files. Reads were mapped to the human reference genome GRCh37/hg19. Read abundance was evaluated and normalized by using Cufflinks for each gene and Cuffdiff from the Cufflinks 2.2.0 package was used to calculate the differential expression levels and evaluate the statistical significance of detected alterations. Only protein-coding genes were considered and gene level expression values were determined by read per kilobase of exon per million fragments mapped (RPKM). All genes with RPKM >1 were designated as expressed.

Genome mapping of SMC1A and Pol II binding regions, and RNA-seq data were visualized using the Integrative Genomics Viewer interface^57^.

### Microarray expression analysis

Total RNA was extracted from both CdLS cell lines as well as healthy control cells using the RNAeasy Mini-kit (Qiagen). Synthesis of cyanine-labeled cRNA was performed using Quick Amplification Labeling Two Color kit (Agilent) and hybridizations of the labeled cRNA were performed using oligo-microarrays containing about 28,000 probe sets covering the whole human genome by Gene Expression Hybridization (Agilent). Images were quantified with Feature Extraction 10.5 (Agilent) and microarray data were normalized using locally weighted scatterplot smoothing (LOWESS) algorithm. Microarray data were screened according to expression level, fold change and standard deviation by GeneSpring 11.0 software. Student’s *t*-test was used to test for statistical significance in gene expression between CdLS cell lines and healthy control cells. A False Discovery Rate (Benjamini-Hochberg) was used to correct *p*-value. Data with a corrected *p*-value of <0.05 and fold change >1 were considered statistically significant.

### Pathway analysis/function and pathway enrichment analysis

The differentially expressed genes were functionally analyzed for biological processes using Gene Spring GX 11.0 software supported by BioPAX. For each term, the *p*-value was calculated and a term with *p* < 0.05 was considered to be enriched.

### cDNA synthesis and quantitative real-time PCR (qRT-PCR)

Total RNA was extracted from CdLS and healthy control cell lines using RNAeasy Mini-kit (Qiagen) and cDNA was synthesized with SuperScript^TM^ II reverse transcriptase using oligo dT (Invitrogen). Reactions were assembled using QuantiTecT SYBER Green PCR mix (Qiagen) and PCR analyses were carried out using Rotor Gene 3000 (Corbett). qPCR reactions were run in duplicate and normalized with respect to GAPDH. Since no difference was found in control cell lines, data was pooled. Primers used for mRNA expression analysis are reported in Supplementary Table 5. Student’s *t*-test was used to test for statistical significance in gene expression between CdLS cell lines and healthy control cells.

### ChIP-qPCR in *SMC1A*-mutated CdLS cell lines

ChIP assays were performed as previously described^47^ with minor modifications. Briefly, 10^7^–10^8^ cells were crosslinked with 1% formaldehyde for 15 min and quenched with 125 mM glycine. Pellets of cells were incubated with lysis buffer 1 (50 mM HEPES-KOH, pH 7.5, 140 mM NaCl, 1 mM EDTA, 10% glycerol, 0.5% NP-40 and 0.25% Triton X-100), then lysis buffer 2 (10 mM Tris-HCl, 100 mM NaCl and 1 mM EDTA) and sonicated in lysis buffer 3 (10 mM Tris-HCl, 100 mM NaCl, 1mM EDTA, 0.1% sodium deoxycolate and 0.5 sarkosyl). Each sample was incubated with Dynabeads protein A or G (Invitrogen) previously bound with 10 μg of specific antibody. The antibodies used were as follows: SMC1A (Bethyl Laboratories, A300-055A), Pol II that recognizes the C-terminal heptapeptide repeat region on the largest subunit of Pol II (Covance 8WG16), Ser2P-Ser5Pol II (Abcam 4H8, ab5408), Ser2P Pol II (Abcam H5, ab24758, and an antibody kindly gifted by Prof. Katsuhiko Shirahige) used with IgG-IgM linker antibody (Millipore), negative control mouse IgG (Sigma) and rabbit IgG (Sigma). Next, the beads were washed with low salt buffer (20 mM Tris-HCl, pH 8, 150 mM NaCl, 0.5 mM EDTA, 0.1% SDS, 1% Triton X-100), high salt buffer (20 mM Tris-HCl, pH 8, 500 mM NaCl, 0.5 mM EDTA, 0.1% SDS, 1% Triton X-100) and RIPA buffer (50 mM HEPES-KOH, pH 7.5, 0.5 mM EDTA, 10% NP-40, 0.7% sodium deoxycolate and 0.5 M LiCl) and eluted overnight at 65 °C. The eluates were incubated with proteinase K and the purificate with QIAquick Purification Kit (Qiagen). For each cell line, at least three independent ChIP assays were carried out and analyzed by qPCR. Each sample was run in duplicate and repeated at least three times. Corresponding primers of the selected genes are described in Supplementary Table 6. Enrichment was determined relative to a genomic region (chr1:153,998,597-153,998,804) that does not bind Pol II. Results were analyzed by Student’s *t*-test. *P*-values of <0.05 were considered statistically significant.

## Additional Information

**Accession codes:** Next generation sequencing data are available on GEO (GSE38395) and NCBI (SRP057545) DataSets.

**How to cite this article**: Mannini, L. *et al.* Mutant cohesin affects RNA polymerase II regulation in Cornelia de Lange syndrome. *Sci. Rep.* **5**, 16803; doi: 10.1038/srep16803 (2015).

## Supplementary Material

Supplementary Information

## Figures and Tables

**Table 1:** Cohesin-binding at active genes in CdLS.

Active genes						
Differentially expressed				Not changing		
	11%				89%	
SMC1A binding		No SMC1A binding		SMC1A binding		No SMC1A binding
60%		40%		29%		71%
