# Supplementary material for: Mutant cohesin affects RNA polymerase II regulation in Cornelia de Lange syndrome
Source: Sci Rep. 2015 Nov 19;5:16803. doi: 10.1038/srep16803 (PMC4652179; doi:10.1038/srep16803)

Supplementary information

Mutant cohesin affects RNA polymerase II regulation in Cornelia de Lange syndrome

Linda Mannini, Fabien Lamaze, Francesco Cucco, Clelia Amato, Valentina Quarantotti, Ilaria M Rizzo, Ian D Krantz, Steve Bilodeau, Antonio Musio

Supplementary Table 1. CdLS and control cell lines used for transcriptome analysis.

**CdLS cell line**	Gender	Mutation	Amino acid change	Protein domain
CdL107	Female	c.173_187del15nt	Val58_Arg62del	Amino terminal P-loop NTPase domain
CdLVH	Male	c.1486C>T	Arg496Cys	hinge-coiled-coil transition
CdLSS	Female	c.1486C>T	Arg496Cys	hinge-coiled-coil transition
CdL BE	Female	c.1487G>A	Arg496His	hinge-coiled-coil transition
CdL060	Female	c.1487G>A	Arg496His	hinge-coiled-coil transition
CdL203	Female	c.1487G>A	Arg496His	hinge-coiled-coil transition
CdL074	Female	c.3364T>C	Phe1122Leu	Carboxy terminal P-loop NTPase domain
**Control cell line**				
AG09390	Female			
AG09387	Male			
AG14981	Female			
AG09393	Female			

Supplementary Table 2. Differentially expressed genes in *SMC1A* mutated cell lines (P<0.05).

	**Total genes**	**Up-regulated genes**	**Down-regulated genes**
**Total probes**	1264	611	653
**Non redundant genes**	1187	571	616

Supplementary Table 3. Dysregulated genes in *SMC1A* mutated cells by microarray analysis (*P*<0.05).

GeneSymbol	Regulation	FC	Description
LEPREL1	up	11,66907	Homo sapiens leprecan-like 1 (LEPREL1), transcript variant 1, mRNA [NM_018192]
PFN2	up	8,884887	Homo sapiens profilin 2 (PFN2), transcript variant 1, mRNA [NM_053024]
ANXA1	up	8,555794	Homo sapiens annexin A1 (ANXA1), mRNA [NM_000700]
PRKCH	up	8,461679	Homo sapiens protein kinase C, eta (PRKCH), mRNA [NM_006255]
CD28	up	8,049169	Homo sapiens CD28 molecule (CD28), mRNA [NM_006139]
ZNF880	up	7,076735	Homo sapiens zinc finger protein 880 (ZNF880), mRNA [NM_001145434]
FEZ1	up	6,915433	Homo sapiens fasciculation and elongation protein zeta 1 (zygin I) (FEZ1), transcript variant 1, mRNA [NM_005103]
ZNF608	up	6,776035	Homo sapiens zinc finger protein 608 (ZNF608), mRNA [NM_020747]
GPAT2	up	6,349985	Homo sapiens glycerol-3-phosphate acyltransferase 2, mitochondrial (GPAT2), nuclear gene encoding mitochondrial protein, mRNA [NM_207328]
RND3	up	6,145313	Homo sapiens Rho family GTPase 3 (RND3), mRNA [NM_005168]
PTPN13	up	6,030349	Homo sapiens protein tyrosine phosphatase, non-receptor type 13 (APO-1/CD95 (Fas)-associated phosphatase) (PTPN13), transcript variant 4, mRNA [NM_080685]
PLOD2	up	5,691338	Homo sapiens procollagen-lysine, 2-oxoglutarate 5-dioxygenase 2 (PLOD2), transcript variant 1, mRNA [NM_182943]
ST3GAL6	up	5,599227	Homo sapiens ST3 beta-galactoside alpha-2,3-sialyltransferase 6 (ST3GAL6), mRNA [NM_006100]
TBC1D4	up	5,181232	Homo sapiens TBC1 domain family, member 4 (TBC1D4), mRNA [NM_014832]
IL2RA	up	5,015806	Homo sapiens interleukin 2 receptor, alpha (IL2RA), mRNA [NM_000417]
GPAT2	up	4,996553	Homo sapiens glycerol-3-phosphate acyltransferase 2, mitochondrial (GPAT2), nuclear gene encoding mitochondrial protein, mRNA [NM_207328]
PLAG1	up	4,768599	Homo sapiens pleiomorphic adenoma gene 1 (PLAG1), transcript variant 1, mRNA [NM_002655]
NRIP1	up	4,728106	Homo sapiens nuclear receptor interacting protein 1 (NRIP1), mRNA [NM_003489]
KLHL29	up	4,417487	Homo sapiens kelch-like 29 (Drosophila) (KLHL29), mRNA [NM_052920]
TUBB2A	up	4,270917	Homo sapiens tubulin, beta 2A (TUBB2A), mRNA [NM_001069]
TMEM144	up	4,151937	Homo sapiens transmembrane protein 144 (TMEM144), mRNA [NM_018342]
ITGB1BP1	up	4,111365	Homo sapiens integrin beta 1 binding protein 1 (ITGB1BP1), transcript variant 1, mRNA [NM_004763]

GPAT2	up	4,021498	Homo sapiens glycerol-3-phosphate acyltransferase 2, mitochondrial (GPAT2), nuclear gene encoding mitochondrial protein, mRNA [NM_207328]
MNDA	up	3,879569	Homo sapiens myeloid cell nuclear differentiation antigen (MNDA), mRNA [NM_002432]
TRPM2	up	3,757673	Homo sapiens transient receptor potential cation channel, subfamily M, member 2 (TRPM2), transcript variant 1, mRNA [NM_003307]
SLC12A8	up	3,630023	Homo sapiens solute carrier family 12 (potassium/chloride transporters), member 8 (SLC12A8), transcript variant 1, mRNA [NM_024628]
ROBO1	up	3,556089	Homo sapiens roundabout, axon guidance receptor, homolog 1 (Drosophila) (ROBO1), transcript variant 2, mRNA [NM_133631]
UNC13B	up	3,332733	Homo sapiens unc-13 homolog B (C, elegans) (UNC13B), mRNA [NM_006377]
MAGI3	up	3,311869	Homo sapiens membrane associated guanylate kinase, WW and PDZ domain containing 3 (MAGI3), transcript variant 2, mRNA [NM_152900]
ZNF880	up	3,266943	Homo sapiens zinc finger protein 880 (ZNF880), mRNA [NM_001145434]
CLIC4	up	3,247167	Homo sapiens chloride intracellular channel 4 (CLIC4), nuclear gene encoding mitochondrial protein, mRNA [NM_013943]
LRRC49	up	3,205096	Homo sapiens leucine rich repeat containing 49 (LRRC49), transcript variant 1, mRNA [NM_001199017]
HRASLS2	up	3,173319	Homo sapiens HRAS-like suppressor 2 (HRASLS2), mRNA [NM_017878]
LRP2	up	3,161781	Homo sapiens low density lipoprotein receptor-related protein 2 (LRP2), mRNA [NM_004525]
GABARAPL1	up	3,087127	Homo sapiens GABA(A) receptor-associated protein like 1 (GABARAPL1), mRNA [NM_031412]
TTLL7	up	3,078939	Homo sapiens tubulin tyrosine ligase-like family, member 7 (TTLL7), mRNA [NM_024686]
PRKAR1B	up	3,018139	Homo sapiens protein kinase, cAMP-dependent, regulatory, type I, beta (PRKAR1B), transcript variant 1, mRNA [NM_001164761]
PLEKHM1	up	3,011212	Homo sapiens pleckstrin homology domain containing, family M (with RUN domain) member 1 (PLEKHM1), transcript variant 1, mRNA [NM_014798]
TMEM45A	up	3,009332	Homo sapiens transmembrane protein 45A (TMEM45A), mRNA [NM_018004]
CD109	up	2,934279	Homo sapiens CD109 molecule (CD109), transcript variant 1, mRNA [NM_133493]
UGT2B10	up	2,914247	Homo sapiens UDP glucuronosyltransferase 2 family, polypeptide B10 (UGT2B10), transcript variant 1, mRNA [NM_001075]
LOC644450	up	2,908759	Homo sapiens hypothetical protein LOC644450, mRNA (cDNA clone IMAGE:4606942), partial cds, [BC022881]
TNS3	up	2,850104	Homo sapiens tensin 3 (TNS3), mRNA [NM_022748]
RABEP1	up	2,837492	Homo sapiens rabaptin, RAB GTPase binding effector protein 1 (RABEP1), transcript variant 1, mRNA [NM_004703]

CLIC4	up	2,795716	Homo sapiens chloride intracellular channel 4 (CLIC4), nuclear gene encoding mitochondrial protein, mRNA [NM_013943]
TGFBR3	up	2,795238	Homo sapiens transforming growth factor, beta receptor III (TGFBR3), transcript variant 1, mRNA [NM_003243]
GPNMB	up	2,79377	Homo sapiens glycoprotein (transmembrane) nmb (GPNMB), transcript variant 1, mRNA [NM_001005340]
HSBP1L1	up	2,746187	Homo sapiens heat shock factor binding protein 1-like 1 (HSBP1L1), mRNA [NM_001136180]
UBD	up	2,709664	Homo sapiens ubiquitin D (UBD), mRNA [NM_006398]
C11orf41	up	2,707999	Homo sapiens chromosome 11 open reading frame 41 (C11orf41), mRNA [NM_012194]
LOC100127983	up	2,654672	Homo sapiens hypothetical protein LOC100127983 (LOC100127983), mRNA [NM_001190972]
CLIC4	up	2,607526	Homo sapiens chloride intracellular channel 4 (CLIC4), nuclear gene encoding mitochondrial protein, mRNA [NM_013943]
PHC1	up	2,606995	Homo sapiens polyhomeotic homolog 1 (Drosophila) (PHC1), mRNA [NM_004426]
ZNF608	up	2,598061	Homo sapiens zinc finger protein 608 (ZNF608), mRNA [NM_020747]
C11orf74	up	2,54176	Homo sapiens chromosome 11 open reading frame 74 (C11orf74), mRNA [NM_138787]
CLMN	up	2,509799	Homo sapiens calmin (calponin-like, transmembrane) (CLMN), mRNA [NM_024734]
ZSWIM7	up	2,502497	Homo sapiens zinc finger, SWIM-type containing 7 (ZSWIM7), transcript variant 1, mRNA [NM_001042697]
PTPN22	up	2,463335	Homo sapiens protein tyrosine phosphatase, non-receptor type 22 (lymphoid) (PTPN22), transcript variant 1, mRNA [NM_015967]
PPFIBP1	up	2,458832	Homo sapiens PTPRF interacting protein, binding protein 1 (liprin beta 1) (PPFIBP1), transcript variant 1, mRNA [NM_003622]
CTSZ	up	2,454215	Homo sapiens cathepsin Z, mRNA (cDNA clone IMAGE:3929674), containing frame-shift errors, [BC025419]
NMT2	up	2,447186	Homo sapiens N-myristoyltransferase 2 (NMT2), mRNA [NM_004808]
HEBP2	up	2,428851	Homo sapiens heme binding protein 2 (HEBP2), mRNA [NM_014320]
HELB	up	2,381742	Homo sapiens helicase (DNA) B (HELB), mRNA [NM_033647]
ARHGEF7	up	2,378133	Homo sapiens Rho guanine nucleotide exchange factor (GEF) 7 (ARHGEF7), transcript variant 2, mRNA [NM_145735]
PLG	up	2,377524	Homo sapiens plasminogen (PLG), transcript variant 1, mRNA [NM_000301]
CACNA1A	up	2,375855	Homo sapiens calcium channel, voltage-dependent, P/Q type, alpha 1A subunit (CACNA1A), transcript variant 2, mRNA [NM_023035]
PTPN22	up	2,359427	Homo sapiens protein tyrosine phosphatase, non-receptor type 22 (lymphoid) (PTPN22), transcript variant 2, mRNA [NM_012411]

HSPA6	up	2,303011	Homo sapiens heat shock 70kDa protein 6 (HSP70B') (HSPA6), mRNA [NM_002155]
KIAA1407	up	2,299393	Homo sapiens KIAA1407 (KIAA1407), mRNA [NM_020817]
SOCS2	up	2,275017	Homo sapiens suppressor of cytokine signaling 2 (SOCS2), mRNA [NM_003877]
RPS14	up	2,261003	ribosomal protein S14 [Source:HGNC Symbol;Acc:10387] [ENST00000401695]
GAA	up	2,206779	Homo sapiens glucosidase, alpha; acid (GAA), transcript variant 1, mRNA [NM_000152]
C13orf15	up	2,182494	Homo sapiens chromosome 13 open reading frame 15 (C13orf15), mRNA [NM_014059]
FOXO3	up	2,168531	Homo sapiens forkhead box O3 (FOXO3), transcript variant 1, mRNA [NM_001455]
CRNDE	up	2,162417	Homo sapiens colorectal neoplasia differentially expressed (non-protein coding) (CRNDE), transcript variant 1, non-coding RNA [NR_034105]
MLL3	up	2,156976	Homo sapiens myeloid/lymphoid or mixed-lineage leukemia 3 (MLL3), mRNA [NM_170606]
ATXN1	up	2,138465	Homo sapiens ataxin 1 (ATXN1), transcript variant 1, mRNA [NM_000332]
HIPK2	up	2,137493	Homo sapiens homeodomain interacting protein kinase 2 (HIPK2), transcript variant 1, mRNA [NM_022740]
CPEB3	up	2,121845	Homo sapiens cytoplasmic polyadenylation element binding protein 3 (CPEB3), transcript variant 1, mRNA [NM_014912]
SH2B2	up	2,114922	Homo sapiens SH2B adaptor protein 2 (SH2B2), mRNA [NM_020979]
PRKAR1B	up	2,10782	Homo sapiens protein kinase, cAMP-dependent, regulatory, type I, beta (PRKAR1B), transcript variant 2, mRNA [NM_002735]
SLC2A14	up	2,106705	solute carrier family 2 (facilitated glucose transporter), member 14 [Source:HGNC Symbol;Acc:18301] [ENST00000431042]
RFESD	up	2,102777	Homo sapiens Rieske (Fe-S) domain containing (RFESD), transcript variant 2, mRNA [NM_173362]
KLHL13	up	2,096597	Homo sapiens kelch-like 13 (Drosophila) (KLHL13), transcript variant 1, mRNA [NM_033495]
NMT2	up	2,058769	Homo sapiens N-myristoyltransferase 2 (NMT2), mRNA [NM_004808]
DYRK3	up	2,038917	Homo sapiens dual-specificity tyrosine-(Y)-phosphorylation regulated kinase 3 (DYRK3), transcript variant 2, mRNA [NM_001004023]
FCRLA	up	2,02729	Homo sapiens Fc receptor-like A (FCRLA), transcript variant 2, mRNA [NM_032738]
KIAA0114	up	2,013699	Homo sapiens KIAA0114 (KIAA0114), non-coding RNA [NR_024031]
LOC100128292	up	2,013008	Homo sapiens hypothetical LOC100128292 (LOC100128292), non-coding RNA [NR_024585]
ECI2	up	2,010106	Homo sapiens enoyl-CoA delta isomerase 2 (ECI2), transcript variant 2, mRNA [NM_206836]
NRIP1	up	2,001576	Homo sapiens nuclear receptor interacting protein 1 (NRIP1), mRNA [NM_003489]
MGST1	up	1,963258	Homo sapiens microsomal glutathione S-transferase 1 (MGST1), transcript variant 1c, mRNA [NM_145791]
DGCR11	up	1,956816	Homo sapiens DiGeorge syndrome critical region gene 11 (DGCR11), non-coding RNA [NR_024157]

ERP44	up	1,941162	Homo sapiens endoplasmic reticulum protein 44 (ERP44), mRNA [NM_015051]
RAB13	up	1,927346	Homo sapiens RAB13, member RAS oncogene family (RAB13), mRNA [NM_002870]
REP15	up	1,927079	Homo sapiens RAB15 effector protein (REP15), mRNA [NM_001029874]
AMPD3	up	1,915038	Homo sapiens adenosine monophosphate deaminase 3 (AMPD3), transcript variant 3, mRNA [NM_001025390]
RAB11FIP1	up	1,91493	Homo sapiens RAB11 family interacting protein 1 (class I) (RAB11FIP1), transcript variant 3, mRNA [NM_001002814]
TPRG1	up	1,88454	Homo sapiens tumor protein p63 regulated 1 (TPRG1), mRNA [NM_198485]
PIGN	up	1,881616	Homo sapiens phosphatidylinositol glycan anchor biosynthesis, class N (PIGN), transcript variant 1, mRNA [NM_176787]
SEPT8	up	1,873391	Homo sapiens septin 8 (SEPT8), transcript variant 1, mRNA [NM_001098811]
NOL4	up	1,87022	Homo sapiens nucleolar protein 4 (NOL4), transcript variant 1, mRNA [NM_003787]
VAPA	up	1,859997	Homo sapiens VAMP (vesicle-associated membrane protein)-associated protein A, 33kDa (VAPA), transcript variant 1, mRNA [NM_003574]
FCRLA	up	1,85691	Homo sapiens Fc receptor-like A (FCRLA), transcript variant 2, mRNA [NM_032738]
IRF8	up	1,854219	Homo sapiens interferon regulatory factor 8 (IRF8), mRNA [NM_002163]
GDPD5	up	1,851982	Homo sapiens glycerophosphodiester phosphodiesterase domain containing 5 (GDPD5), mRNA [NM_030792]
UNC13B	up	1,850999	Homo sapiens unc-13 homolog B (C, elegans) (UNC13B), mRNA [NM_006377]
TWSG1	up	1,840081	Homo sapiens twisted gastrulation homolog 1 (Drosophila) (TWSG1), mRNA [NM_020648]
TMEM37	up	1,834361	transmembrane protein 37 [Source:HGNC Symbol;Acc:18216] [ENST00000409826]
C6orf192	up	1,828863	Homo sapiens chromosome 6 open reading frame 192 (C6orf192), mRNA [NM_052831]
BTK	up	1,817022	Homo sapiens Bruton agammaglobulinemia tyrosine kinase (BTK), mRNA [NM_000061]
PIP5K1B	up	1,802444	Homo sapiens phosphatidylinositol-4-phosphate 5-kinase, type I, beta (PIP5K1B), transcript variant 2, mRNA [NM_003558]
PPA1	up	1,799704	Homo sapiens pyrophosphatase (inorganic) 1 (PPA1), mRNA [NM_021129]
RASA1	up	1,789689	Homo sapiens RAS p21 protein activator (GTPase activating protein) 1 (RASA1), transcript variant 1, mRNA [NM_002890]
OR7E37P	up	1,779814	Homo sapiens olfactory receptor, family 7, subfamily E, member 37 pseudogene (OR7E37P), non-coding RNA [NR_002163]
CLMN	up	1,775951	Homo sapiens calmin (calponin-like, transmembrane) (CLMN), mRNA [NM_024734]
SLC2A3	up	1,771033	Homo sapiens solute carrier family 2 (facilitated glucose transporter), member 3 (SLC2A3), mRNA [NM_006931]

HIATL1	up	1,770683	Homo sapiens hippocampus abundant transcript-like 1 (HIATL1), mRNA [NM_032558]
TMEM123	up	1,759982	Homo sapiens transmembrane protein 123 (TMEM123), mRNA [NM_052932]
DGKD	up	1,75765	Homo sapiens diacylglycerol kinase, delta 130kDa (DGKD), transcript variant 2, mRNA [NM_152879]
HCG21	up	1,757144	HLA complex group 21 (non-protein coding) [Source:HGNC Symbol;Acc:31335] [ENST00000419481]
ARID1B	up	1,747674	Homo sapiens AT rich interactive domain 1B (SWI1-like) (ARID1B), transcript variant 1, mRNA [NM_017519]
HSD17B12	up	1,728885	Homo sapiens hydroxysteroid (17-beta) dehydrogenase 12 (HSD17B12), mRNA [NM_016142]
CTSZ	up	1,728265	Homo sapiens cathepsin Z (CTSZ), mRNA [NM_001336]
CANX	up	1,727774	Homo sapiens calnexin (CANX), transcript variant 1, mRNA [NM_001746]
WDFY1	up	1,722954	Homo sapiens WD repeat and FYVE domain containing 1 (WDFY1), mRNA [NM_020830]
BTG1	up	1,721805	Homo sapiens B-cell translocation gene 1, anti-proliferative (BTG1), mRNA [NM_001731]
C1QTNF1	up	1,717557	Homo sapiens C1q and tumor necrosis factor related protein 1 (C1QTNF1), mRNA [NM_198594]
RGS20	up	1,716079	Homo sapiens regulator of G-protein signaling 20 (RGS20), transcript variant 1, mRNA [NM_170587]
REXO2	up	1,714904	Homo sapiens REX2, RNA exonuclease 2 homolog (S, cerevisiae) (REXO2), mRNA [NM_015523]
GRAMD4	up	1,709853	Homo sapiens GRAM domain containing 4 (GRAMD4), mRNA [NM_015124]
MAP2K1	up	1,707593	Homo sapiens mitogen-activated protein kinase kinase 1 (MAP2K1), mRNA [NM_002755]
GATA3	up	1,70627	Homo sapiens GATA binding protein 3 (GATA3), transcript variant 1, mRNA [NM_001002295]
KDM5B	up	1,703357	Homo sapiens lysine (K)-specific demethylase 5B (KDM5B), mRNA [NM_006618]
FCGR2C	up	1,699609	Homo sapiens Fc fragment of IgG, low affinity IIc, receptor for (CD32) (gene/pseudogene) (FCGR2C), mRNA [NM_201563]
ARHGEF7	up	1,696257	Homo sapiens Rho guanine nucleotide exchange factor (GEF) 7 (ARHGEF7), transcript variant 1, mRNA [NM_003899]
BBS9	up	1,69502	Homo sapiens Bardet-Biedl syndrome 9 (BBS9), transcript variant 2, mRNA [NM_198428]
FCGR2B	up	1,694052	Homo sapiens Fc fragment of IgG, low affinity IIb, receptor (CD32) (FCGR2B), transcript variant 1, mRNA [NM_004001]
PDXDC1	up	1,693408	Homo sapiens pyridoxal-dependent decarboxylase domain containing 1 (PDXDC1), mRNA [NM_015027]
WASH3P	up	1,690922	Homo sapiens CXYorf1-related protein, mRNA (cDNA clone IMAGE:4594516), partial cds, [BC067227]
DYNC1H1	up	1,689597	Homo sapiens dynein, cytoplasmic 1, heavy chain 1 (DYNC1H1), mRNA [NM_001376]
ZNF420	up	1,688304	Homo sapiens zinc finger protein 420 (ZNF420), mRNA [NM_144689]
LOC100288902	up	1,687849	PREDICTED: Homo sapiens hypothetical protein LOC100288902 (LOC100288902), mRNA [XM_002343872]
WRB	up	1,685375	Homo sapiens tryptophan rich basic protein (WRB), transcript variant 1, mRNA [NM_004627]

ITCH	up	1,675175	Homo sapiens itchy E3 ubiquitin protein ligase homolog (mouse) (ITCH), mRNA [NM_031483]
LOC348761	up	1,668629	Homo sapiens hypothetical LOC348761 (LOC348761), non-coding RNA [NR_033879]
KIAA1737	up	1,66855	Homo sapiens KIAA1737 (KIAA1737), mRNA [NM_033426]
LOC550112	up	1,664178	Homo sapiens hypothetical LOC550112 (LOC550112), non-coding RNA [NR_015439]
TBC1D1	up	1,658562	Homo sapiens TBC1 (tre-2/USP6, BUB2, cdc16) domain family, member 1 (TBC1D1), mRNA [NM_015173]
SPON2	up	1,654649	Homo sapiens spondin 2, extracellular matrix protein (SPON2), transcript variant 1, mRNA [NM_012445]
PHLDB1	up	1,654385	Homo sapiens pleckstrin homology-like domain, family B, member 1 (PHLDB1), transcript variant 1, mRNA [NM_015157]
TNIK	up	1,654161	Homo sapiens TRAF2 and NCK interacting kinase (TNIK), transcript variant 1, mRNA [NM_015028]
CLIC6	up	1,650523	Homo sapiens chloride intracellular channel 6 (CLIC6), nuclear gene encoding mitochondrial protein, mRNA [NM_053277]
PPA1	up	1,635471	Homo sapiens pyrophosphatase (inorganic) 1 (PPA1), mRNA [NM_021129]
ANKRD10	up	1,634782	Homo sapiens ankyrin repeat domain 10 (ANKRD10), mRNA [NM_017664]
TNIK	up	1,627616	Homo sapiens TRAF2 and NCK interacting kinase (TNIK), transcript variant 1, mRNA [NM_015028]
OSCP1	up	1,624839	Homo sapiens organic solute carrier partner 1 (OSCP1), transcript variant 2, mRNA [NM_206837]
WSB2	up	1,62385	Homo sapiens WD repeat and SOCS box containing 2 (WSB2), mRNA [NM_018639]
DHCR7	up	1,622801	Homo sapiens 7-dehydrocholesterol reductase (DHCR7), transcript variant 1, mRNA [NM_001360]
CBX6	up	1,622256	Homo sapiens chromobox homolog 6 (CBX6), mRNA [NM_014292]
LNP1	up	1,615841	Homo sapiens leukemia NUP98 fusion partner 1 (LNP1), mRNA [NM_001085451]
MORF4L2	up	1,611475	Homo sapiens mortality factor 4 like 2 (MORF4L2), transcript variant 2, mRNA [NM_012286]
PKNOX1	up	1,606846	Homo sapiens PBX/knotted 1 homeobox 1 (PKNOX1), mRNA [NM_004571]
HK2	up	1,599594	Homo sapiens hexokinase 2 (HK2), mRNA [NM_000189]
HEATR5B	up	1,599471	Homo sapiens HEAT repeat containing 5B (HEATR5B), mRNA [NM_019024]
CLIP1	up	1,598198	Homo sapiens CAP-GLY domain containing linker protein 1 (CLIP1), transcript variant 1, mRNA [NM_002956]
LRP12	up	1,590287	Homo sapiens low density lipoprotein receptor-related protein 12 (LRP12), transcript variant 1, mRNA [NM_013437]
ALDH18A1	up	1,58951	Homo sapiens aldehyde dehydrogenase 18 family, member A1 (ALDH18A1), nuclear gene encoding mitochondrial protein, transcript variant 1, mRNA [NM_002860]
SEC14L4	up	1,583403	Homo sapiens SEC14-like 4 (S, cerevisiae) (SEC14L4), transcript variant 1, mRNA [NM_174977]
KIF3A	up	1,58309	Homo sapiens kinesin family member 3A (KIF3A), mRNA [NM_007054]

ATXN1	up	1,579266	Homo sapiens ataxin 1 (ATXN1), transcript variant 1, mRNA [NM_000332]
COPA	up	1,575231	Homo sapiens coatomer protein complex, subunit alpha (COPA), transcript variant 1, mRNA [NM_001098398]
ZNF501	up	1,573577	Homo sapiens zinc finger protein 501 (ZNF501), mRNA [NM_145044]
SCARNA13	up	1,572956	Homo sapiens small Cajal body-specific RNA 13 (SCARNA13), guide RNA [NR_003002]
SLC37A1	up	1,567658	Homo sapiens solute carrier family 37 (glycerol-3-phosphate transporter), member 1 (SLC37A1), mRNA [NM_018964]
LRRC37B	up	1,566683	Homo sapiens leucine rich repeat containing 37B (LRRC37B), mRNA [NM_052888]
FLJ44715	up	1,565548	Homo sapiens cDNA FLJ44715 fis, clone BRACE3021430, [AK126671]
POLR2L	up	1,55615	Homo sapiens polymerase (RNA) II (DNA directed) polypeptide L, 7,6kDa (POLR2L), mRNA [NM_021128]
DYRK2	up	1,550518	Homo sapiens dual-specificity tyrosine-(Y)-phosphorylation regulated kinase 2 (DYRK2), transcript variant 2, mRNA [NM_006482]
USP48	up	1,543934	Homo sapiens ubiquitin specific peptidase 48 (USP48), transcript variant 2, mRNA [NM_001032730]
GRIN3B	up	1,541729	Homo sapiens glutamate receptor, ionotropic, N-methyl-D-aspartate 3B (GRIN3B), mRNA [NM_138690]
SENP8	up	1,540167	Homo sapiens SUMO/sentrin specific peptidase family member 8 (SENP8), transcript variant 2, mRNA [NM_145204]
IL21R	up	1,537761	Homo sapiens interleukin 21 receptor (IL21R), transcript variant 2, mRNA [NM_181078]
TMEM175	up	1,536947	Homo sapiens transmembrane protein 175 (TMEM175), mRNA [NM_032326]
RAB12	up	1,536497	Homo sapiens RAB12, member RAS oncogene family (RAB12), mRNA [NM_001025300]
KIAA1274	up	1,531204	Homo sapiens KIAA1274 (KIAA1274), mRNA [NM_014431]
NUDT13	up	1,531009	Homo sapiens nudix (nucleoside diphosphate linked moiety X)-type motif 13 (NUDT13), mRNA [NM_015901]
C7orf46	up	1,530887	Homo sapiens chromosome 7 open reading frame 46 (C7orf46), transcript variant 2, mRNA [NM_001127364]
PION	up	1,530066	Homo sapiens pigeon homolog (Drosophila) [Source:HGNC Symbol;Acc:28042] [ENST00000334003]
LANCL3	up	1,527162	Homo sapiens LanC lantibiotic synthetase component C-like 3 (bacterial) (LANCL3), transcript variant 1, mRNA [NM_198511]
WHSC2	up	1,52705	Homo sapiens Wolf-Hirschhorn syndrome candidate 2 (WHSC2), mRNA [NM_005663]
STAMBPL1	up	1,524766	Homo sapiens STAM binding protein-like 1 (STAMBPL1), mRNA [NM_020799]
SIK3	up	1,524044	Homo sapiens SIK family kinase 3 (SIK3), mRNA [NM_025164]
DHX32	up	1,522882	Homo sapiens DEAH (Asp-Glu-Ala-His) box polypeptide 32 (DHX32), mRNA [NM_018180]

FABP5	up	1,522808	Homo sapiens fatty acid binding protein 5 (psoriasis-associated) (FABP5), mRNA [NM_001444]
TMCC3	up	1,522248	Homo sapiens transmembrane and coiled-coil domain family 3 (TMCC3), mRNA [NM_020698]
ADA	up	1,521978	Homo sapiens adenosine deaminase (ADA), mRNA [NM_000022]
TES	up	1,521898	Homo sapiens testis derived transcript (3 LIM domains) (TES), transcript variant 2, mRNA [NM_152829]
RPL13	up	1,519837	Homo sapiens ribosomal protein L13 (RPL13), transcript variant 2, mRNA [NM_033251]
RUFY2	up	1,519119	Homo sapiens RUN and FYVE domain containing 2 [Source:HGNC Symbol;Acc:19761] [ENST00000342616]
UQCR10	up	1,51849	Homo sapiens ubiquinol-cytochrome c reductase, complex III subunit X (UQCR10), transcript variant 1, mRNA [NM_013387]
ZNF480	up	1,518458	Homo sapiens zinc finger protein 480 (ZNF480), mRNA [NM_144684]
UBE2O	up	1,518149	Homo sapiens ubiquitin-conjugating enzyme E2O (UBE2O), mRNA [NM_022066]
UGGT2	up	1,517916	Homo sapiens UDP-glucose glycoprotein glucosyltransferase 2 (UGGT2), mRNA [NM_020121]
FCRLB	up	1,513705	Homo sapiens Fc receptor-like B (FCRLB), mRNA [NM_001002901]
C2orf76	up	1,509502	Homo sapiens chromosome 2 open reading frame 76 (C2orf76), mRNA [NM_001017927]
FLJ11292	up	1,508443	Homo sapiens cDNA FLJ13355 fis, clone PLACE1000048, [AK023417]
RNF122	up	1,508419	Homo sapiens ring finger protein 122 (RNF122), mRNA [NM_024787]
GNAI3	up	1,50225	Homo sapiens guanine nucleotide binding protein (G protein), alpha inhibiting activity polypeptide 3 (GNAI3), mRNA [NM_006496]
FAM92A1	up	1,501227	Homo sapiens family with sequence similarity 92, member A1 (FAM92A1), mRNA [NM_145269]
FAM161A	up	1,500384	Homo sapiens family with sequence similarity 161, member A (FAM161A), transcript variant 2, mRNA [NM_032180]
PRRC2C	up	1,499136	Homo sapiens proline-rich coiled-coil 2C (PRRC2C), mRNA [NM_015172]
LOC401397	up	1,496497	Homo sapiens hypothetical LOC401397 (LOC401397), transcript variant 1, non-coding RNA [NR_024412]
CYCS	up	1,494383	Homo sapiens cytochrome c, somatic (CYCS), nuclear gene encoding mitochondrial protein, mRNA [NM_018947]
SC4MOL	up	1,490306	Homo sapiens sterol-C4-methyl oxidase-like (SC4MOL), transcript variant 1, mRNA [NM_006745]
PLCG2	up	1,488468	Homo sapiens phospholipase C, gamma 2 (phosphatidylinositol-specific) (PLCG2), mRNA [NM_002661]
RSPH3	up	1,488283	Homo sapiens radial spoke 3 homolog (Chlamydomonas) (RSPH3), mRNA [NM_031924]
C14orf49	up	1,487604	Homo sapiens chromosome 14 open reading frame 49 (C14orf49), mRNA [NM_152592]
YBX1	up	1,486195	Homo sapiens Y box binding protein 1 (YBX1), mRNA [NM_004559]

DLG1	up	1,484295	Homo sapiens discs, large homolog 1 (Drosophila) (DLG1), transcript variant 1, mRNA [NM_001098424]
SUB1	up	1,484063	Homo sapiens SUB1 homolog (S, cerevisiae) (SUB1), mRNA [NM_006713]
TMEM194B	up	1,483762	transmembrane protein 194B [Source:HGNC Symbol;Acc:33700] [ENST00000343105]
GPR180	up	1,482949	Homo sapiens G protein-coupled receptor 180 (GPR180), mRNA [NM_180989]
HDDC2	up	1,479761	HD domain containing 2 [Source:HGNC Symbol;Acc:21078] [ENST00000318787]
LOC148709	up	1,478376	Homo sapiens actin pseudogene (LOC148709), non-coding RNA [NR_002929]
FABP5	up	1,478082	Homo sapiens fatty acid binding protein 5 (psoriasis-associated) (FABP5), mRNA [NM_001444]
AGFG2	up	1,474533	Homo sapiens ArfGAP with FG repeats 2 (AGFG2), mRNA [NM_006076]
CDKN1A	up	1,474456	Homo sapiens cyclin-dependent kinase inhibitor 1A (p21, Cip1) (CDKN1A), transcript variant 2, mRNA [NM_078467]
RAB1A	up	1,473814	Homo sapiens RAB1A, member RAS oncogene family (RAB1A), transcript variant 1, mRNA [NM_004161]
TMED5	up	1,471673	Homo sapiens transmembrane emp24 protein transport domain containing 5 (TMED5), transcript variant 1, mRNA [NM_016040]
COG3	up	1,471225	Homo sapiens component of oligomeric golgi complex 3 (COG3), mRNA [NM_031431]
CAPZA1	up	1,468778	Homo sapiens capping protein (actin filament) muscle Z-line, alpha 1 (CAPZA1), mRNA [NM_006135]
CLIP4	up	1,468434	Homo sapiens CAP-GLY domain containing linker protein family, member 4 (CLIP4), mRNA [NM_024692]
FHL2	up	1,466287	Homo sapiens four and a half LIM domains 2 (FHL2), transcript variant 5, mRNA [NM_001039492]
PNPLA4	up	1,465633	Homo sapiens patatin-like phospholipase domain containing 4 (PNPLA4), transcript variant 1, mRNA [NM_004650]
CR2	up	1,465621	Homo sapiens complement component (3d/Epstein Barr virus) receptor 2 (CR2), transcript variant 1, mRNA [NM_001006658]
HCFC2	up	1,465305	Homo sapiens host cell factor C2 (HCFC2), mRNA [NM_013320]
ZNF430	up	1,464662	Homo sapiens zinc finger protein 430 (ZNF430), transcript variant 1, mRNA [NM_025189]
MTHFS	up	1,464158	Homo sapiens 5,10-methenyltetrahydrofolate synthetase (5-formyltetrahydrofolate cyclo-ligase) (MTHFS), transcript variant 1, mRNA [NM_006441]
MAGED1	up	1,463451	Homo sapiens melanoma antigen family D, 1 (MAGED1), transcript variant 1, mRNA [NM_001005333]
IDS	up	1,462598	Homo sapiens iduronate 2-sulfatase (IDS), transcript variant 2, mRNA [NM_006123]
HMGCS1	up	1,45986	Homo sapiens 3-hydroxy-3-methylglutaryl-CoA synthase 1 (soluble) (HMGCS1), transcript variant 2, mRNA [NM_002130]
SIDT1	up	1,452897	Homo sapiens SID1 transmembrane family, member 1 (SIDT1), mRNA [NM_017699]

LRRC42	up	1,449939	Homo sapiens leucine rich repeat containing 42 (LRRC42), mRNA [NM_052940]
ITGB7	up	1,449662	Homo sapiens integrin, beta 7 (ITGB7), mRNA [NM_000889]
CD53	up	1,448799	Homo sapiens CD53 molecule (CD53), transcript variant 1, mRNA [NM_001040033]
RABEPK	up	1,447279	Homo sapiens Rab9 effector protein with kelch motifs [Source:HGNC Symbol;Acc:16896] [ENST00000373544]
MLH1	up	1,44573	Homo sapiens mutL homolog 1, colon cancer, nonpolyposis type 2 (E, coli) (MLH1), transcript variant 1, mRNA [NM_000249]
HSD17B7	up	1,443416	Homo sapiens hydroxysteroid (17-beta) dehydrogenase 7 (HSD17B7), mRNA [NM_016371]
TTC3	up	1,443178	Homo sapiens tetratricopeptide repeat domain 3 (TTC3), transcript variant 1, mRNA [NM_003316]
MAP7	up	1,442169	Homo sapiens microtubule-associated protein 7 (MAP7), transcript variant 4, mRNA [NM_003980]
MEF2D	up	1,437441	Homo sapiens myocyte enhancer factor 2D (MEF2D), mRNA [NM_005920]
LOC285957	up	1,436779	Homo sapiens cDNA FLJ40207 fis, clone TESTI2020946, [AK097526]
TCEAL8	up	1,435627	Homo sapiens transcription elongation factor A (SII)-like 8 (TCEAL8), transcript variant 1, mRNA [NM_153333]
FABP5	up	1,435049	Homo sapiens fatty acid binding protein 5 (psoriasis-associated) (FABP5), mRNA [NM_001444]
RTN4	up	1,430342	Homo sapiens reticulon 4 (RTN4), transcript variant 1, mRNA [NM_020532]
TMED7-TICAM2	up	1,430279	Homo sapiens TMED7-TICAM2 readthrough (TMED7-TICAM2), transcript variant 1, mRNA [NM_001164468]
ARHGEF7	up	1,426558	Homo sapiens Rho guanine nucleotide exchange factor (GEF) 7 (ARHGEF7), transcript variant 1, mRNA [NM_003899]
LCOR	up	1,426356	Homo sapiens ligand dependent nuclear receptor corepressor (LCOR), transcript variant 1, mRNA [NM_032440]
LIPA	up	1,425564	Homo sapiens lipase A, lysosomal acid, cholesterol esterase (LIPA), transcript variant 2, mRNA [NM_000235]
HSD17B12	up	1,42467	Homo sapiens hydroxysteroid (17-beta) dehydrogenase 12 (HSD17B12), mRNA [NM_016142]
ZNF836	up	1,424367	Homo sapiens zinc finger protein 836 (ZNF836), mRNA [NM_001102657]
C15orf61	up	1,423074	Homo sapiens chromosome 15 open reading frame 61 (C15orf61), mRNA [NM_001143936]
ZNF383	up	1,419233	Homo sapiens zinc finger protein 383 (ZNF383), mRNA [NM_152604]
AVL9	up	1,418633	Homo sapiens AVL9 homolog (S, cerevisiase) (AVL9), mRNA [NM_015060]
LRRC37A3	up	1,415624	Homo sapiens leucine rich repeat containing 37, member A3 (LRRC37A3), mRNA [NM_199340]
SLC25A46	up	1,414246	Homo sapiens solute carrier family 25, member 46 (SLC25A46), mRNA [NM_138773]
NUDT4	up	1,413058	Homo sapiens nudix (nucleoside diphosphate linked moiety X)-type motif 4 (NUDT4), transcript variant 2, mRNA [NM_199040]

C21orf119	up	1,411079	Homo sapiens chromosome 21 open reading frame 119 (C21orf119), non-coding RNA [NR_026845]
PDP2	up	1,409007	Homo sapiens pyruvate dehyrogenase phosphatase catalytic subunit 2 (PDP2), mRNA [NM_020786]
EEF1E1	up	1,408755	Homo sapiens eukaryotic translation elongation factor 1 epsilon 1 (EEF1E1), transcript variant 1, mRNA [NM_004280]
USP48	up	1,404372	Homo sapiens ubiquitin specific peptidase 48 (USP48), transcript variant 1, mRNA [NM_032236]
KIAA1279	up	1,402715	Homo sapiens KIAA1279 (KIAA1279), nuclear gene encoding mitochondrial protein, mRNA [NM_015634]
HAAO	up	1,400751	Homo sapiens 3-hydroxyanthranilate 3,4-dioxygenase (HAAO), mRNA [NM_012205]
GCH1	up	1,400312	Homo sapiens GTP cyclohydrolase 1 (GCH1), transcript variant 4, mRNA [NM_001024071]
SNX5	up	1,399443	Homo sapiens sorting nexin 5 (SNX5), transcript variant 2, mRNA [NM_014426]
F11R	up	1,397312	Homo sapiens F11 receptor (F11R), mRNA [NM_016946]
RINT1	up	1,394614	Homo sapiens RAD50 interactor 1 (RINT1), mRNA [NM_021930]
PDHA1	up	1,393733	Homo sapiens pyruvate dehydrogenase (lipoamide) alpha 1 (PDHA1), nuclear gene encoding mitochondrial protein, transcript variant 1, mRNA [NM_000284]
YWHAG	up	1,392527	Homo sapiens tyrosine 3-monooxygenase/tryptophan 5-monooxygenase activation protein, gamma polypeptide (YWHAG), mRNA [NM_012479]
PPP1R13B	up	1,392016	Homo sapiens protein phosphatase 1, regulatory (inhibitor) subunit 13B (PPP1R13B), mRNA [NM_015316]
HCFC2	up	1,389742	Homo sapiens host cell factor C2 (HCFC2), mRNA [NM_013320]
SQLE	up	1,388906	Homo sapiens squalene epoxidase (SQLE), mRNA [NM_003129]
SCAF8	up	1,388327	Homo sapiens SR-related CTD-associated factor 8 (SCAF8), mRNA [NM_014892]
HPS1	up	1,388167	Homo sapiens Hermansky-Pudlak syndrome 1 (HPS1), transcript variant 3, mRNA [NM_182639]
LHPP	up	1,38798	Homo sapiens phospholysine phosphohistidine inorganic pyrophosphate phosphatase (LHPP), transcript variant 1, mRNA [NM_022126]
MCFD2	up	1,387632	Homo sapiens multiple coagulation factor deficiency 2 (MCFD2), transcript variant 1, mRNA [NM_139279]
TASP1	up	1,38413	Homo sapiens taspase, threonine aspartase, 1 (TASP1), mRNA [NM_017714]
SF3A1	up	1,38363	Homo sapiens splicing factor 3a, subunit 1, 120kDa (SF3A1), transcript variant 1, mRNA [NM_005877]
NSRP1	up	1,379925	Homo sapiens nuclear speckle splicing regulatory protein 1 (NSRP1), transcript variant 1, mRNA [NM_032141]
PIGY	up	1,379479	Homo sapiens phosphatidylinositol glycan anchor biosynthesis, class Y (PIGY), nuclear gene encoding mitochondrial protein, transcript variant 2, mRNA [NM_001042616]
GTPBP8	up	1,378469	Homo sapiens GTP-binding protein 8 (putative) (GTPBP8), transcript variant 1, mRNA [NM_014170]

SLC25A25	up	1,378103	Homo sapiens solute carrier family 25 (mitochondrial carrier; phosphate carrier), member 25 (SLC25A25), nuclear gene encoding mitochondrial protein, transcript variant 2, mRNA [NM_001006641]
FASN	up	1,377947	Homo sapiens fatty acid synthase (FASN), mRNA [NM_004104]
AKAP17A	up	1,376337	Homo sapiens A kinase (PRKA) anchor protein 17A (AKAP17A), transcript variant 1, mRNA [NM_005088]
RNF7	up	1,375908	Homo sapiens ring finger protein 7 (RNF7), transcript variant 5, non-coding RNA [NR_037702]
LOC650638	up	1,375028	PREDICTED: Homo sapiens signal recognition particle 54 kDa protein-like (LOC650638), mRNA [XM_939727]
CASC2	up	1,373392	Homo sapiens cancer susceptibility candidate 2 (CASC2), transcript variant 3, non-coding RNA [NR_026941]
COPG	up	1,372002	Homo sapiens coatomer protein complex, subunit gamma (COPG), mRNA [NM_016128]
NDUFB5	up	1,368224	Homo sapiens NADH dehydrogenase (ubiquinone) 1 beta subcomplex, 5, 16kDa (NDUFB5), nuclear gene encoding mitochondrial protein, transcript variant 3, mRNA [NM_001199958]
ZNF277	up	1,368096	Homo sapiens zinc finger protein 277 (ZNF277), mRNA [NM_021994]
C7orf46	up	1,366267	Homo sapiens chromosome 7 open reading frame 46 (C7orf46), transcript variant 1, mRNA [NM_199136]
RAP1A	up	1,365568	Homo sapiens RAP1A, member of RAS oncogene family (RAP1A), transcript variant 1, mRNA [NM_001010935]
CBR4	up	1,364642	Homo sapiens carbonyl reductase 4 (CBR4), mRNA [NM_032783]
TOR3A	up	1,364541	Homo sapiens torsin family 3, member A (TOR3A), mRNA [NM_022371]
WDR37	up	1,363727	Homo sapiens WD repeat domain 37 (WDR37), mRNA [NM_014023]
ZNF550	up	1,363422	zinc finger protein 550 [Source:HGNC Symbol;Acc:28643] [ENST00000447310]
SMCR7L	up	1,363274	Homo sapiens Smith-Magenis syndrome chromosome region, candidate 7-like (SMCR7L), nuclear gene encoding mitochondrial protein, mRNA [NM_019008]
CLEC2D	up	1,361906	Homo sapiens C-type lectin domain family 2, member D (CLEC2D), transcript variant 2, mRNA [NM_001004419]
SLC25A32	up	1,361707	Homo sapiens solute carrier family 25, member 32 (SLC25A32), nuclear gene encoding mitochondrial protein, mRNA [NM_030780]
PCNX	up	1,358492	Homo sapiens pecanex homolog (Drosophila) (PCNX), mRNA [NM_014982]
SKA2	up	1,358418	Homo sapiens spindle and kinetochore associated complex subunit 2 (SKA2), transcript variant 1, mRNA [NM_182620]
ZNF673	up	1,358221	Homo sapiens zinc finger family member 673 (ZNF673), transcript variant 3, mRNA [NM_001129899]
RPL34	up	1,35765	Homo sapiens ribosomal protein L34 (RPL34), transcript variant 2, mRNA [NM_033625]

PPP1R16B	up	1,357278	Homo sapiens protein phosphatase 1, regulatory (inhibitor) subunit 16B (PPP1R16B), transcript variant 1, mRNA [NM_015568]
FOXP1	up	1,357062	Homo sapiens forkhead box P1 (FOXP1), transcript variant 1, mRNA [NM_032682]
RPL15	up	1,35593	Homo sapiens ribosomal protein L15 (RPL15), mRNA [NM_002948]
HDDC2	up	1,355154	Homo sapiens HD domain containing 2 (HDDC2), mRNA [NM_016063]
RAB18	up	1,35352	Homo sapiens RAB18, member RAS oncogene family (RAB18), mRNA [NM_021252]
ACACA	up	1,352462	Homo sapiens acetyl-CoA carboxylase alpha (ACACA), transcript variant 2, mRNA [NM_198839]
THEM4	up	1,352103	Homo sapiens thioesterase superfamily member 4 (THEM4), mRNA [NM_053055]
IGBP1	up	1,350336	Homo sapiens immunoglobulin (CD79A) binding protein 1 (IGBP1), mRNA [NM_001551]
SDHB	up	1,34927	Homo sapiens succinate dehydrogenase complex, subunit B, iron sulfur (Ip) (SDHB), nuclear gene encoding mitochondrial protein, mRNA [NM_003000]
NPEPPS	up	1,348418	Homo sapiens aminopeptidase puromycin sensitive (NPEPPS), mRNA [NM_006310]
ZSCAN20	up	1,347975	Homo sapiens zinc finger and SCAN domain containing 20 (ZSCAN20), mRNA [NM_145238]
LOC401588	up	1,3477	Homo sapiens hypothetical LOC401588 (LOC401588), non-coding RNA [NR_015378]
SEC22B	up	1,347623	Homo sapiens SEC22 vesicle trafficking protein homolog B (S, cerevisiae) (gene/pseudogene) (SEC22B), mRNA [NM_004892]
CCDC102A	up	1,347322	Homo sapiens coiled-coil domain containing 102A (CCDC102A), mRNA [NM_033212]
FAM126A	up	1,347287	Homo sapiens family with sequence similarity 126, member A (FAM126A), mRNA [NM_032581]
NONO	up	1,347133	Homo sapiens non-POU domain containing, octamer-binding (NONO), transcript variant 2, mRNA [NM_007363]
TMED10	up	1,347001	Homo sapiens transmembrane emp24-like trafficking protein 10 (yeast) (TMED10), mRNA [NM_006827]
SCARNA16	up	1,346433	Homo sapiens small Cajal body-specific RNA 16 (SCARNA16), guide RNA [NR_003013]
VPS13C	up	1,344985	Homo sapiens vacuolar protein sorting 13 homolog C (S, cerevisiae) (VPS13C), transcript variant 2A, mRNA [NM_020821]
SURF4	up	1,344845	Homo sapiens surfeit 4 (SURF4), mRNA [NM_033161]
NSMAF	up	1,344809	Homo sapiens neutral sphingomyelinase (N-SMase) activation associated factor (NSMAF), transcript variant 1, mRNA [NM_003580]
HIPK3	up	1,342612	Homo sapiens homeodomain interacting protein kinase 3 (HIPK3), transcript variant 1, mRNA [NM_005734]
RAPGEF6	up	1,342051	Homo sapiens Rap guanine nucleotide exchange factor (GEF) 6 (RAPGEF6), transcript variant 2, mRNA [NM_016340]
MAP9	up	1,341276	Homo sapiens microtubule-associated protein 9 (MAP9), mRNA [NM_001039580]

RPL5	up	1,341131	Homo sapiens ribosomal protein L5 (RPL5), mRNA [NM_000969]
ATXN10	up	1,340986	Homo sapiens ataxin 10 (ATXN10), transcript variant 1, mRNA [NM_013236]
FAM96A	up	1,34028	Homo sapiens family with sequence similarity 96, member A (FAM96A), transcript variant 2, mRNA [NM_001014812]
GORASP2	up	1,339283	Homo sapiens golgi reassembly stacking protein 2, 55kDa (GORASP2), transcript variant 1, mRNA [NM_015530]
MTDH	up	1,339239	Homo sapiens metadherin (MTDH), mRNA [NM_178812]
ZWINT	up	1,337449	Homo sapiens ZW10 interactor (ZWINT), transcript variant 2, mRNA [NM_032997]
SLC2A1	up	1,336916	Homo sapiens solute carrier family 2 (facilitated glucose transporter), member 1 (SLC2A1), mRNA [NM_006516]
SLC25A46	up	1,334846	Homo sapiens solute carrier family 25, member 46 (SLC25A46), mRNA [NM_138773]
C19orf12	up	1,33478	Homo sapiens chromosome 19 open reading frame 12 (C19orf12), transcript variant 2, mRNA [NM_031448]
CCDC75	up	1,334404	Homo sapiens coiled-coil domain containing 75 (CCDC75), mRNA [NM_174931]
PLEKHM3	up	1,333847	Homo sapiens pleckstrin homology domain containing, family M, member 3 (PLEKHM3), mRNA [NM_001080475]
ABHD4	up	1,333558	Homo sapiens abhydrolase domain containing 4 (ABHD4), mRNA [NM_022060]
ZNF559	up	1,333228	Homo sapiens zinc finger protein 559 (ZNF559), transcript variant 2, mRNA [NM_032497]
ATF2	up	1,332088	Homo sapiens activating transcription factor 2 (ATF2), mRNA [NM_001880]
CHCHD7	up	1,331434	Homo sapiens coiled-coil-helix-coiled-coil-helix domain containing 7 (CHCHD7), transcript variant 1, mRNA [NM_001011667]
C12orf11	up	1,331393	Homo sapiens chromosome 12 open reading frame 11 (C12orf11), mRNA [NM_018164]
GMFB	up	1,330366	Homo sapiens glia maturation factor, beta (GMFB), mRNA [NM_004124]
MAN1A2	up	1,33025	Homo sapiens mannosidase, alpha, class 1A, member 2 (MAN1A2), mRNA [NM_006699]
TMEM60	up	1,329257	Homo sapiens transmembrane protein 60 (TMEM60), mRNA [NM_032936]
LEMD3	up	1,328129	Homo sapiens LEM domain containing 3 (LEMD3), transcript variant 1, mRNA [NM_014319]
FAM185A	up	1,327732	family with sequence similarity 185, member A [Source:HGNC Symbol;Acc:22412] [ENST00000496790]
GTF2E1	up	1,326827	Homo sapiens general transcription factor IIE, polypeptide 1, alpha 56kDa (GTF2E1), mRNA [NM_005513]
PPP1CC	up	1,326132	Homo sapiens protein phosphatase 1, catalytic subunit, gamma isozyme (PPP1CC), mRNA [NM_002710]
C7orf59	up	1,323726	Homo sapiens chromosome 7 open reading frame 59 (C7orf59), mRNA [NM_001008395]

LOC100128822	up	1,32238	Homo sapiens hypothetical LOC100128822 (LOC100128822), non-coding RNA [NR_027387]
PPP1CB	up	1,321882	Homo sapiens protein phosphatase 1, catalytic subunit, beta isozyme (PPP1CB), transcript variant 1, mRNA [NM_002709]
BRIX1	up	1,319586	Homo sapiens BRX1, biogenesis of ribosomes, homolog (S, cerevisiae) (BRIX1), mRNA [NM_018321]
HTATIP2	up	1,318579	Homo sapiens HIV-1 Tat interactive protein 2, 30kDa (HTATIP2), transcript variant 2, mRNA [NM_006410]
NUS1	up	1,318186	Homo sapiens nuclear undecaprenyl pyrophosphate synthase 1 homolog (S, cerevisiae) (NUS1), mRNA [NM_138459]
PLAA	up	1,318097	Homo sapiens phospholipase A2-activating protein (PLAA), mRNA [NM_001031689]
ETFDH	up	1,317333	Homo sapiens electron-transferring-flavoprotein dehydrogenase (ETFDH), nuclear gene encoding mitochondrial protein, mRNA [NM_004453]
WDR26	up	1,317146	Homo sapiens WD repeat domain 26 (WDR26), transcript variant 1, mRNA [NM_025160]
KIRREL	up	1,315505	Homo sapiens kin of IRRE like (Drosophila) (KIRREL), mRNA [NM_018240]
FAM53C	up	1,315467	Homo sapiens family with sequence similarity 53, member C (FAM53C), transcript variant 2, mRNA [NM_016605]
CPEB4	up	1,314939	Homo sapiens cytoplasmic polyadenylation element binding protein 4 (CPEB4), mRNA [NM_030627]
C2orf81	up	1,314167	Homo sapiens chromosome 2 open reading frame 81 (C2orf81), mRNA [NM_001145054]
LAX1	up	1,313687	Homo sapiens lymphocyte transmembrane adaptor 1 (LAX1), transcript variant 1, mRNA [NM_017773]
ARCN1	up	1,313662	Homo sapiens archain 1 (ARCN1), transcript variant 1, mRNA [NM_001655]
ADIPOR2	up	1,313271	Homo sapiens adiponectin receptor 2 (ADIPOR2), mRNA [NM_024551]
PCM1	up	1,31272	Homo sapiens pericentriolar material 1 (PCM1), mRNA [NM_006197]
ELF4	up	1,312552	Homo sapiens E74-like factor 4 (ets domain transcription factor) (ELF4), transcript variant 1, mRNA [NM_001421]
CBWD5	up	1,311987	Homo sapiens COBW domain containing 5 (CBWD5), mRNA [NM_001024916]
WHSC1	up	1,310959	Homo sapiens Wolf-Hirschhorn syndrome candidate 1 (WHSC1), transcript variant 8, mRNA [NM_007331]
MRPL33	up	1,310922	Homo sapiens mitochondrial ribosomal protein L33 (MRPL33), nuclear gene encoding mitochondrial protein, transcript variant 1, mRNA [NM_004891]
SPG11	up	1,309791	Homo sapiens spastic paraplegia 11 (autosomal recessive) (SPG11), transcript variant 1, mRNA [NM_025137]
MPHOSPH10	up	1,30966	Homo sapiens M-phase phosphoprotein 10 (U3 small nucleolar ribonucleoprotein) (MPHOSPH10), mRNA [NM_005791]
SLMAP	up	1,309379	Homo sapiens sarcolemma associated protein (SLMAP), mRNA [NM_007159]

AZIN1	up	1,307788	Homo sapiens antizyme inhibitor 1 (AZIN1), transcript variant 1, mRNA [NM_015878]
RAB5A	up	1,307608	Homo sapiens RAB5A, member RAS oncogene family (RAB5A), mRNA [NM_004162]
SRP19	up	1,306572	Homo sapiens signal recognition particle 19kDa (SRP19), transcript variant 1, mRNA [NM_003135]
LOC642852	up	1,306136	Homo sapiens hypothetical LOC642852 (LOC642852), non-coding RNA [NR_026943]
FAM27E3	up	1,305136	PREDICTED: Homo sapiens family with sequence similarity 27, member E3 (FAM27E3), mRNA [XM_001720463]
HIAT1	up	1,304669	Homo sapiens hippocampus abundant transcript 1 (HIAT1), mRNA [NM_033055]
CHCHD2	up	1,303494	Homo sapiens coiled-coil-helix-coiled-coil-helix domain containing 2 (CHCHD2), nuclear gene encoding mitochondrial protein, mRNA [NM_016139]
MTIF2	up	1,303352	Homo sapiens mitochondrial translational initiation factor 2 (MTIF2), nuclear gene encoding mitochondrial protein, transcript variant 1, mRNA [NM_001005369]
MBD1	up	1,303249	Homo sapiens methyl-CpG binding domain protein 1 (MBD1), transcript variant 13, mRNA [NM_001204151]
UQCC	up	1,302508	Homo sapiens ubiquinol-cytochrome c reductase complex chaperone (UQCC), nuclear gene encoding mitochondrial protein, transcript variant 1, mRNA [NM_018244]
ITFG1	up	1,301487	Homo sapiens integrin alpha FG-GAP repeat containing 1 (ITFG1), mRNA [NM_030790]
KDM4C	up	1,299273	Homo sapiens lysine (K)-specific demethylase 4C (KDM4C), transcript variant 1, mRNA [NM_015061]
LRSAM1	up	1,299201	Homo sapiens leucine rich repeat and sterile alpha motif containing 1 (LRSAM1), transcript variant 1, mRNA [NM_138361]
UPF2	up	1,299071	Homo sapiens UPF2 regulator of nonsense transcripts homolog (yeast) (UPF2), transcript variant 1, mRNA [NM_080599]
BAZ2B	up	1,298568	Homo sapiens bromodomain adjacent to zinc finger domain, 2B (BAZ2B), mRNA [NM_013450]
PCNP	up	1,297864	Homo sapiens PEST proteolytic signal containing nuclear protein (PCNP), mRNA [NM_020357]
IDS	up	1,297775	Homo sapiens iduronate 2-sulfatase (IDS), transcript variant 2, mRNA [NM_006123]
CWC22	up	1,297725	Homo sapiens CWC22 spliceosome-associated protein homolog (S, cerevisiae) (CWC22), mRNA [NM_020943]
IL2RB	up	1,29772	Homo sapiens interleukin 2 receptor, beta (IL2RB), mRNA [NM_000878]
POLR3E	up	1,297422	Homo sapiens polymerase (RNA) III (DNA directed) polypeptide E (80kD) (POLR3E), mRNA [NM_018119]
ACTR3B	up	1,29431	Homo sapiens ARP3 actin-related protein 3 homolog B (yeast) (ACTR3B), transcript variant 1, mRNA [NM_020445]
DLG1	up	1,29349	Homo sapiens discs, large homolog 1 (Drosophila) (DLG1), transcript variant 2, mRNA [NM_004087]
TNRC6C	up	1,293402	Homo sapiens trinucleotide repeat containing 6C (TNRC6C), transcript variant 1, mRNA [NM_001142640]

F11R	up	1,293206	Homo sapiens F11 receptor (F11R), mRNA [NM_016946]
DDX23	up	1,293064	Homo sapiens DEAD (Asp-Glu-Ala-Asp) box polypeptide 23 (DDX23), mRNA [NM_004818]
ZNF295	up	1,291837	Homo sapiens zinc finger protein 295 (ZNF295), transcript variant 2, mRNA [NM_020727]
EIF1B	up	1,29076	Homo sapiens eukaryotic translation initiation factor 1B (EIF1B), mRNA [NM_005875]
LOC100129478	up	1,290153	PREDICTED: Homo sapiens uncharacterized protein C4orf46-like (LOC100129478), mRNA [XM_001719774]
BCL2L13	up	1,28962	Homo sapiens BCL2-like 13 (apoptosis facilitator) (BCL2L13), nuclear gene encoding mitochondrial protein, mRNA [NM_015367]
SRP54	up	1,287985	Homo sapiens signal recognition particle 54kDa (SRP54), transcript variant 1, mRNA [NM_003136]
YIPF5	up	1,287893	Homo sapiens Yip1 domain family, member 5 (YIPF5), transcript variant 1, mRNA [NM_001024947]
FRYL	up	1,285939	Homo sapiens FRY-like (FRYL), mRNA [NM_015030]
PNRC1	up	1,285076	Homo sapiens proline-rich nuclear receptor coactivator 1 (PNRC1), mRNA [NM_006813]
TNNI3	up	1,284091	Homo sapiens troponin I type 3 (cardiac) (TNNI3), mRNA [NM_000363]
SP110	up	1,283614	Homo sapiens SP110 nuclear body protein (SP110), transcript variant b, mRNA [NM_004510]
SUPT5H	up	1,280528	Homo sapiens suppressor of Ty 5 homolog (S, cerevisiae) (SUPT5H), transcript variant 1, mRNA [NM_003169]
ZNF561	up	1,280467	Homo sapiens zinc finger protein 561 (ZNF561), mRNA [NM_152289]
DTWD1	up	1,280101	Homo sapiens DTW domain containing 1 (DTWD1), transcript variant 1, mRNA [NM_020234]
C15orf24	up	1,279725	Homo sapiens chromosome 15 open reading frame 24 (C15orf24), mRNA [NM_020154]
SLC30A7	up	1,279567	Homo sapiens solute carrier family 30 (zinc transporter), member 7 (SLC30A7), transcript variant 1, mRNA [NM_133496]
PNPLA8	up	1,277379	Homo sapiens patatin-like phospholipase domain containing 8 (PNPLA8), mRNA [NM_015723]
DHX57	up	1,274888	Homo sapiens DEAH (Asp-Glu-Ala-Asp/His) box polypeptide 57 (DHX57), mRNA [NM_198963]
KRBA2	up	1,274885	Homo sapiens KRAB-A domain containing 2 (KRBA2), mRNA [NM_213597]
PDCD2	up	1,274075	Homo sapiens programmed cell death 2 (PDCD2), transcript variant 1, mRNA [NM_002598]
TPST2	up	1,273951	Homo sapiens tyrosylprotein sulfotransferase 2 (TPST2), transcript variant 1, mRNA [NM_001008566]
MYO9A	up	1,273111	Homo sapiens myosin IXA (MYO9A), mRNA [NM_006901]
POLI	up	1,272953	Homo sapiens polymerase (DNA directed) iota (POLI), mRNA [NM_007195]
TMEM184C	up	1,271928	Homo sapiens transmembrane protein 184C (TMEM184C), mRNA [NM_018241]
TMEM97	up	1,271359	Homo sapiens transmembrane protein 97 (TMEM97), mRNA [NM_014573]
TMEM14C	up	1,271159	Homo sapiens transmembrane protein 14C (TMEM14C), transcript variant 2, mRNA [NM_016462]

AKIRIN1	up	1,270786	Homo sapiens akirin 1 (AKIRIN1), transcript variant 1, mRNA [NM_024595]
EP400	up	1,270381	Homo sapiens E1A binding protein p400 (EP400), mRNA [NM_015409]
PAK1IP1	up	1,270094	Homo sapiens PAK1 interacting protein 1 (PAK1IP1), mRNA [NM_017906]
PPIP5K2	up	1,269374	Homo sapiens diphosphoinositol pentakisphosphate kinase 2 (PPIP5K2), mRNA [NM_015216]
BTF3P11	up	1,269284	Homo sapiens basic transcription factor 3 pseudogene 11 (BTF3P11), non-coding RNA [NR_026983]
POLR1D	up	1,269136	Homo sapiens polymerase (RNA) I polypeptide D, 16kDa (POLR1D), transcript variant 2, mRNA [NM_152705]
PFKM	up	1,26849	Homo sapiens phosphofructokinase, muscle (PFKM), transcript variant 4, mRNA [NM_000289]
DDX26B	up	1,267719	Homo sapiens DEAD/H (Asp-Glu-Ala-Asp/His) box polypeptide 26B (DDX26B), mRNA [NM_182540]
ANKRD46	up	1,265539	Homo sapiens ankyrin repeat domain 46 (ANKRD46), mRNA [NM_198401]
SEC24B	up	1,263895	Homo sapiens SEC24 family, member B (S, cerevisiae) (SEC24B), transcript variant 1, mRNA [NM_006323]
LAMTOR3	up	1,263621	Homo sapiens late endosomal/lysosomal adaptor, MAPK and MTOR activator 3 (LAMTOR3), transcript variant 1, mRNA [NM_021970]
TATDN3	up	1,262408	Homo sapiens TatD DNase domain containing 3 (TATDN3), transcript variant 5, mRNA [NM_001146171]
RAB28	up	1,260732	Homo sapiens RAB28, member RAS oncogene family (RAB28), transcript variant 1, mRNA [NM_001017979]
RNF7	up	1,260611	Homo sapiens ring finger protein 7 (RNF7), transcript variant 1, mRNA [NM_014245]
CUL5	up	1,259617	Homo sapiens cullin 5 (CUL5), mRNA [NM_003478]
SNX3	up	1,259305	Homo sapiens sorting nexin 3 (SNX3), transcript variant 1, mRNA [NM_003795]
HSD17B7	up	1,258579	Homo sapiens hydroxysteroid (17-beta) dehydrogenase 7 (HSD17B7), mRNA [NM_016371]
SETD5	up	1,257803	Homo sapiens SET domain containing 5 (SETD5), mRNA [NM_001080517]
SMG1	up	1,25688	Homo sapiens smg-1 homolog, phosphatidylinositol 3-kinase-related kinase (C, elegans) (SMG1), mRNA [NM_015092]
POLR2K	up	1,256868	Homo sapiens polymerase (RNA) II (DNA directed) polypeptide K, 7,0kDa (POLR2K), mRNA [NM_005034]
GNPDA2	up	1,256638	Homo sapiens glucosamine-6-phosphate deaminase 2 (GNPDA2), mRNA [NM_138335]
KIAA1429	up	1,256547	Homo sapiens KIAA1429 (KIAA1429), transcript variant 1, mRNA [NM_015496]
TTC19	up	1,256492	Homo sapiens tetratricopeptide repeat domain 19 (TTC19), transcript variant 1, mRNA [NM_017775]
UBE2H	up	1,255497	Homo sapiens ubiquitin-conjugating enzyme E2H (UBE2H), transcript variant 1, mRNA [NM_003344]
IVD	up	1,254585	Homo sapiens isovaleryl-CoA dehydrogenase (IVD), nuclear gene encoding mitochondrial protein, transcript variant 2, mRNA [NM_001159508]

C20orf24	up	1,252327	Homo sapiens chromosome 20 open reading frame 24 (C20orf24), transcript variant 1, mRNA [NM_018840]
RAB3GAP2	up	1,252313	Homo sapiens RAB3 GTPase activating protein subunit 2 (non-catalytic) (RAB3GAP2), mRNA [NM_012414]
TSSC1	up	1,251099	Homo sapiens tumor suppressing subtransferable candidate 1 (TSSC1), mRNA [NM_003310]
DDX6	up	1,249882	Homo sapiens DEAD (Asp-Glu-Ala-Asp) box polypeptide 6 (DDX6), mRNA [NM_004397]
SLC31A1	up	1,249484	Homo sapiens solute carrier family 31 (copper transporters), member 1 (SLC31A1), mRNA [NM_001859]
NT5DC1	up	1,249322	Homo sapiens 5'-nucleotidase domain containing 1 (NT5DC1), mRNA [NM_152729]
PPP1CC	up	1,249076	Homo sapiens protein phosphatase 1, catalytic subunit, gamma isozyme (PPP1CC), mRNA [NM_002710]
HAAO	up	1,248629	Homo sapiens 3-hydroxyanthranilate 3,4-dioxygenase (HAAO), mRNA [NM_012205]
DOCK2	up	1,247425	Homo sapiens dedicator of cytokinesis 2 (DOCK2), mRNA [NM_004946]
SLC35B3	up	1,247412	Homo sapiens solute carrier family 35, member B3 (SLC35B3), transcript variant 1, mRNA [NM_015948]
PPP1CB	up	1,247157	Homo sapiens protein phosphatase 1, catalytic subunit, beta isozyme (PPP1CB), transcript variant 1, mRNA [NM_002709]
GIPR	up	1,247111	Homo sapiens gastric inhibitory polypeptide receptor (GIPR), mRNA [NM_000164]
C14orf1	up	1,246776	Homo sapiens chromosome 14 open reading frame 1 (C14orf1), mRNA [NM_007176]
EIF3M	up	1,245997	Homo sapiens eukaryotic translation initiation factor 3, subunit M (EIF3M), mRNA [NM_006360]
RPS3A	up	1,245926	Homo sapiens ribosomal protein S3A (RPS3A), mRNA [NM_001006]
HAGH	up	1,245403	Homo sapiens hydroxyacylglutathione hydrolase (HAGH), transcript variant 2, mRNA [NM_001040427]
NIN	up	1,245146	Homo sapiens ninein (GSK3B interacting protein) (NIN), transcript variant 6, mRNA [NM_016350]
SRP9	up	1,244263	Homo sapiens signal recognition particle 9kDa (SRP9), transcript variant 2, mRNA [NM_003133]
TRA2B	up	1,244013	Homo sapiens transformer 2 beta homolog (Drosophila) (TRA2B), mRNA [NM_004593]
SNX3	up	1,243886	Homo sapiens sorting nexin 3 (SNX3), transcript variant 1, mRNA [NM_003795]
TYW1	up	1,243483	Homo sapiens tRNA-yW synthesizing protein 1 homolog (S, cerevisiae) (TYW1), mRNA [NM_018264]
RPAIN	up	1,242647	Homo sapiens RPA interacting protein (RPAIN), transcript variant 5, mRNA [NM_001160266]
METTL21A	up	1,241908	Homo sapiens methyltransferase like 21A (METTL21A), transcript variant 2, mRNA [NM_001127395]
ATP6V1F	up	1,240448	Homo sapiens ATPase, H+ transporting, lysosomal 14kDa, V1 subunit F (ATP6V1F), transcript variant 1, mRNA [NM_004231]

PPP1R12A	up	1,238642	Homo sapiens protein phosphatase 1, regulatory (inhibitor) subunit 12A (PPP1R12A), transcript variant 1, mRNA [NM_002480]
FAM104A	up	1,238432	Homo sapiens family with sequence similarity 104, member A (FAM104A), transcript variant 2, mRNA [NM_032837]
ATF6	up	1,236805	Homo sapiens activating transcription factor 6 (ATF6), mRNA [NM_007348]
WDR11	up	1,235258	Homo sapiens WD repeat domain 11 (WDR11), mRNA [NM_018117]
IFNAR2	up	1,234499	Homo sapiens interferon (alpha, beta and omega) receptor 2 (IFNAR2), transcript variant 2, mRNA [NM_000874]
LOC493754	up	1,23294	Homo sapiens RAB guanine nucleotide exchange factor (GEF) 1 pseudogene (LOC493754), non-coding RNA [NR_002933]
LOC100506105	up	1,231891	PREDICTED: Homo sapiens hypothetical LOC100506105 (LOC100506105), partial miscRNA [XR_109318]
MRPL19	up	1,231809	Homo sapiens mitochondrial ribosomal protein L19 (MRPL19), nuclear gene encoding mitochondrial protein, mRNA [NM_014763]
UTP11L	up	1,231127	Homo sapiens UTP11-like, U3 small nucleolar ribonucleoprotein, (yeast) (UTP11L), mRNA [NM_016037]
NRBF2	up	1,230722	Homo sapiens nuclear receptor binding factor 2 (NRBF2), mRNA [NM_030759]
GALC	up	1,228685	Homo sapiens galactosylceramidase (GALC), transcript variant 1, mRNA [NM_000153]
C9orf85	up	1,228393	Homo sapiens chromosome 9 open reading frame 85 (C9orf85), mRNA [NM_182505]
FNTA	up	1,228101	Homo sapiens farnesyltransferase, CAAX box, alpha (FNTA), transcript variant 1, mRNA [NM_002027]
SCN4B	up	1,227949	Homo sapiens sodium channel, voltage-gated, type IV, beta (SCN4B), transcript variant 1, mRNA [NM_174934]
RTCD1	up	1,227806	Homo sapiens RNA terminal phosphate cyclase domain 1 (RTCD1), transcript variant 2, mRNA [NM_003729]
CSRNP1	up	1,227121	Homo sapiens cysteine-serine-rich nuclear protein 1 (CSRNP1), mRNA [NM_033027]
PPIAL4G	up	1,226875	Homo sapiens cDNA FLJ16804 fis, clone TESTI4020460, highly similar to Peptidyl-prolyl cis-trans isomerase A (EC 5,2,1,8), [AK123006]
TH1L	up	1,226557	Homo sapiens TH1-like (Drosophila) (TH1L), transcript variant 1, mRNA [NM_198976]
LASP1	up	1,22633	Homo sapiens LIM and SH3 protein 1 (LASP1), mRNA [NM_006148]
CDKN1A	up	1,225608	Homo sapiens tumor protein p53 binding protein 1 (TP53BP1), transcript variant 3, mRNA [NM_005657]
CCDC72	up	1,224336	coiled-coil domain containing 72 [Source:HGNC Symbol;Acc:26932] [ENST00000477624]
EIF3E	up	1,222919	Homo sapiens eukaryotic translation initiation factor 3, subunit E (EIF3E), mRNA [NM_001568]

PSMD6	up	1,222527	Homo sapiens proteasome (prosome, macropain) 26S subunit, non-ATPase, 6 (PSMD6), mRNA [NM_014814]
SDCCAG3	up	1,222194	Homo sapiens serologically defined colon cancer antigen 3 (SDCCAG3), transcript variant 1, mRNA [NM_001039707]
NDUFA1	up	1,219971	Homo sapiens NADH dehydrogenase (ubiquinone) 1 alpha subcomplex, 1, 7,5kDa (NDUFA1), nuclear gene encoding mitochondrial protein, mRNA [NM_004541]
KIAA1430	up	1,219692	Homo sapiens KIAA1430 (KIAA1430), mRNA [NM_020827]
NKTR	up	1,21817	Homo sapiens natural killer-tumor recognition sequence (NKTR), mRNA [NM_005385]
TOMM20	up	1,217494	Homo sapiens translocase of outer mitochondrial membrane 20 homolog (yeast) (TOMM20), nuclear gene encoding mitochondrial protein, mRNA [NM_014765]
NBR1	up	1,215449	Homo sapiens neighbor of BRCA1 gene 1 (NBR1), transcript variant 2, mRNA [NM_031858]
YPEL5	up	1,214824	Homo sapiens yippee-like 5 (Drosophila) (YPEL5), transcript variant 4, mRNA [NM_016061]
TMEM128	up	1,214742	Homo sapiens transmembrane protein 128 (TMEM128), mRNA [NM_032927]
GLIPR1	up	1,21448	Homo sapiens GLI pathogenesis-related 1 (GLIPR1), mRNA [NM_006851]
IQCB1	up	1,214175	Homo sapiens IQ motif containing B1 (IQCB1), transcript variant 3, mRNA [NM_001023571]
GTF2A2	up	1,213691	Homo sapiens general transcription factor IIA, 2, 12kDa (GTF2A2), mRNA [NM_004492]
LTN1	up	1,212665	Homo sapiens listerin E3 ubiquitin protein ligase 1 (LTN1), mRNA [NM_015565]
TMEM170A	up	1,212086	Homo sapiens transmembrane protein 170A (TMEM170A), mRNA [NM_145254]
DDX59	up	1,211403	Homo sapiens DEAD (Asp-Glu-Ala-Asp) box polypeptide 59 (DDX59), mRNA [NM_001031725]
TGFBRAP1	up	1,210736	transforming growth factor, beta receptor associated protein 1 [Source:HGNC Symbol;Acc:16836] [ENST00000393359]
NAA15	up	1,210082	Homo sapiens N(alpha)-acetyltransferase 15, NatA auxiliary subunit (NAA15), mRNA [NM_057175]
GPATCH8	up	1,209991	Homo sapiens G patch domain containing 8 (GPATCH8), transcript variant 1, mRNA [NM_001002909]
NANS	up	1,20951	Homo sapiens N-acetylneuraminic acid synthase (NANS), mRNA [NM_018946]
ERGIC2	up	1,20943	Homo sapiens ERGIC and golgi 2 (ERGIC2), mRNA [NM_016570]
TOR3A	up	1,20918	Homo sapiens torsin family 3, member A (TOR3A), mRNA [NM_022371]
NLK	up	1,208933	Homo sapiens nemo-like kinase (NLK), mRNA [NM_016231]
RBBP4	up	1,208777	Homo sapiens retinoblastoma binding protein 4 (RBBP4), transcript variant 1, mRNA [NM_005610]
AK3	up	1,208613	Homo sapiens adenylate kinase 3 (AK3), nuclear gene encoding mitochondrial protein, transcript variant 1, mRNA [NM_016282]
TCEB1P3	up	1,208285	Homo sapiens transcription elongation factor B (SIII), polypeptide 1 pseudogene 3 [Source:HGNC Symbol;Acc:29457] [ENST00000535261]

SMPD1	up	1,208181	Homo sapiens sphingomyelin phosphodiesterase 1, acid lysosomal (SMPD1), transcript variant 1, mRNA [NM_000543]
LOC399753	up	1,208045	Homo sapiens cDNA FLJ14094 fis, clone MAMMA1000372, [AK024156]
FTSJ1	up	1,207767	Homo sapiens FtsJ homolog 1 (E, coli) (FTSJ1), transcript variant 3, mRNA [NM_177439]
MYOD1	up	1,207563	Homo sapiens myogenic differentiation 1 (MYOD1), mRNA [NM_002478]
PFKP	up	1,205894	Homo sapiens phosphofructokinase, platelet (PFKP), transcript variant 1, mRNA [NM_002627]
APOM	up	1,204504	Homo sapiens apolipoprotein M (APOM), mRNA [NM_019101]
RER1	up	1,203958	Homo sapiens RER1 retention in endoplasmic reticulum 1 homolog (S, cerevisiae) (RER1), mRNA [NM_007033]
HOXD9	up	1,20339	Homo sapiens homeobox D9 (HOXD9), mRNA [NM_014213]
PHACTR2	up	1,197495	Homo sapiens phosphatase and actin regulator 2 (PHACTR2), transcript variant 3, mRNA [NM_014721]
PCBP2	up	1,197484	Homo sapiens poly(rC) binding protein 2 (PCBP2), transcript variant 2, mRNA [NM_031989]
DPH5	up	1,196702	Homo sapiens DPH5 homolog (S, cerevisiae) (DPH5), transcript variant 1, mRNA [NM_001077394]
TRIP12	up	1,194401	Homo sapiens thyroid hormone receptor interactor 12 (TRIP12), mRNA [NM_004238]
SLC41A2	up	1,193007	Homo sapiens solute carrier family 41, member 2 (SLC41A2), mRNA [NM_032148]
PDPK1	up	1,192244	Homo sapiens 3-phosphoinositide dependent protein kinase-1 (PDPK1), transcript variant 1, mRNA [NM_002613]
EBNA1BP2	up	1,191935	Homo sapiens EBNA1 binding protein 2 (EBNA1BP2), transcript variant 2, mRNA [NM_006824]
PHIP	up	1,191372	Homo sapiens pleckstrin homology domain interacting protein (PHIP), mRNA [NM_017934]
PDCD2	up	1,191118	Homo sapiens programmed cell death 2 (PDCD2), transcript variant 1, mRNA [NM_002598]
C2orf3	up	1,190678	Homo sapiens chromosome 2 open reading frame 3 (C2orf3), transcript variant 1, mRNA [NM_003203]
RELT	up	1,190049	Homo sapiens RELT tumor necrosis factor receptor (RELT), transcript variant 1, mRNA [NM_032871]
COX15	up	1,188925	Homo sapiens COX15 homolog, cytochrome c oxidase assembly protein (yeast) (COX15), nuclear gene encoding mitochondrial protein, transcript variant 1, mRNA [NM_078470]
ST13	up	1,188555	Homo sapiens suppression of tumorigenicity 13 (colon carcinoma) (Hsp70 interacting protein) (ST13), mRNA [NM_003932]
ABCE1	up	1,188089	Homo sapiens ATP-binding cassette, sub-family E (OABP), member 1 (ABCE1), transcript variant 1, mRNA [NM_002940]
FAM174A	up	1,187819	Homo sapiens family with sequence similarity 174, member A (FAM174A), mRNA [NM_198507]
WDR75	up	1,187804	Homo sapiens WD repeat domain 75 (WDR75), mRNA [NM_032168]
MARCH7	up	1,186205	Homo sapiens membrane-associated ring finger (C3HC4) 7 (MARCH7), mRNA [NM_022826]

PPP1R12A	up	1,186007	Homo sapiens protein phosphatase 1, regulatory (inhibitor) subunit 12A (PPP1R12A), transcript variant 1, mRNA [NM_002480]
PEX3	up	1,185974	Homo sapiens peroxisomal biogenesis factor 3 (PEX3), mRNA [NM_003630]
CMAS	up	1,184666	Homo sapiens cytidine monophosphate N-acetylneuraminic acid synthetase (CMAS), mRNA [NM_018686]
CIR1	up	1,184286	Homo sapiens corepressor interacting with RBPJ, 1 (CIR1), mRNA [NM_004882]
YME1L1	up	1,183936	Homo sapiens YME1-like 1 (S, cerevisiae) (YME1L1), nuclear gene encoding mitochondrial protein, transcript variant 1, mRNA [NM_139312]
LRRC47	up	1,183005	Homo sapiens leucine rich repeat containing 47 (LRRC47), mRNA [NM_020710]
ANKRD17	up	1,182324	Homo sapiens ankyrin repeat domain 17 (ANKRD17), transcript variant 1, mRNA [NM_032217]
CTNNBIP1	up	1,180057	Homo sapiens catenin, beta interacting protein 1 (CTNNBIP1), transcript variant 1, mRNA [NM_020248]
PJA2	up	1,177321	Homo sapiens praja ring finger 2 (PJA2), mRNA [NM_014819]
AP3M1	up	1,176621	Homo sapiens adaptor-related protein complex 3, mu 1 subunit (AP3M1), transcript variant 1, mRNA [NM_207012]
HOXD1	up	1,175642	Homo sapiens homeobox D1 (HOXD1), mRNA [NM_024501]
ANKLE2	up	1,175134	Homo sapiens ankyrin repeat and LEM domain containing 2 (ANKLE2), mRNA [NM_015114]
SNAPC2	up	1,174336	Homo sapiens small nuclear RNA activating complex, polypeptide 2, 45kDa (SNAPC2), transcript variant 1, mRNA [NM_003083]
PRR20B	up	1,174128	Homo sapiens proline rich 20B (PRR20B), mRNA [NM_001130404]
C18orf21	up	1,173157	Homo sapiens chromosome 18 open reading frame 21 (C18orf21), transcript variant 1, mRNA [NM_031446]
AGTPBP1	up	1,172821	Homo sapiens ATP/GTP binding protein 1 (AGTPBP1), mRNA [NM_015239]
TMEM143	up	1,172175	Homo sapiens transmembrane protein 143 (TMEM143), mRNA [NM_018273]
ARPC5L	up	1,169371	Homo sapiens actin related protein 2/3 complex, subunit 5-like (ARPC5L), mRNA [NM_030978]
GNPAT	up	1,168098	Homo sapiens glyceronephosphate O-acyltransferase (GNPAT), mRNA [NM_014236]
MLX	up	1,167331	Homo sapiens MAX-like protein X (MLX), transcript variant 3, mRNA [NM_170607]
ZNF24	up	1,161973	Homo sapiens zinc finger protein 24 (ZNF24), mRNA [NM_006965]
LTV1	up	1,161803	Homo sapiens LTV1 homolog (S, cerevisiae) (LTV1), mRNA [NM_032860]
ATP5I	up	1,161679	Homo sapiens ATP synthase, H+ transporting, mitochondrial Fo complex, subunit E (ATP5I), nuclear gene encoding mitochondrial protein, transcript variant 1, mRNA [NM_007100]
USP19	up	1,161588	Homo sapiens ubiquitin specific peptidase 19 (USP19), transcript variant 2, mRNA [NM_001199161]

MDH2	up	1,159487	Homo sapiens malate dehydrogenase 2, NAD (mitochondrial) (MDH2), nuclear gene encoding mitochondrial protein, mRNA [NM_005918]
OTUD6B	up	1,156664	Homo sapiens OTU domain containing 6B (OTUD6B), mRNA [NM_016023]
FLJ43681	up	1,150713	Homo sapiens ribosomal protein L23a pseudogene (FLJ43681), non-coding RNA [NR_029406]
ZMYND8	up	1,150389	Homo sapiens zinc finger, MYND-type containing 8 (ZMYND8), transcript variant 1, mRNA [NM_183047]
UBE2D3	up	1,148739	Homo sapiens ubiquitin-conjugating enzyme E2D 3 (UBE2D3), transcript variant 2, mRNA [NM_181886]
RAB21	up	1,148701	Homo sapiens RAB21, member RAS oncogene family (RAB21), mRNA [NM_014999]
SS18L2	up	1,146444	Homo sapiens synovial sarcoma translocation gene on chromosome 18-like 2 (SS18L2), mRNA [NM_016305]
GNG5	up	1,145961	Homo sapiens guanine nucleotide binding protein (G protein), gamma 5 (GNG5), mRNA [NM_005274]
KPNB1	up	1,144346	Homo sapiens karyopherin (importin) beta 1 (KPNB1), mRNA [NM_002265]
CAPZA2	up	1,144067	Homo sapiens capping protein (actin filament) muscle Z-line, alpha 2 (CAPZA2), mRNA [NM_006136]
CD2AP	up	1,143535	Homo sapiens CD2-associated protein (CD2AP), mRNA [NM_012120]
KCNQ2	up	1,143376	potassium voltage-gated channel, KQT-like subfamily, member 2 [Source:HGNC Symbol;Acc:6296] [ENST00000370222]
RPS3	up	1,141876	Homo sapiens ribosomal protein S3 (RPS3), mRNA [NM_001005]
PAM16	up	1,139422	Homo sapiens presequence translocase-associated motor 16 homolog (S, cerevisiae) (PAM16), nuclear gene encoding mitochondrial protein, mRNA [NM_016069]
SCAF11	up	1,136975	Homo sapiens SR-related CTD-associated factor 11 (SCAF11), mRNA [NM_004719]
PPP2R5C	up	1,136274	Homo sapiens protein phosphatase 2, regulatory subunit B', gamma (PPP2R5C), transcript variant 3, mRNA [NM_178587]
ZC3H15	up	1,136194	Homo sapiens zinc finger CCCH-type containing 15 (ZC3H15), mRNA [NM_018471]
GMPR2	up	1,134937	Homo sapiens guanosine monophosphate reductase 2 (GMPR2), transcript variant 2, mRNA [NM_001002000]
BCKDHA	up	1,133794	Homo sapiens branched chain keto acid dehydrogenase E1, alpha polypeptide (BCKDHA), nuclear gene encoding mitochondrial protein, transcript variant 1, mRNA [NM_000709]
EIF2D	up	1,13363	Homo sapiens eukaryotic translation initiation factor 2D (EIF2D), transcript variant 1, mRNA [NM_006893]
SRP68	up	1,12967	Homo sapiens signal recognition particle 68kDa (SRP68), mRNA [NM_014230]
RBM12	up	1,127962	Homo sapiens RNA binding motif protein 12 (RBM12), transcript variant 1, mRNA [NM_006047]
FUNDC1	up	1,118451	Homo sapiens FUN14 domain containing 1 (FUNDC1), mRNA [NM_173794]

STAM2	up	1,116323	Homo sapiens signal transducing adaptor molecule (SH3 domain and ITAM motif) 2 (STAM2), mRNA [NM_005843]
UGP2	up	1,116188	Homo sapiens UDP-glucose pyrophosphorylase 2 (UGP2), transcript variant 1, mRNA [NM_006759]
RPS9	up	1,116171	Homo sapiens ribosomal protein S9 (RPS9), mRNA [NM_001013]
ZNHIT1	up	1,098932	Homo sapiens zinc finger, HIT-type containing 1 (ZNHIT1), mRNA [NM_006349]
CLPTM1L	up	1,097999	Homo sapiens CLPTM1-like (CLPTM1L), mRNA [NM_030782]
COPS5	up	1,070181	Homo sapiens COP9 constitutive photomorphogenic homolog subunit 5 (Arabidopsis) (COPS5), mRNA [NM_006837]
ANAPC4	up	1,068966	Homo sapiens anaphase promoting complex subunit 4 (ANAPC4), mRNA [NM_013367]
TMEM199	up	1,064801	Homo sapiens transmembrane protein 199 (TMEM199), mRNA [NM_152464]
CCDC167	down	-1,06764	Homo sapiens coiled-coil domain containing 167 (CCDC167), mRNA [NM_138493]
EZR	down	-1,07284	Homo sapiens ezrin (EZR), transcript variant 1, mRNA [NM_003379]
POLM	down	-1,08723	Homo sapiens polymerase (DNA directed), mu (POLM), mRNA [NM_013284]
SUMO2	down	-1,09514	Homo sapiens SMT3 suppressor of mif two 3 homolog 2 (S, cerevisiae) (SUMO2), transcript variant 1, mRNA [NM_006937]
PDCD7	down	-1,09968	Homo sapiens programmed cell death 7 (PDCD7), mRNA [NM_005707]
RABGAP1	down	-1,10252	Homo sapiens RAB GTPase activating protein 1 (RABGAP1), mRNA [NM_012197]
ING5	down	-1,10305	Homo sapiens inhibitor of growth family, member 5 (ING5), mRNA [NM_032329]
GSTK1	down	-1,1166	Homo sapiens glutathione S-transferase kappa 1 (GSTK1), nuclear gene encoding mitochondrial protein, transcript variant 2, mRNA [NM_001143679]
PANK4	down	-1,11782	Homo sapiens pantothenate kinase 4 (PANK4), mRNA [NM_018216]
ARFRP1	down	-1,11782	Homo sapiens ADP-ribosylation factor related protein 1 (ARFRP1), transcript variant 2, mRNA [NM_001134758]
SCRIB	down	-1,12068	Homo sapiens scribbled homolog (Drosophila) (SCRIB), transcript variant 1, mRNA [NM_182706]
TOP2B	down	-1,12434	Homo sapiens topoisomerase (DNA) II beta 180kDa (TOP2B), mRNA [NM_001068]
ARID5A	down	-1,12735	Homo sapiens AT rich interactive domain 5A (MRF1-like) (ARID5A), mRNA [NM_212481]
PPP1CA	down	-1,12853	Homo sapiens protein phosphatase 1, catalytic subunit, alpha isozyme (PPP1CA), transcript variant 3, mRNA [NM_001008709]
EIF3B	down	-1,13611	Homo sapiens eukaryotic translation initiation factor 3, subunit B (EIF3B), transcript variant 2, mRNA [NM_001037283]
ND5	down	-1,13998	mitochondrially encoded NADH dehydrogenase 5 [Source:HGNC Symbol;Acc:7461] [ENST00000361567]

DDX49	down	-1,14244	Homo sapiens DEAD (Asp-Glu-Ala-Asp) box polypeptide 49 (DDX49), transcript variant 1, mRNA [NM_019070]
MYH14	down	-1,14363	Homo sapiens myosin, heavy chain 14, non-muscle (MYH14), transcript variant 1, mRNA [NM_001077186]
IPO13	down	-1,14402	Homo sapiens importin 13 (IPO13), mRNA [NM_014652]
MRPS2	down	-1,15128	Homo sapiens mitochondrial ribosomal protein S2 (MRPS2), nuclear gene encoding mitochondrial protein, mRNA [NM_016034]
ALG1L	down	-1,15702	Homo sapiens asparagine-linked glycosylation 1-like (ALG1L), transcript variant 2, mRNA [NM_001015050]
ESYT1	down	-1,15826	Homo sapiens extended synaptotagmin-like protein 1 (ESYT1), transcript variant 2, mRNA [NM_015292]
CCDC167	down	-1,15832	Homo sapiens coiled-coil domain containing 167 (CCDC167), mRNA [NM_138493]
NCAPH2	down	-1,16072	Homo sapiens non-SMC condensin II complex, subunit H2 (NCAPH2), transcript variant 3, mRNA [NM_001185011]
INTS1	down	-1,16124	Homo sapiens integrator complex subunit 1 (INTS1), mRNA [NM_001080453]
LOC375190	down	-1,16203	Homo sapiens hypothetical protein LOC375190 (LOC375190), mRNA [NM_001145710]
SDR42E1	down	-1,16734	Homo sapiens short chain dehydrogenase/reductase family 42E, member 1 (SDR42E1), mRNA [NM_145168]
SUGP1	down	-1,16859	Homo sapiens SURP and G patch domain containing 1 (SUGP1), mRNA [NM_172231]
SEPHS1	down	-1,16864	Homo sapiens selenophosphate synthetase 1 (SEPHS1), transcript variant 1, mRNA [NM_012247]
MED24	down	-1,16887	Homo sapiens mediator complex subunit 24 (MED24), transcript variant 1, mRNA [NM_014815]
PSMC1	down	-1,17052	Homo sapiens proteasome (prosome, macropain) 26S subunit, ATPase, 1 (PSMC1), mRNA [NM_002802]
GUSBP1	down	-1,17133	Homo sapiens glucuronidase, beta pseudogene 1 (GUSBP1), transcript variant 3, non-coding RNA [NR_027028]
PEMT	down	-1,17268	Homo sapiens phosphatidylethanolamine N-methyltransferase (PEMT), nuclear gene encoding mitochondrial protein, transcript variant 2, mRNA [NM_007169]
MICA	down	-1,17282	Homo sapiens MHC class I polypeptide-related sequence A (MICA), transcript variant 1 (allele MICA*001), mRNA [NM_000247]
PCCA	down	-1,17351	Homo sapiens propionyl CoA carboxylase, alpha polypeptide (PCCA), nuclear gene encoding mitochondrial protein, transcript variant 1, mRNA [NM_000282]
DDHD2	down	-1,17405	Homo sapiens DDHD domain containing 2 (DDHD2), transcript variant 1, mRNA [NM_015214]
CCNG1	down	-1,17477	Homo sapiens cyclin G1 (CCNG1), transcript variant 1, mRNA [NM_004060]
ERO1L	down	-1,17533	Homo sapiens ERO1-like (S, cerevisiae) (ERO1L), mRNA [NM_014584]

SH3KBP1	down	-1,17602	Homo sapiens SH3-domain kinase binding protein 1 (SH3KBP1), transcript variant 1, mRNA [NM_031892]
MAK16	down	-1,1761	Homo sapiens MAK16 homolog (S, cerevisiae) (MAK16), mRNA [NM_032509]
DTL	down	-1,17688	Homo sapiens denticleless homolog (Drosophila) (DTL), mRNA [NM_016448]
PMVK	down	-1,17765	Homo sapiens phosphomevalonate kinase (PMVK), mRNA [NM_006556]
CHKA	down	-1,17797	Homo sapiens choline kinase alpha (CHKA), transcript variant 1, mRNA [NM_001277]
PHPT1	down	-1,17892	Homo sapiens phosphohistidine phosphatase 1 (PHPT1), transcript variant 3, mRNA [NM_014172]
AGPAT3	down	-1,18071	Homo sapiens 1-acylglycerol-3-phosphate O-acyltransferase 3 (AGPAT3), transcript variant 1, mRNA [NM_020132]
LAP3	down	-1,18497	Homo sapiens leucine aminopeptidase 3 (LAP3), mRNA [NM_015907]
TSSC4	down	-1,18498	Homo sapiens tumor suppressing subtransferable candidate 4 (TSSC4), mRNA [NM_005706]
TWF2	down	-1,18541	Homo sapiens twinfilin, actin-binding protein, homolog 2 (Drosophila) (TWF2), mRNA [NM_007284]
TBP	down	-1,18569	Homo sapiens TATA box binding protein (TBP), transcript variant 1, mRNA [NM_003194]
RECQL4	down	-1,18579	Homo sapiens RecQ protein-like 4 (RECQL4), mRNA [NM_004260]
ATAD3B	down	-1,18747	Homo sapiens ATPase family, AAA domain containing 3B (ATAD3B), nuclear gene encoding mitochondrial protein, mRNA [NM_031921]
SRC	down	-1,18866	Homo sapiens v-src sarcoma (Schmidt-Ruppin A-2) viral oncogene homolog (avian) (SRC), transcript variant 1, mRNA [NM_005417]
SLC26A6	down	-1,18886	Homo sapiens solute carrier family 26, member 6 (SLC26A6), transcript variant 4, mRNA [NM_001040454]
ALKBH3	down	-1,1895	Homo sapiens alkB, alkylation repair homolog 3 (E, coli) (ALKBH3), mRNA [NM_139178]
C16orf80	down	-1,18999	Homo sapiens chromosome 16 open reading frame 80 (C16orf80), mRNA [NM_013242]
KSR1	down	-1,19038	Homo sapiens kinase suppressor of ras 1 (KSR1), mRNA [NM_014238]
PPP2R5E	down	-1,19048	Homo sapiens protein phosphatase 2, regulatory subunit B', epsilon isoform (PPP2R5E), mRNA [NM_006246]
CRBN	down	-1,19107	Homo sapiens cereblon (CRBN), transcript variant 1, mRNA [NM_016302]
KRI1	down	-1,19141	Homo sapiens KRI1 homolog (S, cerevisiae) (KRI1), mRNA [NM_023008]
C20orf43	down	-1,19222	Homo sapiens chromosome 20 open reading frame 43 (C20orf43), mRNA [NM_016407]
HAUS2	down	-1,19237	Homo sapiens HAUS augmin-like complex, subunit 2 (HAUS2), transcript variant 1, mRNA [NM_018097]
CCDC25	down	-1,19296	Homo sapiens coiled-coil domain containing 25 (CCDC25), mRNA [NM_018246]
PPIA	down	-1,19347	Homo sapiens peptidylprolyl isomerase A (cyclophilin A) (PPIA), mRNA [NM_021130]
ZBTB48	down	-1,19412	Homo sapiens zinc finger and BTB domain containing 48 (ZBTB48), mRNA [NM_005341]

SMYD5	down	-1,19439	Homo sapiens SMYD family member 5 (SMYD5), mRNA [NM_006062]
FAM195A	down	-1,19554	Homo sapiens family with sequence similarity 195, member A (FAM195A), mRNA [NM_138418]
RABEP1	down	-1,1956	Homo sapiens rabaptin, RAB GTPase binding effector protein 1 (RABEP1), transcript variant 1, mRNA [NM_004703]
ILF3	down	-1,19693	Homo sapiens interleukin enhancer binding factor 3, 90kDa (ILF3), transcript variant 1, mRNA [NM_012218]
PANX1	down	-1,19802	Homo sapiens pannexin 1 (PANX1), mRNA [NM_015368]
GPS1	down	-1,20212	Homo sapiens G protein pathway suppressor 1 (GPS1), transcript variant 1, mRNA [NM_212492]
PSMC1	down	-1,20426	Homo sapiens proteasome (prosome, macropain) 26S subunit, ATPase, 1 (PSMC1), mRNA [NM_002802]
RBKS	down	-1,2058	Homo sapiens ribokinase (RBKS), mRNA [NM_022128]
APOBEC3C	down	-1,20788	Homo sapiens apolipoprotein B mRNA editing enzyme, catalytic polypeptide-like 3C (APOBEC3C), mRNA [NM_014508]
LOC100132273	down	-1,20794	Homo sapiens hypothetical LOC100132273 (LOC100132273), non-coding RNA [NR_034118]
MAP3K3	down	-1,20805	Homo sapiens mitogen-activated protein kinase kinase kinase 3 (MAP3K3), transcript variant 1, mRNA [NM_203351]
BCAP29	down	-1,20958	Homo sapiens B-cell receptor-associated protein 29 (BCAP29), transcript variant 2, mRNA [NM_018844]
MAP7D1	down	-1,20959	Homo sapiens MAP7 domain containing 1 (MAP7D1), mRNA [NM_018067]
CLSTN3	down	-1,2098	Homo sapiens calsyntenin 3 (CLSTN3), mRNA [NM_014718]
ITPR3	down	-1,21057	Homo sapiens inositol 1,4,5-trisphosphate receptor, type 3 (ITPR3), mRNA [NM_002224]
SAP30BP	down	-1,21183	Homo sapiens SAP30 binding protein (SAP30BP), mRNA [NM_013260]
PKN1	down	-1,21215	Homo sapiens protein kinase N1 (PKN1), transcript variant 2, mRNA [NM_002741]
AGPAT6	down	-1,21715	Homo sapiens 1-acylglycerol-3-phosphate O-acyltransferase 6 (lysophosphatidic acid acyltransferase, zeta) (AGPAT6), mRNA [NM_178819]
SBF1	down	-1,21792	Homo sapiens SET binding factor 1 (SBF1), mRNA [NM_002972]
FAM35A	down	-1,21806	Homo sapiens family with sequence similarity 35, member A (FAM35A), mRNA [NM_019054]
UBE2J2	down	-1,22223	Homo sapiens ubiquitin-conjugating enzyme E2, J2 (UBE2J2), transcript variant 3, mRNA [NM_194458]
DRG2	down	-1,22474	Homo sapiens developmentally regulated GTP binding protein 2 (DRG2), mRNA [NM_001388]
ELANE	down	-1,2261	Homo sapiens elastase, neutrophil expressed (ELANE), mRNA [NM_001972]
SPPL3	down	-1,22926	Homo sapiens signal peptide peptidase-like 3 (SPPL3), mRNA [NM_139015]
ZNF587	down	-1,2294	Homo sapiens zinc finger protein 587 (ZNF587), transcript variant 1, mRNA [NM_032828]

ANKAR	down	-1,23012	Homo sapiens ankyrin and armadillo repeat containing (ANKAR), mRNA [NM_144708]
EIF6	down	-1,23025	Homo sapiens eukaryotic translation initiation factor 6 (EIF6), transcript variant 2, mRNA [NM_181468]
SLC2A11	down	-1,23084	Homo sapiens solute carrier family 2 (facilitated glucose transporter), member 11 (SLC2A11), transcript variant 1, mRNA [NM_030807]
NARFL	down	-1,23114	Homo sapiens nuclear prelamin A recognition factor-like (NARFL), mRNA [NM_022493]
B3GNT7	down	-1,23243	Homo sapiens UDP-GlcNAc:betaGal beta-1,3-N-acetylglucosaminyltransferase 7 (B3GNT7), mRNA [NM_145236]
DDX21	down	-1,23282	Homo sapiens DEAD (Asp-Glu-Ala-Asp) box polypeptide 21 (DDX21), mRNA [NM_004728]
LOC100130285	down	-1,23343	PREDICTED: Homo sapiens hypothetical LOC100130285 (LOC100130285), miscRNA [XR_109235]
TAF15	down	-1,2339	Homo sapiens TAF15 RNA polymerase II, TATA box binding protein (TBP)-associated factor, 68kDa (TAF15), transcript variant 1, mRNA [NM_139215]
ING4	down	-1,23403	Homo sapiens inhibitor of growth family, member 4 (ING4), transcript variant 1, mRNA [NM_016162]
FBXW5	down	-1,23442	Homo sapiens F-box and WD repeat domain containing 5 (FBXW5), mRNA [NM_018998]
MRPS26	down	-1,23495	Homo sapiens mitochondrial ribosomal protein S26 (MRPS26), nuclear gene encoding mitochondrial protein, mRNA [NM_030811]
RTTN	down	-1,23523	Homo sapiens rotatin (RTTN), mRNA [NM_173630]
MTERFD3	down	-1,23613	Homo sapiens MTERF domain containing 3 (MTERFD3), transcript variant 1, mRNA [NM_001033050]
FANCM	down	-1,23665	Homo sapiens Fanconi anemia, complementation group M (FANCM), mRNA [NM_020937]
ZNF193	down	-1,23666	Homo sapiens zinc finger protein 193 (ZNF193), transcript variant 2, mRNA [NM_006299]
SDHA	down	-1,23668	Homo sapiens succinate dehydrogenase complex, subunit A, flavoprotein (Fp) (SDHA), nuclear gene encoding mitochondrial protein, mRNA [NM_004168]
ILK	down	-1,23738	Homo sapiens integrin-linked kinase (ILK), transcript variant 3, mRNA [NM_001014795]
ATAD3A	down	-1,23768	Homo sapiens ATPase family, AAA domain containing 3A (ATAD3A), nuclear gene encoding mitochondrial protein, transcript variant 1, mRNA [NM_018188]
GPATCH4	down	-1,2393	Homo sapiens G patch domain containing 4 (GPATCH4), transcript variant 2, mRNA [NM_182679]
ZSWIM3	down	-1,24272	Homo sapiens zinc finger, SWIM-type containing 3 (ZSWIM3), transcript variant 1, mRNA [NM_080752]
SHARPIN	down	-1,24355	Homo sapiens SHANK-associated RH domain interactor (SHARPIN), transcript variant 1, mRNA [NM_030974]
ILVBL	down	-1,24452	Homo sapiens ilvB (bacterial acetolactate synthase)-like (ILVBL), mRNA [NM_006844]
MPRIP	down	-1,24621	Homo sapiens myosin phosphatase Rho interacting protein (MPRIP), transcript variant 1, mRNA [NM_015134]
UROS	down	-1,24633	Homo sapiens uroporphyrinogen III synthase (UROS), mRNA [NM_000375]

LRP8	down	-1,24664	Homo sapiens low density lipoprotein receptor-related protein 8, apolipoprotein e receptor (LRP8), transcript variant 2, mRNA [NM_033300]
TMEM219	down	-1,24668	Homo sapiens transmembrane protein 219 (TMEM219), transcript variant 2, mRNA [NM_194280]
NARS	down	-1,24672	Homo sapiens asparaginyl-tRNA synthetase (NARS), mRNA [NM_004539]
PYGB	down	-1,24813	Homo sapiens phosphorylase, glycogen; brain (PYGB), mRNA [NM_002862]
PLEKHJ1	down	-1,2485	Homo sapiens pleckstrin homology domain containing, family J member 1 (PLEKHJ1), mRNA [NM_018049]
TUBB2C	down	-1,2492	Homo sapiens tubulin, beta 2C (TUBB2C), mRNA [NM_006088]
FGF9	down	-1,24933	Homo sapiens fibroblast growth factor 9 (glia-activating factor) (FGF9), mRNA [NM_002010]
RAB35	down	-1,25247	Homo sapiens RAB35, member RAS oncogene family (RAB35), transcript variant 1, mRNA [NM_006861]
ZBTB24	down	-1,25252	Homo sapiens zinc finger and BTB domain containing 24 (ZBTB24), transcript variant 1, mRNA [NM_014797]
SIAH1	down	-1,25665	Homo sapiens seven in absentia homolog 1 (Drosophila) (SIAH1), transcript variant 1, mRNA [NM_003031]
GRB10	down	-1,25669	Homo sapiens growth factor receptor-bound protein 10 (GRB10), transcript variant 4, mRNA [NM_001001555]
FAM22F	down	-1,25722	Homo sapiens family with sequence similarity 22, member F (FAM22F), mRNA [NM_017561]
EIF4E	down	-1,25727	Homo sapiens eukaryotic translation initiation factor 4E (EIF4E), transcript variant 1, mRNA [NM_001968]
CC2D1A	down	-1,2596	Homo sapiens coiled-coil and C2 domain containing 1A (CC2D1A), mRNA [NM_017721]
AKAP10	down	-1,25961	Homo sapiens A kinase (PRKA) anchor protein 10 (AKAP10), nuclear gene encoding mitochondrial protein, mRNA [NM_007202]
ZNF341	down	-1,26118	Homo sapiens zinc finger protein 341 (ZNF341), mRNA [NM_032819]
C12orf41	down	-1,26148	Homo sapiens chromosome 12 open reading frame 41 (C12orf41), mRNA [NM_017822]
ATG4D	down	-1,26174	Homo sapiens ATG4 autophagy related 4 homolog D (S, cerevisiae) (ATG4D), mRNA [NM_032885]
NOSIP	down	-1,26318	Homo sapiens nitric oxide synthase interacting protein (NOSIP), mRNA [NM_015953]
FAM135A	down	-1,2638	Homo sapiens family with sequence similarity 135, member A (FAM135A), transcript variant 2, mRNA [NM_020819]
STX16	down	-1,26542	Homo sapiens syntaxin 16 (STX16), transcript variant 1, mRNA [NM_001001433]
INPPL1	down	-1,26653	Homo sapiens inositol polyphosphate phosphatase-like 1 (INPPL1), mRNA [NM_001567]
PTOV1	down	-1,26696	Homo sapiens prostate tumor overexpressed 1 (PTOV1), mRNA [NM_017432]
ZNF691	down	-1,2675	Homo sapiens zinc finger protein 691 (ZNF691), transcript variant 2, mRNA [NM_015911]

KCTD1	down	-1,26844	Homo sapiens potassium channel tetramerisation domain containing 1 (KCTD1), transcript variant 2, mRNA [NM_198991]
NLRX1	down	-1,26892	Homo sapiens NLR family member X1 (NLRX1), transcript variant 2, mRNA [NM_170722]
GTSE1	down	-1,26941	Homo sapiens G-2 and S-phase expressed 1 (GTSE1), mRNA [NM_016426]
ADCK2	down	-1,27038	Homo sapiens aarF domain containing kinase 2 (ADCK2), mRNA [NM_052853]
MCRS1	down	-1,2713	Homo sapiens microspherule protein 1 (MCRS1), transcript variant 1, mRNA [NM_006337]
C3orf26	down	-1,27146	Homo sapiens chromosome 3 open reading frame 26 (C3orf26), transcript variant 1, mRNA [NM_032359]
MAFF	down	-1,27222	Homo sapiens v-maf musculoaponeurotic fibrosarcoma oncogene homolog F (avian) (MAFF), transcript variant 1, mRNA [NM_012323]
SRD5A1	down	-1,2725	Homo sapiens steroid-5-alpha-reductase, alpha polypeptide 1 (3-oxo-5 alpha-steroid delta 4-dehydrogenase alpha 1) (SRD5A1), mRNA [NM_001047]
UROS	down	-1,27266	Homo sapiens uroporphyrinogen III synthase (UROS), mRNA [NM_000375]
TMCO4	down	-1,27281	Homo sapiens transmembrane and coiled-coil domains 4 (TMCO4), mRNA [NM_181719]
ERI1	down	-1,27569	Homo sapiens exoribonuclease 1 (ERI1), mRNA [NM_153332]
PBX4	down	-1,27606	Homo sapiens pre-B-cell leukemia homeobox 4 (PBX4), transcript variant 1, mRNA [NM_025245]
CYTH2	down	-1,2761	Homo sapiens cytohesin 2 (CYTH2), transcript variant 2, mRNA [NM_004228]
SDHA	down	-1,2761	Homo sapiens succinate dehydrogenase complex, subunit A, flavoprotein (Fp) (SDHA), nuclear gene encoding mitochondrial protein, mRNA [NM_004168]
ITPRIP	down	-1,27792	Homo sapiens inositol 1,4,5-trisphosphate receptor interacting protein (ITPRIP), mRNA [NM_033397]
FIBP	down	-1,27925	Homo sapiens fibroblast growth factor (acidic) intracellular binding protein (FIBP), transcript variant 2, mRNA [NM_004214]
SHARPIN	down	-1,27963	Homo sapiens SHANK-associated RH domain interactor (SHARPIN), transcript variant 1, mRNA [NM_030974]
SARS	down	-1,28028	Homo sapiens seryl-tRNA synthetase (SARS), transcript variant 1, mRNA [NM_006513]
ARID5A	down	-1,28411	Homo sapiens AT rich interactive domain 5A (MRF1-like) (ARID5A), mRNA [NM_212481]
MAN1B1	down	-1,28574	Homo sapiens mannosidase, alpha, class 1B, member 1 (MAN1B1), mRNA [NM_016219]
VAPB	down	-1,2861	Homo sapiens VAMP (vesicle-associated membrane protein)-associated protein B and C (VAPB), transcript variant 1, mRNA [NM_004738]
NFKBIE	down	-1,28815	Homo sapiens nuclear factor of kappa light polypeptide gene enhancer in B-cells inhibitor, epsilon (NFKBIE), mRNA [NM_004556]
DNAJC11	down	-1,28945	Homo sapiens DnaJ (Hsp40) homolog, subfamily C, member 11 (DNAJC11), mRNA [NM_018198]
ADCK5	down	-1,29011	Homo sapiens aarF domain containing kinase 5 (ADCK5), mRNA [NM_174922]

NDUFAF1	down	-1,29039	Homo sapiens NADH dehydrogenase (ubiquinone) 1 alpha subcomplex, assembly factor 1 (NDUFAF1), mRNA [NM_016013]
ASPSCR1	down	-1,29081	Homo sapiens alveolar soft part sarcoma chromosome region, candidate 1 (ASPSCR1), mRNA [NM_024083]
DCAF15	down	-1,29168	Homo sapiens DDB1 and CUL4 associated factor 15 (DCAF15), mRNA [NM_138353]
KLHL2	down	-1,2918	Homo sapiens kelch-like 2, Mayven (Drosophila) (KLHL2), transcript variant 1, mRNA [NM_007246]
CRIPT	down	-1,29221	Homo sapiens cysteine-rich PDZ-binding protein (CRIPT), mRNA [NM_014171]
SH3GLB2	down	-1,29302	Homo sapiens SH3-domain GRB2-like endophilin B2 (SH3GLB2), mRNA [NM_020145]
SH3BP5L	down	-1,29312	Homo sapiens SH3-binding domain protein 5-like (SH3BP5L), mRNA [NM_030645]
CLCN7	down	-1,29348	Homo sapiens chloride channel 7 (CLCN7), transcript variant 1, mRNA [NM_001287]
ENTPD6	down	-1,29361	Homo sapiens ectonucleoside triphosphate diphosphohydrolase 6 (putative) (ENTPD6), transcript variant 1, mRNA [NM_001247]
LRFN4	down	-1,29391	Homo sapiens leucine rich repeat and fibronectin type III domain containing 4 (LRFN4), mRNA [NM_024036]
FAM131A	down	-1,29418	Homo sapiens family with sequence similarity 131, member A (FAM131A), transcript variant 1, mRNA [NM_144635]
URGCP	down	-1,29431	Homo sapiens upregulator of cell proliferation (URGCP), nuclear gene encoding mitochondrial protein, transcript variant 2, mRNA [NM_001077664]
ZNF687	down	-1,29559	Homo sapiens zinc finger protein 687 (ZNF687), mRNA [NM_020832]
ZNF512B	down	-1,29976	Homo sapiens zinc finger protein 512B (ZNF512B), mRNA [NM_020713]
UBE2I	down	-1,3011	Homo sapiens ubiquitin-conjugating enzyme E2I (UBC9 homolog, yeast) [Source:HGNC Symbol;Acc:12485] [ENST00000402301]
C3	down	-1,30133	Homo sapiens complement component 3 (C3), mRNA [NM_000064]
LENG1	down	-1,30196	Homo sapiens leukocyte receptor cluster (LRC) member 1 (LENG1), mRNA [NM_024316]
FBXO18	down	-1,30428	Homo sapiens F-box protein, helicase, 18 (FBXO18), transcript variant 2, mRNA [NM_178150]
ZNF19	down	-1,30436	Homo sapiens zinc finger protein 19 (ZNF19), mRNA [NM_006961]
TCOF1	down	-1,30481	Homo sapiens Treacher Collins-Franceschetti syndrome 1 (TCOF1), transcript variant 3, mRNA [NM_001008657]
ARHGAP30	down	-1,30628	Homo sapiens Rho GTPase activating protein 30 (ARHGAP30), transcript variant 2, mRNA [NM_181720]
GPX4	down	-1,30769	Homo sapiens glutathione peroxidase 4 (phospholipid hydroperoxidase) (GPX4), transcript variant 3, mRNA [NM_001039848]
PPM1M	down	-1,30999	Homo sapiens protein phosphatase, Mg2+/Mn2+ dependent, 1M (PPM1M), transcript variant 1, mRNA [NM_144641]

LRRC8D	down	-1,31001	Homo sapiens leucine rich repeat containing 8 family, member D (LRRC8D), transcript variant 2, mRNA [NM_018103]
GIT1	down	-1,31146	Homo sapiens G protein-coupled receptor kinase interacting ArfGAP 1 (GIT1), transcript variant 2, mRNA [NM_014030]
GAS8	down	-1,31181	Homo sapiens growth arrest-specific 8 (GAS8), transcript variant 1, mRNA [NM_001481]
RECQL5	down	-1,31225	Homo sapiens RecQ protein-like 5 (RECQL5), transcript variant 1, mRNA [NM_004259]
RAB1B	down	-1,31229	Homo sapiens RAB1B, member RAS oncogene family (RAB1B), mRNA [NM_030981]
ZNF136	down	-1,31345	Homo sapiens zinc finger protein 136 (ZNF136), mRNA [NM_003437]
HNRNPD	down	-1,31581	Homo sapiens heterogeneous nuclear ribonucleoprotein D (AU-rich element RNA binding protein 1, 37kDa) (HNRNPD), transcript variant 1, mRNA [NM_031370]
CD81	down	-1,31619	Homo sapiens CD81 molecule (CD81), mRNA [NM_004356]
CEP95	down	-1,32082	Homo sapiens centrosomal protein 95kDa (CEP95), mRNA [NM_138363]
KLHL23	down	-1,32132	Homo sapiens kelch-like 23 (Drosophila) (KLHL23), mRNA [NM_144711]
LOC389906	down	-1,32149	Homo sapiens hypothetical LOC389906 (LOC389906), non-coding RNA [NR_034031]
MAST2	down	-1,32217	Homo sapiens microtubule associated serine/threonine kinase 2 (MAST2), mRNA [NM_015112]
LYPLA2	down	-1,32263	Homo sapiens lysophospholipase II (LYPLA2), mRNA [NM_007260]
ZNF3	down	-1,32266	Homo sapiens zinc finger protein 3 (ZNF3), transcript variant 2, mRNA [NM_032924]
TRMT61A	down	-1,32325	Homo sapiens tRNA methyltransferase 61 homolog A (S, cerevisiae) (TRMT61A), mRNA [NM_152307]
TRAIP	down	-1,32374	Homo sapiens TRAF interacting protein (TRAIP), mRNA [NM_005879]
RFX5	down	-1,32377	Homo sapiens regulatory factor X, 5 (influences HLA class II expression) (RFX5), transcript variant 1, mRNA [NM_000449]
SUV39H1	down	-1,32516	Homo sapiens suppressor of variegation 3-9 homolog 1 (Drosophila) (SUV39H1), mRNA [NM_003173]
TTC38	down	-1,32608	Homo sapiens tetratricopeptide repeat domain 38 (TTC38), mRNA [NM_017931]
STXBP2	down	-1,3262	Homo sapiens syntaxin binding protein 2 (STXBP2), transcript variant 1, mRNA [NM_006949]
PLEKHA8	down	-1,32829	Homo sapiens pleckstrin homology domain containing, family A (phosphoinositide binding specific) member 8 (PLEKHA8), transcript variant 3, mRNA [NM_032639]
PPP2R5D	down	-1,33005	Homo sapiens protein phosphatase 2, regulatory subunit B', delta (PPP2R5D), transcript variant 2, mRNA [NM_180976]
PPIE	down	-1,33043	Homo sapiens peptidylprolyl isomerase E (cyclophilin E) (PPIE), transcript variant 2, mRNA [NM_203456]
DVL1	down	-1,3316	Homo sapiens dishevelled, dsh homolog 1 (Drosophila) (DVL1), mRNA [NM_004421]
DOT1L	down	-1,33221	Homo sapiens DOT1-like, histone H3 methyltransferase (S, cerevisiae) (DOT1L), mRNA [NM_032482]

RBM23	down	-1,33335	Homo sapiens RNA binding motif protein 23 (RBM23), transcript variant 1, mRNA [NM_001077351]
ZFP36L2	down	-1,33541	Homo sapiens zinc finger protein 36, C3H type-like 2 (ZFP36L2), mRNA [NM_006887]
FKBPL	down	-1,33807	Homo sapiens FK506 binding protein like (FKBPL), mRNA [NM_022110]
KIAA0415	down	-1,33822	Homo sapiens KIAA0415 (KIAA0415), mRNA [NM_014855]
HDAC10	down	-1,33869	Homo sapiens histone deacetylase 10 (HDAC10), transcript variant 1, mRNA [NM_032019]
UBE2D4	down	-1,33979	Homo sapiens ubiquitin-conjugating enzyme E2D 4 (putative) (UBE2D4), mRNA [NM_015983]
ALKBH3	down	-1,33985	Homo sapiens alkB, alkylation repair homolog 3 (E, coli) (ALKBH3), mRNA [NM_139178]
ANKS3	down	-1,34092	Homo sapiens ankyrin repeat and sterile alpha motif domain containing 3 (ANKS3), transcript variant 1, mRNA [NM_133450]
C12orf66	down	-1,34409	Homo sapiens chromosome 12 open reading frame 66 (C12orf66), mRNA [NM_152440]
CD81	down	-1,34768	Homo sapiens CD81 molecule (CD81), mRNA [NM_004356]
LOC729683	down	-1,34825	full-length cDNA clone CS0DL006YD08 of B cells (Ramos cell line) Cot 25-normalized of Homo sapiens (human), [CR594811]
KIAA0664	down	-1,34869	Homo sapiens KIAA0664 (KIAA0664), mRNA [NM_015229]
ICAM2	down	-1,3497	Homo sapiens intercellular adhesion molecule 2 (ICAM2), transcript variant 5, mRNA [NM_000873]
SESN2	down	-1,35041	Homo sapiens sestrin 2 (SESN2), mRNA [NM_031459]
CTAGE15P	down	-1,35084	Homo sapiens CTAGE family, member 15, pseudogene (CTAGE15P), mRNA [NM_001008747]
RALY	down	-1,35246	Homo sapiens RNA binding protein, autoantigenic (hnRNP-associated with lethal yellow homolog (mouse)) (RALY), transcript variant 1, mRNA [NM_016732]
UNC45A	down	-1,35355	Homo sapiens unc-45 homolog A (C, elegans) (UNC45A), transcript variant 3, mRNA [NM_001039675]
FOXM1	down	-1,35437	Homo sapiens forkhead box M1 (FOXM1), transcript variant 1, mRNA [NM_202002]
TFEB	down	-1,35548	Homo sapiens transcription factor EB (TFEB), transcript variant 1, mRNA [NM_007162]
TPD52L2	down	-1,35565	Homo sapiens tumor protein D52-like 2 (TPD52L2), transcript variant 1, mRNA [NM_199360]
C19orf25	down	-1,35715	Homo sapiens chromosome 19 open reading frame 25 (C19orf25), mRNA [NM_152482]
VAMP3	down	-1,35771	Homo sapiens vesicle-associated membrane protein 3 (cellubrevin) (VAMP3), mRNA [NM_004781]
NETO2	down	-1,35776	Homo sapiens neuropilin (NRP) and tolloid (TLL)-like 2 (NETO2), transcript variant 1, mRNA [NM_018092]
CDHR1	down	-1,35842	Homo sapiens cadherin-related family member 1 (CDHR1), transcript variant 1, mRNA [NM_033100]
ST8SIA4	down	-1,35856	Homo sapiens ST8 alpha-N-acetyl-neuraminide alpha-2,8-sialyltransferase 4 (ST8SIA4), transcript variant 1, mRNA [NM_005668]
LOC388692	down	-1,35972	Homo sapiens hypothetical LOC388692 (LOC388692), non-coding RNA [NR_027002]

MAP4K1	down	-1,35976	Homo sapiens mitogen-activated protein kinase kinase kinase kinase 1 (MAP4K1), transcript variant 1, mRNA [NM_001042600]
C19orf28	down	-1,36117	Homo sapiens chromosome 19 open reading frame 28 (C19orf28), transcript variant 2, mRNA [NM_001042680]
C7orf41	down	-1,36167	Homo sapiens chromosome 7 open reading frame 41 (C7orf41), mRNA [NM_152793]
DNM2	down	-1,36277	Homo sapiens dynamin 2 (DNM2), transcript variant 1, mRNA [NM_001005360]
FADD	down	-1,36339	Homo sapiens Fas (TNFRSF6)-associated via death domain (FADD), mRNA [NM_003824]
BACE2	down	-1,36475	Homo sapiens beta-site APP-cleaving enzyme 2 (BACE2), transcript variant a, mRNA [NM_012105]
C15orf17	down	-1,3661	Homo sapiens chromosome 15 open reading frame 17 (C15orf17), mRNA [NM_020447]
HSBP1	down	-1,36848	Homo sapiens heat shock factor binding protein 1 (HSBP1), mRNA [NM_001537]
MED25	down	-1,36873	Homo sapiens mediator complex subunit 25 (MED25), mRNA [NM_030973]
C11orf57	down	-1,37018	Homo sapiens chromosome 11 open reading frame 57 (C11orf57), transcript variant 1, mRNA [NM_018195]
STARD5	down	-1,371	Homo sapiens StAR-related lipid transfer (START) domain containing 5 (STARD5), mRNA [NM_181900]
KIF22	down	-1,37121	Homo sapiens kinesin family member 22 (KIF22), mRNA [NM_007317]
RBMY1B	down	-1,37149	Homo sapiens RNA binding motif protein, Y-linked, family 1, member B (RBMY1B), mRNA [NM_001006121]
SYS1	down	-1,37153	Homo sapiens SYS1 Golgi-localized integral membrane protein homolog (S, cerevisiae) (SYS1), transcript variant 3, mRNA [NM_001197129]
DNM1P46	down	-1,37206	Homo sapiens DNM1 pseudogene 46 (DNM1P46), non-coding RNA [NR_003260]
LOC100133050	down	-1,37416	Homo sapiens glucuronidase, beta pseudogene (LOC100133050), non-coding RNA [NR_027503]
CERS4	down	-1,37501	Homo sapiens ceramide synthase 4 (CERS4), mRNA [NM_024552]
ING3	down	-1,37511	Homo sapiens inhibitor of growth family, member 3 (ING3), transcript variant 3, mRNA [NM_198267]
ZNF137P	down	-1,37541	Homo sapiens zinc finger protein 137, pseudogene (ZNF137P), non-coding RNA [NR_023311]
SMG5	down	-1,37624	Homo sapiens smg-5 homolog, nonsense mediated mRNA decay factor (C, elegans) (SMG5), mRNA [NM_015327]
DHX37	down	-1,37647	Homo sapiens DEAH (Asp-Glu-Ala-His) box polypeptide 37 (DHX37), mRNA [NM_032656]
USP42	down	-1,37657	Homo sapiens ubiquitin specific peptidase 42 (USP42), mRNA [NM_032172]
C7orf50	down	-1,3767	Homo sapiens chromosome 7 open reading frame 50 (C7orf50), transcript variant 1, mRNA [NM_032350]
NAA16	down	-1,37926	Homo sapiens N(alpha)-acetyltransferase 16, NatA auxiliary subunit (NAA16), transcript variant 1, mRNA [NM_024561]

IRF3	down	-1,3806	Homo sapiens interferon regulatory factor 3 (IRF3), transcript variant 3, mRNA [NM_001197123]
TSC22D2	down	-1,38064	Homo sapiens TSC22 domain family, member 2 (TSC22D2), mRNA [NM_014779]
ACP2	down	-1,38085	Homo sapiens acid phosphatase 2, lysosomal (ACP2), transcript variant 1, mRNA [NM_001610]
LIMK1	down	-1,38269	Homo sapiens LIM domain kinase 1 (LIMK1), transcript variant 1, mRNA [NM_002314]
NDST2	down	-1,38284	Homo sapiens N-deacetylase/N-sulfotransferase (heparan glucosaminyl) 2 (NDST2), mRNA [NM_003635]
ZNF134	down	-1,38304	Homo sapiens zinc finger protein 134 (ZNF134), mRNA [NM_003435]
TRAF4	down	-1,38361	Homo sapiens TNF receptor-associated factor 4 (TRAF4), mRNA [NM_004295]
TMEM102	down	-1,38407	Homo sapiens transmembrane protein 102 (TMEM102), mRNA [NM_178518]
TIGD1	down	-1,38766	Homo sapiens tigger transposable element derived 1 (TIGD1), mRNA [NM_145702]
PDSS2	down	-1,39103	Homo sapiens prenyl (decaprenyl) diphosphate synthase, subunit 2 (PDSS2), mRNA [NM_020381]
RRN3P2	down	-1,3924	Homo sapiens RNA polymerase I transcription factor homolog (S, cerevisiae) pseudogene 2 (RRN3P2), non-coding RNA [NR_003369]
PILRB	down	-1,39264	Homo sapiens paired immunoglobin-like type 2 receptor beta (PILRB), transcript variant 3, mRNA [NM_178238]
ZNF564	down	-1,39501	Homo sapiens zinc finger protein 564 (ZNF564), mRNA [NM_144976]
ASXL2	down	-1,39618	Homo sapiens additional sex combs like 2 (Drosophila) (ASXL2), mRNA [NM_018263]
PASK	down	-1,39728	Homo sapiens PAS domain containing serine/threonine kinase [Source:HGNC Symbol;Acc:17270] [ENST00000493544]
C17orf63	down	-1,39734	Homo sapiens chromosome 17 open reading frame 63 (C17orf63), transcript variant 2, mRNA [NM_018182]
SPIN1	down	-1,39816	Homo sapiens spindlin 1 (SPIN1), mRNA [NM_006717]
SLC39A3	down	-1,4023	Homo sapiens solute carrier family 39 (zinc transporter), member 3 (SLC39A3), transcript variant 1, mRNA [NM_144564]
JMJD8	down	-1,40368	Homo sapiens jumonji domain containing 8 (JMJD8), mRNA [NM_001005920]
ADPRH	down	-1,40393	Homo sapiens ADP-ribosylarginine hydrolase (ADPRH), mRNA [NM_001125]
ZHX2	down	-1,4041	Homo sapiens zinc fingers and homeoboxes 2 (ZHX2), mRNA [NM_014943]
C17orf70	down	-1,40441	Homo sapiens chromosome 17 open reading frame 70 (C17orf70), transcript variant 2, mRNA [NM_025161]
MYPOP	down	-1,40477	Homo sapiens Myb-related transcription factor, partner of profilin (MYPOP), mRNA [NM_001012643]
ZNF641	down	-1,40671	Homo sapiens zinc finger protein 641 (ZNF641), transcript variant 1, mRNA [NM_152320]
HN1L	down	-1,40816	Homo sapiens hematological and neurological expressed 1-like (HN1L), mRNA [NM_144570]

ORAI1	down	-1,41026	Homo sapiens ORAI calcium release-activated calcium modulator 1 (ORAI1), mRNA [NM_032790]
SLC25A34	down	-1,41439	Homo sapiens solute carrier family 25, member 34 (SLC25A34), mRNA [NM_207348]
RG9MTD3	down	-1,41904	Homo sapiens RNA (guanine-9-) methyltransferase domain containing 3 (RG9MTD3), mRNA [NM_144964]
LRRC26	down	-1,41992	Homo sapiens leucine rich repeat containing 26 (LRRC26), mRNA [NM_001013653]
ACAP1	down	-1,42043	Homo sapiens ArfGAP with coiled-coil, ankyrin repeat and PH domains 1 (ACAP1), mRNA [NM_014716]
ARPC4-TTLL3	down	-1,42128	Homo sapiens ARPC4-TTLL3 readthrough (ARPC4-TTLL3), mRNA [NM_001198793]
SLX1A	down	-1,42265	Homo sapiens SLX1 structure-specific endonuclease subunit homolog A (S, cerevisiae) (SLX1A), transcript variant 1, mRNA [NM_001014999]
HNRNPH3	down	-1,4267	Homo sapiens heterogeneous nuclear ribonucleoprotein H3 (2H9) (HNRNPH3), transcript variant 2H9, mRNA [NM_012207]
TRAF2	down	-1,4299	Homo sapiens TNF receptor-associated factor 2 (TRAF2), mRNA [NM_021138]
ZC3H12D	down	-1,43056	Homo sapiens zinc finger CCCH-type containing 12D (ZC3H12D), mRNA [NM_207360]
LRFN4	down	-1,43612	Homo sapiens leucine rich repeat and fibronectin type III domain containing 4 (LRFN4), mRNA [NM_024036]
ITGB1BP1	down	-1,43717	Homo sapiens integrin beta 1 binding protein 1 (ITGB1BP1), transcript variant 2, mRNA [NM_022334]
PARP12	down	-1,4392	Homo sapiens poly (ADP-ribose) polymerase family, member 12 (PARP12), mRNA [NM_022750]
KIAA1919	down	-1,43959	Homo sapiens KIAA1919 (KIAA1919), mRNA [NM_153369]
KLHDC3	down	-1,4409	Homo sapiens kelch domain containing 3 (KLHDC3), transcript variant 1, mRNA [NM_057161]
TRIB3	down	-1,44175	Homo sapiens tribbles homolog 3 (Drosophila) (TRIB3), mRNA [NM_021158]
TRAPPC4	down	-1,44751	Homo sapiens trafficking protein particle complex 4 (TRAPPC4), mRNA [NM_016146]
INF2	down	-1,45022	Homo sapiens inverted formin, FH2 and WH2 domain containing (INF2), transcript variant 1, mRNA [NM_022489]
CDC34	down	-1,45032	Homo sapiens cell division cycle 34 homolog (S, cerevisiae) (CDC34), mRNA [NM_004359]
TUBGCP6	down	-1,45155	Homo sapiens tubulin, gamma complex associated protein 6 (TUBGCP6), mRNA [NM_020461]
TP53I3	down	-1,45205	Homo sapiens tumor protein p53 inducible protein 3 (TP53I3), transcript variant 1, mRNA [NM_004881]
BAP1	down	-1,45237	Homo sapiens BRCA1 associated protein-1 (ubiquitin carboxy-terminal hydrolase) (BAP1), mRNA [NM_004656]
TBL1X	down	-1,45471	Homo sapiens transducin (beta)-like 1X-linked (TBL1X), transcript variant 1, mRNA [NM_005647]
ALDH16A1	down	-1,45773	Homo sapiens aldehyde dehydrogenase 16 family, member A1 (ALDH16A1), transcript variant 1, mRNA [NM_153329]

C16orf7	down	-1,45805	Homo sapiens chromosome 16 open reading frame 7 (C16orf7), mRNA [NM_004913]
CRYAB	down	-1,4601	Homo sapiens crystallin, alpha B (CRYAB), mRNA [NM_001885]
COBRA1	down	-1,46398	Homo sapiens cofactor of BRCA1 (COBRA1), mRNA [NM_015456]
SOS2	down	-1,46525	Homo sapiens son of sevenless homolog 2 (Drosophila) (SOS2), mRNA [NM_006939]
FAM53B	down	-1,46537	Homo sapiens family with sequence similarity 53, member B (FAM53B), mRNA [NM_014661]
CTAGE4	down	-1,46609	Homo sapiens CTAGE family, member 4 (CTAGE4), mRNA [NM_198495]
HN1L	down	-1,47093	Homo sapiens hematological and neurological expressed 1-like (HN1L), mRNA [NM_144570]
IFI30	down	-1,47098	Homo sapiens interferon, gamma-inducible protein 30 (IFI30), mRNA [NM_006332]
HERC5	down	-1,47993	Homo sapiens hect domain and RLD 5 (HERC5), mRNA [NM_016323]
C19orf77	down	-1,48025	Homo sapiens chromosome 19 open reading frame 77 (C19orf77), mRNA [NM_001136503]
CERS6	down	-1,48091	Homo sapiens ceramide synthase 6 (CERS6), mRNA [NM_203463]
ZNF276	down	-1,48096	Homo sapiens zinc finger protein 276 (ZNF276), transcript variant a, mRNA [NM_001113525]
SFMBT1	down	-1,48392	Homo sapiens Scm-like with four mbt domains 1 (SFMBT1), transcript variant 1, mRNA [NM_001005159]
ZNF572	down	-1,48414	Homo sapiens zinc finger protein 572 (ZNF572), mRNA [NM_152412]
HLA-DOA	down	-1,48435	Homo sapiens major histocompatibility complex, class II, DO alpha (HLA-DOA), mRNA [NM_002119]
LAT	down	-1,48501	Homo sapiens linker for activation of T cells (LAT), transcript variant 1, mRNA [NM_014387]
LRRFIP2	down	-1,48739	Homo sapiens leucine rich repeat (in FLII) interacting protein 2 (LRRFIP2), transcript variant 2, mRNA [NM_017724]
SLC25A13	down	-1,48776	Homo sapiens solute carrier family 25, member 13 (citrin) (SLC25A13), nuclear gene encoding mitochondrial protein, transcript variant 2, mRNA [NM_014251]
ISCU	down	-1,48881	Homo sapiens iron-sulfur cluster scaffold homolog (E, coli) (ISCU), nuclear gene encoding mitochondrial protein, transcript variant 1, mRNA [NM_014301]
DOCK7	down	-1,50685	Homo sapiens dedicator of cytokinesis 7 (DOCK7), mRNA [NM_033407]
DLGAP4	down	-1,50702	Homo sapiens discs, large (Drosophila) homolog-associated protein 4 (DLGAP4), transcript variant 1, mRNA [NM_014902]
GUSBP1	down	-1,50717	Homo sapiens glucuronidase, beta pseudogene 1 (GUSBP1), transcript variant 3, non-coding RNA [NR_027028]
BTN2A2	down	-1,50772	Homo sapiens butyrophilin, subfamily 2, member A2 (BTN2A2), transcript variant 2, mRNA [NM_181531]
CTAGE5	down	-1,50809	Homo sapiens CTAGE family, member 5 (CTAGE5), transcript variant 4, mRNA [NM_203356]
TP53I3	down	-1,50902	Homo sapiens tumor protein p53 inducible protein 3 (TP53I3), transcript variant 1, mRNA [NM_004881]

DDX51	down	-1,51103	Homo sapiens DEAD (Asp-Glu-Ala-Asp) box polypeptide 51 (DDX51), mRNA [NM_175066]
DIS3L	down	-1,51258	Homo sapiens DIS3 mitotic control homolog (S, cerevisiae)-like (DIS3L), transcript variant 2, mRNA [NM_133375]
ATAD3B	down	-1,51285	Homo sapiens ATPase family, AAA domain containing 3B (ATAD3B), nuclear gene encoding mitochondrial protein, mRNA [NM_031921]
NCOA5	down	-1,51286	Homo sapiens nuclear receptor coactivator 5 (NCOA5), mRNA [NM_020967]
TELO2	down	-1,51932	Homo sapiens TEL2, telomere maintenance 2, homolog (S, cerevisiae) (TELO2), mRNA [NM_016111]
FANCA	down	-1,52477	Homo sapiens Fanconi anemia, complementation group A (FANCA), transcript variant 1, mRNA [NM_000135]
ITPR1	down	-1,53298	Homo sapiens inositol 1,4,5-trisphosphate receptor, type 1 (ITPR1), transcript variant 2, mRNA [NM_002222]
PDE6G	down	-1,53466	Homo sapiens phosphodiesterase 6G, cGMP-specific, rod, gamma (PDE6G), transcript variant 1, mRNA [NM_002602]
ZNF714	down	-1,53637	Homo sapiens zinc finger protein 714 (ZNF714), mRNA [NM_182515]
GANAB	down	-1,53681	Homo sapiens glucosidase, alpha; neutral AB (GANAB), transcript variant 3, mRNA [NM_198335]
UNC13D	down	-1,53727	Homo sapiens unc-13 homolog D (C, elegans) (UNC13D), mRNA [NM_199242]
DDX51	down	-1,54083	Homo sapiens DEAD (Asp-Glu-Ala-Asp) box polypeptide 51 (DDX51), mRNA [NM_175066]
AKTIP	down	-1,5439	Homo sapiens AKT interacting protein (AKTIP), transcript variant 1, mRNA [NM_001012398]
SEPT2	down	-1,54565	septin 2 [Source:HGNC Symbol;Acc:7729] [ENST00000473479]
ANKRD16	down	-1,54972	Homo sapiens ankyrin repeat domain 16 (ANKRD16), transcript variant 2, mRNA [NM_001009941]
PABPC1L	down	-1,55461	Homo sapiens poly(A) binding protein, cytoplasmic 1-like (PABPC1L), mRNA [NM_001124756]
SGSM3	down	-1,55563	Homo sapiens small G protein signaling modulator 3 (SGSM3), mRNA [NM_015705]
LGALS3	down	-1,5569	Homo sapiens lectin, galactoside-binding, soluble, 3 (LGALS3), transcript variant 1, mRNA [NM_002306]
C19orf20	down	-1,55735	Homo sapiens chromosome 19 open reading frame 20 (C19orf20), mRNA [NM_033513]
MICA	down	-1,56208	Homo sapiens MHC class I polypeptide-related sequence A (MICA), transcript variant 1 (allele MICA*001), mRNA [NM_000247]
ZDHHC14	down	-1,56316	Homo sapiens zinc finger, DHHC-type containing 14 (ZDHHC14), transcript variant 2, mRNA [NM_153746]
BTBD6	down	-1,56653	Homo sapiens BTB (POZ) domain containing 6 (BTBD6), mRNA [NM_033271]
LRRC16B	down	-1,56785	Homo sapiens leucine rich repeat containing 16B (LRRC16B), mRNA [NM_138360]
DCAF4	down	-1,56938	Homo sapiens DDB1 and CUL4 associated factor 4 (DCAF4), transcript variant 2, mRNA [NM_181340]

ADAMTS13	down	-1,57041	Homo sapiens ADAM metallopeptidase with thrombospondin type 1 motif, 13 (ADAMTS13), transcript variant 1, mRNA [NM_139025]
LOC728147	down	-1,57196	Homo sapiens cDNA FLJ38943 fis, clone NT2NE2017480, [AK096262]
MAZ	down	-1,57231	Homo sapiens MYC-associated zinc finger protein (purine-binding transcription factor) (MAZ), transcript variant 2, mRNA [NM_001042539]
PTPRCAP	down	-1,58201	Homo sapiens protein tyrosine phosphatase, receptor type, C-associated protein (PTPRCAP), mRNA [NM_005608]
SEMA7A	down	-1,58313	Homo sapiens semaphorin 7A, GPI membrane anchor (John Milton Hagen blood group) (SEMA7A), transcript variant 1, mRNA [NM_003612]
DNASE2	down	-1,59139	Homo sapiens deoxyribonuclease II, lysosomal (DNASE2), mRNA [NM_001375]
MARK2	down	-1,59252	Homo sapiens MAP/microtubule affinity-regulating kinase 2 (MARK2), transcript variant 4, mRNA [NM_001039469]
GINS3	down	-1,59926	Homo sapiens GINS complex subunit 3 (Psf3 homolog) (GINS3), transcript variant 2, mRNA [NM_022770]
RIN1	down	-1,60059	Homo sapiens Ras and Rab interactor 1 (RIN1), mRNA [NM_004292]
ATAD3C	down	-1,60464	Homo sapiens ATPase family, AAA domain containing 3C (ATAD3C), mRNA [NM_001039211]
TMEM139	down	-1,60543	Homo sapiens transmembrane protein 139 (TMEM139), transcript variant 1, mRNA [NM_153345]
AKT2	down	-1,60782	Homo sapiens v-akt murine thymoma viral oncogene homolog 2 (AKT2), transcript variant 1, mRNA [NM_001626]
RP11-165H20,1	down	-1,60814	Homo sapiens CHIA-like pseudogene (RP11-165H20,1), non-coding RNA [NR_003928]
P2RX5	down	-1,61038	Homo sapiens purinergic receptor P2X, ligand-gated ion channel, 5 (P2RX5), transcript variant 2, mRNA [NM_175080]
ANXA4	down	-1,61212	Homo sapiens annexin A4 (ANXA4), mRNA [NM_001153]
SCYL2	down	-1,61347	Homo sapiens SCY1-like 2 (S, cerevisiae) (SCYL2), mRNA [NM_017988]
POLR2C	down	-1,61428	Homo sapiens polymerase (RNA) II (DNA directed) polypeptide C, 33kDa (POLR2C), mRNA [NM_032940]
FAM59B	down	-1,62173	Homo sapiens family with sequence similarity 59, member B (FAM59B), transcript variant 2, mRNA [NM_001191033]
RGS12	down	-1,62192	Homo sapiens regulator of G-protein signaling 12 (RGS12), transcript variant 2, mRNA [NM_002926]
CCDC8	down	-1,62478	Homo sapiens coiled-coil domain containing 8 (CCDC8), mRNA [NM_032040]
CHKB	down	-1,6249	Homo sapiens choline kinase beta (CHKB), mRNA [NM_005198]
ORAI2	down	-1,6281	Homo sapiens ORAI calcium release-activated calcium modulator 2 (ORAI2), transcript variant 2, mRNA [NM_032831]
WASF2	down	-1,63345	Homo sapiens WAS protein family, member 2 (WASF2), transcript variant 1, mRNA [NM_006990]

AGSK1	down	-1,63446	Homo sapiens golgin subfamily A member 2-like (AGSK1), non-coding RNA [NR_026811]
C16orf59	down	-1,63604	Homo sapiens chromosome 16 open reading frame 59 (C16orf59), mRNA [NM_025108]
PTPRK	down	-1,6362	Homo sapiens protein tyrosine phosphatase, receptor type, K (PTPRK), transcript variant 2, mRNA [NM_002844]
ZCCHC2	down	-1,63835	Homo sapiens zinc finger, CCHC domain containing 2 (ZCCHC2), mRNA [NM_017742]
REC8	down	-1,64441	Homo sapiens REC8 homolog (yeast) (REC8), transcript variant 2, mRNA [NM_001048205]
LOC389906	down	-1,64486	Homo sapiens hypothetical LOC389906 (LOC389906), non-coding RNA [NR_034031]
ZGLP1	down	-1,64648	Homo sapiens zinc finger, GATA-like protein 1 (ZGLP1), mRNA [NM_001103167]
TMEM64	down	-1,6472	Homo sapiens transmembrane protein 64 (TMEM64), transcript variant 1, mRNA [NM_001008495]
KMO	down	-1,65291	Homo sapiens kynurenine 3-monooxygenase (kynurenine 3-hydroxylase) (KMO), mRNA [NM_003679]
ARRB1	down	-1,6534	Homo sapiens arrestin, beta 1 (ARRB1), transcript variant 1, mRNA [NM_004041]
POU3F1	down	-1,65362	Homo sapiens POU class 3 homeobox 1 (POU3F1), mRNA [NM_002699]
VASP	down	-1,65676	Homo sapiens vasodilator-stimulated phosphoprotein (VASP), mRNA [NM_003370]
NHLRC4	down	-1,66046	Homo sapiens NHL repeat containing 4 (NHLRC4), mRNA [NM_176677]
SOCS1	down	-1,66705	Homo sapiens suppressor of cytokine signaling 1 (SOCS1), mRNA [NM_003745]
TPRN	down	-1,67225	Homo sapiens taperin (TPRN), mRNA [NM_001128228]
HLA-A	down	-1,67327	Homo sapiens major histocompatibility complex, class I, A (HLA-A), transcript variant 1, mRNA [NM_002116]
ICOSLG	down	-1,67774	inducible T-cell co-stimulator ligand [Source:HGNC Symbol;Acc:17087] [ENST00000407780]
DHRS9	down	-1,69267	Homo sapiens dehydrogenase/reductase (SDR family) member 9 (DHRS9), transcript variant 1, mRNA [NM_005771]
MAEL	down	-1,69515	Homo sapiens maelstrom homolog (Drosophila) (MAEL), mRNA [NM_032858]
HERC2P2	down	-1,7007	Homo sapiens hect domain and RLD 2 pseudogene 2 (HERC2P2), non-coding RNA [NR_002824]
AGSK1	down	-1,70361	Homo sapiens golgin subfamily A member 2-like (AGSK1), non-coding RNA [NR_026811]
CLEC18B	down	-1,70472	Homo sapiens C-type lectin domain family 18, member B (CLEC18B), mRNA [NM_001011880]
BMP6	down	-1,70844	Homo sapiens bone morphogenetic protein 6 (BMP6), mRNA [NM_001718]
TGM1	down	-1,71898	Homo sapiens transglutaminase 1 (K polypeptide epidermal type I, protein-glutamine-gamma-glutamyltransferase) (TGM1), mRNA [NM_000359]
RPL13P5	down	-1,72115	Homo sapiens ribosomal protein L13 pseudogene 5 (RPL13P5), non-coding RNA [NR_002803]
TMEM8A	down	-1,72351	Homo sapiens transmembrane protein 8A (TMEM8A), mRNA [NM_021259]
LOC100270746	down	-1,73391	Homo sapiens hypothetical LOC100270746 (LOC100270746), non-coding RNA [NR_026776]
ZDHHC2	down	-1,73927	Homo sapiens zinc finger, DHHC-type containing 2 (ZDHHC2), mRNA [NM_016353]

CD151	down	-1,74633	Homo sapiens CD151 molecule (Raph blood group) (CD151), transcript variant 1, mRNA [NM_004357]
LYG1	down	-1,74748	Homo sapiens lysozyme G-like 1 (LYG1), mRNA [NM_174898]
TMTC4	down	-1,74889	Homo sapiens transmembrane and tetratricopeptide repeat containing 4 (TMTC4), transcript variant 1, mRNA [NM_032813]
CERCAM	down	-1,75005	Homo sapiens cerebral endothelial cell adhesion molecule (CERCAM), mRNA [NM_016174]
PDE4DIP	down	-1,75415	Homo sapiens phosphodiesterase 4D interacting protein (PDE4DIP), transcript variant 1, mRNA [NM_014644]
MFHAS1	down	-1,75519	Homo sapiens malignant fibrous histiocytoma amplified sequence 1 (MFHAS1), mRNA [NM_004225]
NIPSNAP3B	down	-1,76062	Homo sapiens nipsnap homolog 3B (C, elegans) (NIPSNAP3B), mRNA [NM_018376]
MCTP1	down	-1,76084	Homo sapiens multiple C2 domains, transmembrane 1 (MCTP1), transcript variant L, mRNA [NM_024717]
DMD	down	-1,76279	Homo sapiens dystrophin (DMD), transcript variant Dp140b, mRNA [NM_004021]
ANK3	down	-1,76375	Homo sapiens ankyrin 3, node of Ranvier (ankyrin G) (ANK3), transcript variant 1, mRNA [NM_020987]
ISYNA1	down	-1,76415	Homo sapiens inositol-3-phosphate synthase 1 (ISYNA1), transcript variant 1, mRNA [NM_016368]
KLF2	down	-1,76801	Homo sapiens Kruppel-like factor 2 (lung) (KLF2), mRNA [NM_016270]
IL1B	down	-1,76819	Homo sapiens interleukin 1, beta (IL1B), mRNA [NM_000576]
HERC6	down	-1,79744	Homo sapiens hect domain and RLD 6 (HERC6), transcript variant 1, mRNA [NM_017912]
ZNF879	down	-1,79857	Homo sapiens zinc finger protein 879 (ZNF879), mRNA [NM_001136116]
NPL	down	-1,80137	Homo sapiens N-acetylneuraminate pyruvate lyase (dihydrodipicolinate synthase) (NPL), transcript variant 2, mRNA [NM_001200050]
ST3GAL4	down	-1,80319	Homo sapiens ST3 beta-galactoside alpha-2,3-sialyltransferase 4 (ST3GAL4), mRNA [NM_006278]
LOC153684	down	-1,80971	Homo sapiens hypothetical LOC153684 (LOC153684), non-coding RNA [NR_015447]
MANEAL	down	-1,8114	Homo sapiens mannosidase, endo-alpha-like (MANEAL), transcript variant 1, mRNA [NM_001031740]
PDAP1	down	-1,81278	Homo sapiens PDGFA associated protein 1 (PDAP1), mRNA [NM_014891]
DOCK2	down	-1,81552	Homo sapiens dedicator of cytokinesis 2 (DOCK2), mRNA [NM_004946]
RNF157	down	-1,81625	Homo sapiens ring finger protein 157 (RNF157), mRNA [NM_052916]
PRR5	down	-1,82206	Homo sapiens proline rich 5 (renal) (PRR5), transcript variant 2, mRNA [NM_015366]
AMZ1	down	-1,82382	Homo sapiens archaelysin family metallopeptidase 1 (AMZ1), mRNA [NM_133463]
ATP6V0E2	down	-1,82969	Homo sapiens ATPase, H+ transporting V0 subunit e2 (ATP6V0E2), transcript variant 1, mRNA [NM_145230]

TPST1	down	-1,83989	Homo sapiens tyrosylprotein sulfotransferase 1 (TPST1), mRNA [NM_003596]
ZNF443	down	-1,83992	Homo sapiens zinc finger protein 443 (ZNF443), mRNA [NM_005815]
FBXO43	down	-1,84022	Homo sapiens F-box protein 43 (FBXO43), transcript variant 2, mRNA [NM_001029860]
HES6	down	-1,84147	Homo sapiens hairy and enhancer of split 6 (Drosophila) (HES6), transcript variant 1, mRNA [NM_018645]
ADI1	down	-1,84343	Homo sapiens acireductone dioxygenase 1 (ADI1), mRNA [NM_018269]
METRN	down	-1,84875	Homo sapiens meteorin, glial cell differentiation regulator (METRN), mRNA [NM_024042]
FJX1	down	-1,84983	Homo sapiens four jointed box 1 (Drosophila) (FJX1), mRNA [NM_014344]
NAPEPLD	down	-1,85366	Homo sapiens N-acyl phosphatidylethanolamine phospholipase D (NAPEPLD), transcript variant 1, mRNA [NM_001122838]
TSNAXIP1	down	-1,86826	Homo sapiens translin-associated factor X interacting protein 1 (TSNAXIP1), mRNA [NM_018430]
HES6	down	-1,87026	Homo sapiens hairy and enhancer of split 6 (Drosophila) (HES6), transcript variant 1, mRNA [NM_018645]
RAB3C	down	-1,87056	Homo sapiens RAB3C, member RAS oncogene family (RAB3C), mRNA [NM_138453]
C17orf76	down	-1,87319	Homo sapiens chromosome 17 open reading frame 76 (C17orf76), transcript variant 2, mRNA [NM_207387]
MMP9	down	-1,87885	Homo sapiens matrix metallopeptidase 9 (gelatinase B, 92kDa gelatinase, 92kDa type IV collagenase) (MMP9), mRNA [NM_004994]
LOC728743	down	-1,879	Homo sapiens zinc finger protein pseudogene (LOC728743), non-coding RNA [NR_027237]
COL18A1	down	-1,88971	Homo sapiens collagen, type XVIII, alpha 1 (COL18A1), transcript variant 1, mRNA [NM_030582]
MAPK12	down	-1,89093	Homo sapiens mitogen-activated protein kinase 12 (MAPK12), mRNA [NM_002969]
GSG1	down	-1,89164	Homo sapiens germ cell associated 1 (GSG1), transcript variant 1, mRNA [NM_031289]
LOC339975	down	-1,89339	Homo sapiens hypothetical protein LOC339975 (LOC339975), non-coding RNA [NR_038931]
HMGB3	down	-1,89834	Homo sapiens high mobility group box 3 (HMGB3), mRNA [NM_005342]
PDLIM2	down	-1,90116	Homo sapiens PDZ and LIM domain 2 (mystique) (PDLIM2), transcript variant 3, mRNA [NM_198042]
FFAR1	down	-1,90256	Homo sapiens free fatty acid receptor 1 (FFAR1), mRNA [NM_005303]
C2orf63	down	-1,91096	Homo sapiens chromosome 2 open reading frame 63 (C2orf63), transcript variant 1, mRNA [NM_152385]
DNAJB2	down	-1,91627	Homo sapiens DnaJ (Hsp40) homolog, subfamily B, member 2 (DNAJB2), transcript variant 1, mRNA [NM_001039550]
SEMA5A	down	-1,9181	Homo sapiens sema domain, seven thrombospondin repeats (type 1 and type 1-like), transmembrane domain (TM) and short cytoplasmic domain, (semaphorin) 5A (SEMA5A), mRNA [NM_003966]

CHST10	down	-1,91898	Homo sapiens carbohydrate sulfotransferase 10 (CHST10), mRNA [NM_004854]
SAP30L	down	-1,93002	Homo sapiens SAP30-like (SAP30L), transcript variant 1, mRNA [NM_024632]
POU4F1	down	-1,93908	Homo sapiens POU class 4 homeobox 1 (POU4F1), mRNA [NM_006237]
BICD1	down	-1,94452	Homo sapiens bicaudal D homolog 1 (Drosophila) (BICD1), transcript variant 1, mRNA [NM_001714]
UAP1L1	down	-1,95028	Homo sapiens UDP-N-acteylglucosamine pyrophosphorylase 1-like 1 (UAP1L1), mRNA [NM_207309]
PTPLA	down	-1,95487	protein tyrosine phosphatase-like (proline instead of catalytic arginine), member A [Source:HGNC Symbol;Acc:9639] [ENST00000326961]
SPATS2L	down	-1,95532	Homo sapiens spermatogenesis associated, serine-rich 2-like (SPATS2L), transcript variant 2, mRNA [NM_001100422]
RMI2	down	-1,9577	Homo sapiens RMI2, RecQ mediated genome instability 2, homolog (S, cerevisiae) (RMI2), mRNA [NM_152308]
MITF	down	-1,96647	Homo sapiens microphthalmia-associated transcription factor (MITF), transcript variant 1, mRNA [NM_198159]
MYB	down	-1,98347	Homo sapiens v-myb myeloblastosis viral oncogene homolog (avian) (MYB), transcript variant 2, mRNA [NM_005375]
C16orf74	down	-1,99568	Homo sapiens chromosome 16 open reading frame 74 (C16orf74), mRNA [NM_206967]
TGM2	down	-1,99765	Homo sapiens transglutaminase 2 (C polypeptide, protein-glutamine-gamma-glutamyltransferase) (TGM2), transcript variant 1, mRNA [NM_004613]
STK16	down	-1,99845	Homo sapiens serine/threonine kinase 16 (STK16), transcript variant 1, mRNA [NM_001008910]
CLEC11A	down	-2,00447	Homo sapiens C-type lectin domain family 11, member A (CLEC11A), mRNA [NM_002975]
JUP	down	-2,01565	Homo sapiens junction plakoglobin (JUP), transcript variant 1, mRNA [NM_002230]
CSAG3	down	-2,02578	Homo sapiens CSAG family, member 3 (CSAG3), transcript variant 2, mRNA [NM_001129828]
C20orf201	down	-2,02638	Homo sapiens chromosome 20 open reading frame 201 (C20orf201), mRNA [NM_001007125]
MGST3	down	-2,0374	Homo sapiens microsomal glutathione S-transferase 3 (MGST3), mRNA [NM_004528]
P2RX5	down	-2,04057	Homo sapiens purinergic receptor P2X, ligand-gated ion channel, 5 (P2RX5), transcript variant 1, mRNA [NM_002561]
C12orf35	down	-2,0462	Homo sapiens chromosome 12 open reading frame 35 (C12orf35), mRNA [NM_018169]
CMAHP	down	-2,04885	Homo sapiens cytidine monophospho-N-acetylneuraminic acid hydroxylase, pseudogene (CMAHP), transcript variant 1, non-coding RNA [NR_002174]
LYPD1	down	-2,05005	Homo sapiens LY6/PLAUR domain containing 1 (LYPD1), transcript variant 1, mRNA [NM_144586]
PLGLB1	down	-2,05112	Homo sapiens plasminogen-like B1 [Source:HGNC Symbol;Acc:9072] [ENST00000409310]
LMNB1	down	-2,05243	Homo sapiens lamin B1 (LMNB1), transcript variant 1, mRNA [NM_005573]
ERF	down	-2,05401	Homo sapiens Ets2 repressor factor (ERF), mRNA [NM_006494]

LOC283914	down	-2,05897	Homo sapiens hypothetical LOC283914 (LOC283914), non-coding RNA [NR_027079]
FAM43A	down	-2,05925	Homo sapiens family with sequence similarity 43, member A (FAM43A), mRNA [NM_153690]
KIAA1543	down	-2,06557	Homo sapiens KIAA1543 (KIAA1543), transcript variant 1, mRNA [NM_001080429]
EPSTI1	down	-2,06859	Homo sapiens epithelial stromal interaction 1 (breast) (EPSTI1), transcript variant 2, mRNA [NM_033255]
ADRB1	down	-2,08678	Homo sapiens adrenergic, beta-1-, receptor (ADRB1), mRNA [NM_000684]
RBMS1	down	-2,08967	Homo sapiens RNA binding motif, single stranded interacting protein 1 (RBMS1), transcript variant 1, mRNA [NM_016836]
CABLES1	down	-2,09106	Homo sapiens Cdk5 and Abl enzyme substrate 1 (CABLES1), transcript variant 1, mRNA [NM_138375]
ANKRD9	down	-2,10311	Homo sapiens ankyrin repeat domain 9 (ANKRD9), mRNA [NM_152326]
KIAA0649	down	-2,10748	Homo sapiens KIAA0649 (KIAA0649), mRNA [NM_014811]
LOC440900	down	-2,11134	Homo sapiens hypothetical LOC440900 (LOC440900), non-coding RNA [NR_034128]
SCML1	down	-2,13773	Homo sapiens sex comb on midleg-like 1 (Drosophila) (SCML1), transcript variant 1, mRNA [NM_001037540]
CACNB2	down	-2,14386	Homo sapiens calcium channel, voltage-dependent, beta 2 subunit (CACNB2), transcript variant 9, mRNA [NM_001167945]
CYP2J2	down	-2,15397	Homo sapiens cytochrome P450, family 2, subfamily J, polypeptide 2 (CYP2J2), mRNA [NM_000775]
BCAS3	down	-2,15481	Homo sapiens breast carcinoma amplified sequence 3 (BCAS3), transcript variant 2, mRNA [NM_017679]
IER5L	down	-2,16184	Homo sapiens immediate early response 5-like (IER5L), mRNA [NM_203434]
CMAHP	down	-2,16195	Homo sapiens cytidine monophospho-N-acetylneuraminic acid hydroxylase, pseudogene (CMAHP), transcript variant 1, non-coding RNA [NR_002174]
NPL	down	-2,16283	Homo sapiens N-acetylneuraminate pyruvate lyase (dihydrodipicolinate synthase) (NPL), transcript variant 3, mRNA [NM_001200056]
ITIH1	down	-2,17554	Homo sapiens inter-alpha (globulin) inhibitor H1 (ITIH1), transcript variant 1, mRNA [NM_002215]
FAM107B	down	-2,1823	Homo sapiens family with sequence similarity 107, member B (FAM107B), mRNA [NM_031453]
FAM19A5	down	-2,18434	Homo sapiens family with sequence similarity 19 (chemokine (C-C motif)-like), member A5 (FAM19A5), transcript variant 2, mRNA [NM_015381]
AP1S1	down	-2,1862	Homo sapiens adaptor-related protein complex 1, sigma 1 subunit (AP1S1), mRNA [NM_001283]
LOC100130776	down	-2,19477	Homo sapiens hypothetical LOC100130776 (LOC100130776), non-coding RNA [NR_027032]
SMOX	down	-2,19703	Homo sapiens spermine oxidase (SMOX), transcript variant 1, mRNA [NM_175839]
CLEC3B	down	-2,20117	Homo sapiens C-type lectin domain family 3, member B (CLEC3B), mRNA [NM_003278]

LLGL2	down	-2,20582	Homo sapiens lethal giant larvae homolog 2 (Drosophila) (LLGL2), transcript variant 3, mRNA [NM_001031803]
FAM89A	down	-2,20599	Homo sapiens family with sequence similarity 89, member A (FAM89A), mRNA [NM_198552]
BATF3	down	-2,2167	Homo sapiens basic leucine zipper transcription factor, ATF-like 3 (BATF3), mRNA [NM_018664]
MMP15	down	-2,21768	Homo sapiens matrix metallopeptidase 15 (membrane-inserted) (MMP15), mRNA [NM_002428]
GPER	down	-2,21956	Homo sapiens G protein-coupled estrogen receptor 1 (GPER), transcript variant 3, mRNA [NM_001039966]
COL5A1	down	-2,22054	Homo sapiens collagen, type V, alpha 1 (COL5A1), mRNA [NM_000093]
C9orf130	down	-2,22433	Homo sapiens chromosome 9 open reading frame 130 (C9orf130), transcript variant 1, non-coding RNA [NR_023390]
RAPGEF1	down	-2,22813	Homo sapiens Rap guanine nucleotide exchange factor (GEF) 1 (RAPGEF1), transcript variant 2, mRNA [NM_198679]
HNRNPUL2	down	-2,23097	Homo sapiens heterogeneous nuclear ribonucleoprotein U-like 2 (HNRNPUL2), mRNA [NM_001079559]
ADAM15	down	-2,23206	Homo sapiens ADAM metallopeptidase domain 15 (ADAM15), transcript variant 1, mRNA [NM_207191]
NOTO	down	-2,233	Homo sapiens notochord homeobox (NOTO), mRNA [NM_001134462]
CHMP4B	down	-2,2396	Homo sapiens chromatin modifying protein 4B (CHMP4B), mRNA [NM_176812]
C1orf15-NBL1	down	-2,24812	Homo sapiens C1ORF15-NBL1 readthrough (C1orf15-NBL1), transcript variant 1, mRNA [NM_001204088]
EMD	down	-2,27649	Homo sapiens emerin (EMD), mRNA [NM_000117]
ANXA6	down	-2,29411	Homo sapiens annexin A6 (ANXA6), transcript variant 1, mRNA [NM_001155]
LOC388564	down	-2,30574	Homo sapiens hypothetical protein LOC388564 (LOC388564), mRNA [NM_001190764]
GRIK4	down	-2,33659	Homo sapiens glutamate receptor, ionotropic, kainate 4 (GRIK4), mRNA [NM_014619]
NQO2	down	-2,33892	Homo sapiens NAD(P)H dehydrogenase, quinone 2 (NQO2), mRNA [NM_000904]
RGS22	down	-2,3442	Homo sapiens regulator of G-protein signaling 22 (RGS22), mRNA [NM_015668]
CALHM3	down	-2,34971	Homo sapiens calcium homeostasis modulator 3 (CALHM3), mRNA [NM_001129742]
ETV4	down	-2,36335	Homo sapiens ets variant 4 (ETV4), transcript variant 2, mRNA [NM_001079675]
LOC388796	down	-2,36633	Homo sapiens hypothetical LOC388796 (LOC388796), transcript variant 1, non-coding RNA [NR_015366]
CBR3	down	-2,37022	Homo sapiens carbonyl reductase 3 (CBR3), mRNA [NM_001236]
CLGN	down	-2,37104	Homo sapiens calmegin (CLGN), transcript variant 1, mRNA [NM_004362]
GPR68	down	-2,37312	Homo sapiens G protein-coupled receptor 68 (GPR68), transcript variant 2, mRNA [NM_003485]

CCDC58	down	-2,37984	Homo sapiens coiled-coil domain containing 58 (CCDC58), mRNA [NM_001017928]
C9orf140	down	-2,3892	Homo sapiens chromosome 9 open reading frame 140 (C9orf140), mRNA [NM_178448]
DLST	down	-2,41245	Homo sapiens dihydrolipoamide S-succinyltransferase (E2 component of 2-oxo-glutarate complex) (DLST), transcript variant 1, mRNA [NM_001933]
HOOK1	down	-2,42466	Homo sapiens hook homolog 1 (Drosophila) (HOOK1), mRNA [NM_015888]
MGMT	down	-2,42715	Homo sapiens O-6-methylguanine-DNA methyltransferase (MGMT), mRNA [NM_002412]
NT5E	down	-2,44706	Homo sapiens 5'-nucleotidase, ecto (CD73) (NT5E), transcript variant 1, mRNA [NM_002526]
F2RL3	down	-2,45234	Homo sapiens coagulation factor II (thrombin) receptor-like 3 (F2RL3), mRNA [NM_003950]
CD163L1	down	-2,46474	Homo sapiens CD163 molecule-like 1 (CD163L1), mRNA [NM_174941]
AMZ1	down	-2,47305	Homo sapiens archaelysin family metallopeptidase 1 (AMZ1), mRNA [NM_133463]
CLCF1	down	-2,47467	Homo sapiens cardiotrophin-like cytokine factor 1 (CLCF1), transcript variant 1, mRNA [NM_013246]
LOC723809	down	-2,49019	Homo sapiens hypothetical LOC723809 (LOC723809), non-coding RNA [NR_027374]
TNFSF12	down	-2,50692	Homo sapiens tumor necrosis factor (ligand) superfamily, member 12 (TNFSF12), transcript variant 1, mRNA [NM_003809]
TRIM71	down	-2,51023	Homo sapiens tripartite motif containing 71 (TRIM71), mRNA [NM_001039111]
SPINT2	down	-2,51249	Homo sapiens serine peptidase inhibitor, Kunitz type, 2 (SPINT2), transcript variant a, mRNA [NM_021102]
CMTM7	down	-2,51697	Homo sapiens CKLF-like MARVEL transmembrane domain containing 7 (CMTM7), transcript variant 1, mRNA [NM_138410]
PRR5	down	-2,53155	Homo sapiens proline rich 5 (renal) (PRR5), transcript variant 2, mRNA [NM_015366]
FGF9	down	-2,57219	Homo sapiens fibroblast growth factor 9 (glia-activating factor) (FGF9), mRNA [NM_002010]
HS3ST1	down	-2,57938	Homo sapiens heparan sulfate (glucosamine) 3-O-sulfotransferase 1 (HS3ST1), mRNA [NM_005114]
DNAJC7	down	-2,58666	Homo sapiens DnaJ (Hsp40) homolog, subfamily C, member 7 (DNAJC7), transcript variant 1, mRNA [NM_003315]
MGC16025	down	-2,59405	Homo sapiens hypothetical LOC85009 (MGC16025), non-coding RNA [NR_026664]
DKFZP564C152	down	-2,59873	Homo sapiens mRNA; cDNA DKFZp564C152 (from clone DKFZp564C152) [AL049980]
PWWP2B	down	-2,60485	Homo sapiens PWWP domain containing 2B (PWWP2B), transcript variant 1, mRNA [NM_138499]
GPT2	down	-2,60565	Homo sapiens glutamic pyruvate transaminase (alanine aminotransferase) 2 (GPT2), transcript variant 1, mRNA [NM_133443]
RAB38	down	-2,61915	Homo sapiens RAB38, member RAS oncogene family (RAB38), mRNA [NM_022337]
C5orf13	down	-2,62543	Homo sapiens chromosome 5 open reading frame 13 (C5orf13), transcript variant 11, mRNA [NM_001142483]

ADAM15	down	-2,6283	Homo sapiens ADAM metallopeptidase domain 15 (ADAM15), transcript variant 6, mRNA [NM_207197]
ANKRD2	down	-2,64915	Homo sapiens ankyrin repeat domain 2 (stretch responsive muscle) (ANKRD2), transcript variant 1, mRNA [NM_020349]
TCL1B	down	-2,70196	Homo sapiens T-cell leukemia/lymphoma 1B (TCL1B), transcript variant 1, mRNA [NM_004918]
FAM22A	down	-2,72027	Homo sapiens family with sequence similarity 22, member A (FAM22A), mRNA [NM_001099338]
WNT3A	down	-2,74529	Homo sapiens wingless-type MMTV integration site family, member 3A (WNT3A), mRNA [NM_033131]
F13A1	down	-2,76153	Homo sapiens coagulation factor XIII, A1 polypeptide (F13A1), mRNA [NM_000129]
KIAA1324	down	-2,8322	Homo sapiens KIAA1324 (KIAA1324), mRNA [NM_020775]
NT5E	down	-2,84466	Homo sapiens 5'-nucleotidase, ecto (CD73) (NT5E), transcript variant 1, mRNA [NM_002526]
NKD2	down	-2,87794	Homo sapiens naked cuticle homolog 2 (Drosophila) (NKD2), mRNA [NM_033120]
COMP	down	-2,87871	Homo sapiens cartilage oligomeric matrix protein (COMP), mRNA [NM_000095]
CYP1A1	down	-2,88422	Homo sapiens cytochrome P450, family 1, subfamily A, polypeptide 1 (CYP1A1), mRNA [NM_000499]
PTGER4	down	-2,89534	Homo sapiens prostaglandin E receptor 4 (subtype EP4) (PTGER4), mRNA [NM_000958]
CYP7B1	down	-2,95412	Homo sapiens cytochrome P450, family 7, subfamily B, polypeptide 1 (CYP7B1), mRNA [NM_004820]
NAV2	down	-2,95675	Homo sapiens neuron navigator 2 (NAV2), transcript variant 1, mRNA [NM_182964]
FOX06	down	-2,96692	forkhead box O6 [Source:HGNC Symbol;Acc:24814] [ENST00000372591]
EMID1	down	-2,97167	Homo sapiens EMI domain containing 1 (EMID1), mRNA [NM_133455]
GADD45G	down	-2,97206	Homo sapiens growth arrest and DNA-damage-inducible, gamma (GADD45G), mRNA [NM_006705]
HEBP1	down	-3,0158	Homo sapiens heme binding protein 1 (HEBP1), mRNA [NM_015987]
LOC96610	down	-3,01946	Homo sapiens BMS1 homolog, ribosome assembly protein (yeast) pseudogene (LOC96610), non-coding RNA [NR_027293]
SLC25A29	down	-3,06921	Homo sapiens solute carrier family 25, member 29 (SLC25A29), nuclear gene encoding mitochondrial protein, mRNA [NM_001039355]
TEX19	down	-3,11051	Homo sapiens testis expressed 19 (TEX19), mRNA [NM_207459]
TNFSF12	down	-3,11871	Homo sapiens tumor necrosis factor (ligand) superfamily, member 12 (TNFSF12), transcript variant 1, mRNA [NM_003809]
AKAP7	down	-3,13968	Homo sapiens A kinase (PRKA) anchor protein 7 (AKAP7), transcript variant gamma, mRNA [NM_016377]
MRC2	down	-3,157	Homo sapiens mannose receptor, C type 2 (MRC2), mRNA [NM_006039]
RPS6KA6	down	-3,15714	Homo sapiens ribosomal protein S6 kinase, 90kDa, polypeptide 6 (RPS6KA6), mRNA [NM_014496]

RASGRP2	down	-3,20074	Homo sapiens RAS guanyl releasing protein 2 (calcium and DAG-regulated) (RASGRP2), transcript variant 2, mRNA [NM_153819]
HDX	down	-3,21368	Homo sapiens highly divergent homeobox (HDX), transcript variant 2, mRNA [NM_144657]
EMB	down	-3,23018	Homo sapiens embigin (EMB), mRNA [NM_198449]
BHLHE22	down	-3,2424	Homo sapiens basic helix-loop-helix family, member e22 (BHLHE22), mRNA [NM_152414]
IGSF1	down	-3,25907	Homo sapiens immunoglobulin superfamily, member 1 (IGSF1), transcript variant 1, mRNA [NM_001555]
OR7D2	down	-3,28622	Homo sapiens olfactory receptor, family 7, subfamily D, member 2 (OR7D2), mRNA [NM_175883]
CA2	down	-3,30555	Homo sapiens carbonic anhydrase II (CA2), mRNA [NM_000067]
BMP2	down	-3,31307	Homo sapiens bone morphogenetic protein 2 (BMP2), mRNA [NM_001200]
RGAG4	down	-3,35361	Homo sapiens retrotransposon gag domain containing 4 (RGAG4), mRNA [NM_001024455]
FAM184A	down	-3,35832	Homo sapiens family with sequence similarity 184, member A (FAM184A), transcript variant 1, mRNA [NM_024581]
CEBPA	down	-3,39726	Homo sapiens CCAAT/enhancer binding protein (C/EBP), alpha (CEBPA), mRNA [NM_004364]
LRRC16A	down	-3,41134	Homo sapiens leucine rich repeat containing 16A (LRRC16A), transcript variant 1, mRNA [NM_017640]
NEFH	down	-3,45778	Homo sapiens neurofilament, heavy polypeptide (NEFH), mRNA [NM_021076]
UBE2QL1	down	-3,46233	Homo sapiens ubiquitin-conjugating enzyme E2Q family-like 1 (UBE2QL1), mRNA [NM_001145161]
JSRP1	down	-3,47539	Homo sapiens junctional sarcoplasmic reticulum protein 1 (JSRP1), mRNA [NM_144616]
STK32C	down	-3,49328	Homo sapiens serine/threonine kinase 32C (STK32C), mRNA [NM_173575]
DDAH1	down	-3,50337	Homo sapiens dimethylarginine dimethylaminohydrolase 1 (DDAH1), transcript variant 1, mRNA [NM_012137]
NPY	down	-3,51553	Homo sapiens neuropeptide Y (NPY), mRNA [NM_000905]
ZDHHC14	down	-3,54196	Homo sapiens zinc finger, DHHC-type containing 14 (ZDHHC14), transcript variant 2, mRNA [NM_153746]
COL16A1	down	-3,56352	Homo sapiens collagen, type XVI, alpha 1 (COL16A1), mRNA [NM_001856]
SNX7	down	-3,5824	Homo sapiens sorting nexin 7 (SNX7), transcript variant 1, mRNA [NM_015976]
ASB9	down	-3,62059	Homo sapiens ankyrin repeat and SOCS box containing 9 (ASB9), transcript variant 1, mRNA [NM_001031739]
WWOX	down	-3,63247	Homo sapiens WW domain containing oxidoreductase (WWOX), transcript variant 2, mRNA [NM_130791]
B3GALTL	down	-3,67616	Homo sapiens beta 1,3-galactosyltransferase-like (B3GALTL), mRNA [NM_194318]
ASPA	down	-3,74551	Homo sapiens aspartoacylase (ASPA), transcript variant 1, mRNA [NM_000049]

TMEM119	down	-3,80439	Homo sapiens transmembrane protein 119 (TMEM119), mRNA [NM_181724]
POU3F3	down	-3,80767	Homo sapiens POU class 3 homeobox 3 (POU3F3), mRNA [NM_006236]
CXCL10	down	-3,81237	Homo sapiens chemokine (C-X-C motif) ligand 10 (CXCL10), mRNA [NM_001565]
PHOSPHO2-KLHL23	down	-3,81703	Homo sapiens PHOSPHO2-KLHL23 readthrough (PHOSPHO2-KLHL23), mRNA [NM_001199290]
CA13	down	-3,87698	Homo sapiens carbonic anhydrase XIII (CA13), mRNA [NM_198584]
NCKAP5	down	-3,91091	Homo sapiens NCK-associated protein 5 (NCKAP5), transcript variant 1, mRNA [NM_207363]
CCRL1	down	-3,9716	Homo sapiens chemokine (C-C motif) receptor-like 1 (CCRL1), transcript variant 1, mRNA [NM_178445]
TUBB6	down	-3,99816	Homo sapiens tubulin, beta 6 (TUBB6), mRNA [NM_032525]
FXYD2	down	-4,11389	Homo sapiens FXYD domain containing ion transport regulator 2 (FXYD2), transcript variant a, mRNA [NM_001680]
IGSF1	down	-4,13084	Homo sapiens immunoglobulin superfamily, member 1 (IGSF1), transcript variant 2, mRNA [NM_205833]
HEY1	down	-4,2047	Homo sapiens hairy/enhancer-of-split related with YRPW motif 1 (HEY1), transcript variant 2, mRNA [NM_001040708]
AEBP1	down	-4,3016	Homo sapiens AE binding protein 1 (AEBP1), mRNA [NM_001129]
PLEKHA4	down	-4,30389	Homo sapiens pleckstrin homology domain containing, family A (phosphoinositide binding specific) member 4 (PLEKHA4), transcript variant 1, mRNA [NM_020904]
TNFRSF11A	down	-4,35228	Homo sapiens tumor necrosis factor receptor superfamily, member 11a, NFKB activator (TNFRSF11A), mRNA [NM_003839]
LFNG	down	-4,37759	Homo sapiens LFNG O-fucosylpeptide 3-beta-N-acetylglucosaminyltransferase (LFNG), transcript variant 1, mRNA [NM_001040167]
HES5	down	-4,39227	Homo sapiens hairy and enhancer of split 5 (Drosophila) (HES5), mRNA [NM_001010926]
C22orf36	down	-4,42682	Homo sapiens chromosome 22 open reading frame 36 (C22orf36), mRNA [NM_207644]
TNRC18	down	-4,44182	Homo sapiens trinucleotide repeat containing 18 (TNRC18), mRNA [NM_001080495]
C1DP3	down	-4,50735	Homo sapiens C1D nuclear receptor corepressor pseudogene 3 [Source:HGNC Symbol;Acc:31661] [ENST00000412019]
GTSF1	down	-4,59979	Homo sapiens gametocyte specific factor 1 (GTSF1), mRNA [NM_144594]
FXYD2	down	-4,65928	Homo sapiens FXYD domain containing ion transport regulator 2 (FXYD2), transcript variant b, mRNA [NM_021603]
GAL3ST1	down	-4,80014	Homo sapiens galactose-3-O-sulfotransferase 1 (GAL3ST1), mRNA [NM_004861]
TNN	down	-4,87032	Homo sapiens tenascin N (TNN), mRNA [NM_022093]
RAPH1	down	-4,88931	Homo sapiens Ras association (RalGDS/AF-6) and pleckstrin homology domains 1 (RAPH1), transcript variant 1, mRNA [NM_213589]

RIMS3	down	-4,90364	Homo sapiens regulating synaptic membrane exocytosis 3 (RIMS3), mRNA [NM_014747]
TIAM1	down	-4,94077	Homo sapiens T-cell lymphoma invasion and metastasis 1 (TIAM1), mRNA [NM_003253]
CA4	down	-4,94146	Homo sapiens carbonic anhydrase IV (CA4), mRNA [NM_000717]
CXCR4	down	-5,15257	Homo sapiens chemokine (C-X-C motif) receptor 4 (CXCR4), transcript variant 1, mRNA [NM_001008540]
DDC	down	-5,2511	Homo sapiens dopa decarboxylase (aromatic L-amino acid decarboxylase) (DDC), transcript variant 2, mRNA [NM_000790]
CXCL10	down	-5,25424	Homo sapiens chemokine (C-X-C motif) ligand 10 (CXCL10), mRNA [NM_001565]
KCTD15	down	-5,43917	Homo sapiens potassium channel tetramerisation domain containing 15 (KCTD15), transcript variant 1, mRNA [NM_024076]
RASGRP2	down	-5,58616	Homo sapiens RAS guanyl releasing protein 2 (calcium and DAG-regulated) (RASGRP2), transcript variant 2, mRNA [NM_153819]
LRRC31	down	-5,73339	Homo sapiens leucine rich repeat containing 31 (LRRC31), mRNA [NM_024727]
FRZB	down	-5,73708	Homo sapiens frizzled-related protein (FRZB), mRNA [NM_001463]
PTPLA	down	-5,99137	Homo sapiens protein tyrosine phosphatase-like (proline instead of catalytic arginine), member A (PTPLA), mRNA [NM_014241]
TPM2	down	-6,30416	Homo sapiens tropomyosin 2 (beta) (TPM2), transcript variant 2, mRNA [NM_213674]
TNFRSF21	down	-6,62652	Homo sapiens tumor necrosis factor receptor superfamily, member 21 (TNFRSF21), mRNA [NM_014452]
VIL1	down	-7,85588	Homo sapiens villin 1 (VIL1), mRNA [NM_007127]
FAM59A	down	-7,9309	Homo sapiens family with sequence similarity 59, member A (FAM59A), transcript variant 2, mRNA [NM_022751]
S100A10	down	-8,30112	Homo sapiens S100 calcium binding protein A10 (S100A10), mRNA [NM_002966]
LOC401847	down	-8,8908	PREDICTED: Homo sapiens hypothetical protein LOC401847 (LOC401847), mRNA [XM_001718104]
CHRM4	down	-9,00904	Homo sapiens cholinergic receptor, muscarinic 4 (CHRM4), mRNA [NM_000741]
FAM134B	down	-9,31962	Homo sapiens family with sequence similarity 134, member B (FAM134B), transcript variant 1, mRNA [NM_001034850]
CYP27A1	down	-9,33209	Homo sapiens cytochrome P450, family 27, subfamily A, polypeptide 1 (CYP27A1), nuclear gene encoding mitochondrial protein, mRNA [NM_000784]
MYRIP	down	-9,81639	Homo sapiens myosin VIIA and Rab interacting protein (MYRIP), mRNA [NM_015460]
TPM2	down	-12,8464	Homo sapiens tropomyosin 2 (beta) (TPM2), transcript variant 2, mRNA [NM_213674]
BAMBI	down	-36,3594	Homo sapiens BMP and activin membrane-bound inhibitor homolog (Xenopus laevis) (BAMBI), mRNA [NM_012342]

Supplementary Table 4. Pathways affected in *SMC1A* mutated cells (*P*<0.05)

**Pathway**	***P*-value**
Hs_Insulin_Signaling_WP481_42706	9,86E-07
Hs_Eukaryotic_Transcription_Initiation_WP405_41116	2,15E-04
Hs_Cholesterol_Biosynthesis_WP197_41229	2,70E-04
Hs_TCA_Cycle_WP78_45096	0,001596324
Hs_Kit_receptor_signaling_pathway_WP304_44603	0,001959281
Hs_Signaling_of_Hepatocyte_Growth_Factor_Receptor_WP313_41206	0,002236154
Hs_Acetylcholine_Synthesis_WP528_38919	0,002259841
Hs_Glycolysis_and_Gluconeogenesis_WP534_41077	0,00284873
Hs_Integrin-mediated_cell_adhesion_WP185_41226	0,002890552
Hs_MAPK_signaling_pathway_WP382_41048	0,003055366
Hs_Glycolysis_and_Gluconeogenesis_WP534_51732	0,003645324
Hs_Cytoplasmic_Ribosomal_Proteins_WP477_41118	0,003744046
Hs_Notch_Signaling_Pathway_WP61_45344	0,003802155
Hs_Calcium_Regulation_in_the_Cardiac_Cell_WP536_41204	0,004048791
Hs_B_Cell_Receptor_Signaling_Pathway_WP23_44620	0,005151478
Hs_Prostate_Cancer_WP2263_53136	0,005629781
Hs_TCA_Cycle_WP78_47741	0,005852571
Hs_EGF-EGFR_Signaling_Pathway_WP437_44600	0,006422873
Hs_RANKL-RANK_Signaling_Pathway_WP2018_44630	0,007807028
Hs_Prostaglandin_Synthesis_and_Regulation_WP98_45273	0,008769475
Hs_Serotonin_HTR1_Group_and_FOS_Pathway_WP722_46093	0,010042358
Hs_TCR_Signaling_Pathway_WP69_45093	0,012222474
Hs_Glycogen_Metabolism_WP500_41230	0,016392842
Hs_Regulation_of_Actin_Cytoskeleton_WP51_45278	0,019081526

Hs_AGE-RAGE_pathway_WP2324_53109	0,020217197
Hs_SIDS_Susceptibility_Pathways_WP706_49768	0,021444673
Hs_EPO_Receptor_Signaling_WP581_41162	0,021946903
Hs_SIDS_Susceptibility_Pathways_WP706_52941	0,022891477
Hs_Focal_Adhesion_WP306_41071	0,023204237
Hs_TWEAK_Signaling_Pathway_WP2036_46204	0,030158525
Hs_Myometrial_Relaxation_and_Contraction_Pathways_WP289_45373	0,030161388
Hs_mRNA_processing_WP411_45374	0,035718203
Hs_Glucuronidation_WP698_38927	0,041485265
Hs_Tryptophan_metabolism_WP465_43616	0,042452045
Hs_Adipogenesis_WP236_41040	0,044835843
Hs_Translation_Factors_WP107_41026	0,049577996
Hs_Toll-like_receptor_signaling_pathway_WP75_41115	0,05077824

Supplementary Table 5. List of primer sequences used for microarray validation by qRT-PCR.

**Gene**	**Forward primer sequence (5’-3’)**	**Reverse primer sequence (5’-3’)**
RALY	CGACAGGCTCTTCGACTACC	TCTGTGTCAGCTCCGTCTTG
UBE2I	CAGGAGAGGAAAGCATGGAG	TCGGGTGAAATAATGGTGGT
SOCS1	AGAGCTTCGACTGCCTCTTC	AGGGGAAGGAGCTCAGGTAG
ILF3	CTGGTGCTGCTGTGTAAGGA	AGGGACAATGGAGGCTCTTT
DNM2	TGATCCTGCAGTTCATCAGC	TCTTCTCAACGGGAGCAACT
FOXM1	CTGTTCAAAATGCCCCAAGT	TGCTGTGATGATGCTGTGAA
CXCR4	CCGTGGCAAACTGGTACTTT	GACGCCAACATAGACCACCT
FAM43A	TGCACATCACTAGCGAGGAC	TCACCGTCAGCTTCATCTTG
RASA1	TGCTGTTGCTGGACCTAGTG	TAGTTGGAGGAGCGGTCAAC
CHCHD2	AGGGTTTCAATGAGGTGCTG	TGCCCAGTCCCAGATTCTAC
IL2RB	CCTGTGTCTGGAGCCAAGAT	GGGTGACGATGTCAACTGTG
IRF8	AGTGGCTGATCGAGCAGATT	ACTGCCCAGGCCTTAAAAAT
RAB13	GAGCCATGGGCATTATCCTA	CCTTCTGCACCTTCCTCTTG
DYRK13	CCGGATTTTGGAGCATCTTA	CAAGATGGACTGGGCAAACT
STAM2	CGCTGCAAGAACAGAAACAG	CACCAATTGGCATCACTGTC
LIPA	CTGGTTTTGACGTGTGGATG	TAGCCAGCTCAGGGATCTGT
GADPH	AGAAGGCTGGGGCTCATTT	AGTCTTCTGGGTGGCAGTGAT

Supplementary Table 6. Primers used for ChIP-qPCR .

Gene	Forward primer sequence (5'-3')	Reverse primer sequence (5'-3')	Chromosome position
RALY	TAAAAGCGAAAGGACCAGGA	AGTGGGAGGAAGATGGAAGC	chr20:33,993,082-33,993,325
	CAGTCCCTCCTCCCTTTCAG	TCCTTCTCTGTCTCCTGGGA	chr20:33,993,585-33,993,777
SOCS1	GGAGGAGGGAGGGGAGTC	CGGAGAAAGGCTGTGCTG	chr16:11,255,899-11,256,047
	AGAGCTTCGACTGCCTCTTC	AGGGGAAGGAGCTCAGGTAG	chr16:11,254,854-11,255,054
ILF3	GCCATTGTCACATGATGGAG	AGGGGCGAAAAGAGATAAGG	chr19:10,654,072-10,654,215
	GACTACGCACGCAAACACTC	GTGCGGGAACACGTGACC	chr19:10,764,808-10,764,975
UBE2I	GCGGGAATGAGTGAGAGT	CATTCGATCCCTCCATCA	chr16:1,308,343-1,308,605
	CGACGCTTCAGAGGATCCTT	GAGCGTCTGTAAACTCCCCT	chr16:1,309,178-1,309,387
	GAGGAGGACAAGGACTGGAG	CGGGTGTGAGCAGTTTGAAA	chr16:1,370,243-1,370,413
DNM2	TTGCAGTCTGGGAACTGAGA	AGCCAACCAGGCAAAAGAG	chr19:10,717,402-10,717,661
	TACTCAACCATAGGCCCGTG	CCTCATCCGGTTCTCAGGC	chr19:10,717,937-10,718,093
FOXM1	CCACTTCTTCCCCCACAAG	CAGTTTGTTCCGCTGTTTGA	chr12:2,877,134-2,877,322
	CAGCACTGGAGAGACTGTCA	GCCATCAACAGCACTGAGAG	chr12:2,868,501-2,868,667
IL2RB	GTGTTGGGTAGCCTGTGTGA	GCCTGAAACTCCTCAAGCAC	chr22:37,150,027-37,150,244
	ACATCACCTGGCTGAGACAT	CCCCTCTATCCTCTGCACAG	chr22:37,149,818-37,149,982
IRF8	ATTTCTCGGAAAGCAGAGCA	GCCCACTGTGCCTACCTG	chr16:85,899,023-85,899,238
	CCTCATTTCCCAGTTTTGGA	TGCACCCCATGCTATAAACA	chr16:85,910,463-85,910,618
DYRK3	CAGTCGGGAGCGAAAGTG	GTGCCTCCCATCTCCTAGC	chr1:206,635,552-206,635,714
	TCTGCAATCCTTCTGAACCAC	AGTAGCACTTCCTCCTTTCCA	chr1:206,637,721-206,637,820
RASA1	GAGTAGAGCGGGCTTCAACA	ACCCAGAGTTCCAGCCACT	chr5:87,268,433-87,268,637
	AAACAGGCAAAGGAAAACGTT	ACCAGTCTCTTACCTGCCAA	chr5:87,363,342-87,363,517
CHCHD2	CGGATGAACCACAGGAACTT	CAGCACACAGGCAGAGAACT	chr7:56,106,526-56,106,682
RAB13	CACATGGGTCCCCATAAGAG	ATGGGTCCCAATTCACTTTG	chr1:153,986,839-153,987,035
	AAGTTTTCCTCCCTCTCCGC	AATAGCGAGAGAGACAGGCC	chr1:153958816-153958983
GAPDH	AGTGTCCTGCTGCCCACAGT	TAGCCGGGCCCTACTTTCTC	chr12:6,534,207-6,534,374
PRDX1	GAGAGCTCTTGCGCGTCTTG	CGCTCAAGCAGTCTCGGACT	chr1:45,521,669-45,521,896
GENE DESERT 1	GGCACAGGGCATTTCATTAT	AGGAGAAGGGCTCAAAGGAG	chr2:104,020,711-104,020,878
GENE DESERT 2	TGCCATGCGTTGAAAATATCC	TGTTTCTGAAGTTGCCAAGC	chr7:27,207,173-27,207,346

Supplementary figure 1. **Validation of microarray data from *SCM1A*-mutated CdLS and healthy control cell lines (CT) by qRT-PCR**. (**A**) Expression levels of downregulated genes and (**B**) upregulated genes in CdLS and control cell lines. The results are the averages of three independent experiments. For statistical analysis, a two tailed Student's *t*-test was performed.*$p < 0.05$.

Supplementary figure 2. Analysis of Pol II recruitment at the promoter regions by ChIP-qPCR using anti-Pol II antibody (CTD domain). (**A**) Pol II recruitment decreases in CdLS cell lines compared to control cell lines at the promoter regions of downregulated genes in *SMC1A*-mutated CdLS cell lines (**B**) Pol II enrichment does not significantly change in CdLS cell lines at the promoter regions of upregulated genes. I: genes virtually active in all cell types, II: genes preferentially expressed in lymphoblastoid cells, and III: our best targets based on ChIP-Seq enrichment. Since no difference was found, control cell line data were pooled. IgG was used as negative control. Results represent three independent ChIP assays normalized to genomic regions without Pol II enrichment. The average values of the experiments and the relative standard errors are shown.* $p < 0.05$

Supplementary figure 3. **Pol II binding status analysis in *SMC1A*-mutated CdLS and control cell lines**. (**A**) ChIP-qPCR evaluation of Pol II (CTD domain) at the promoters of genes whose expression was not affected by *SMC1A* mutations and (**B**) in gene desert regions. Since no difference was found, control cell line data were pooled. Results represent three independent ChIP assays and the average values of the experiments and the relative standard errors are shown.

Supplementary figure 4. **4H8 antibody recognizes the phosphorylated form Ser2P.** The specificity of 4H8 antibody was assayed by co-immunoprecipitating (IP) phospho-forms of Pol II from a total protein extract deriving a normal lymphoblastoid cell line. Western blotting showed that 4H8 recognizes phospho-forms of Pol II. A signal corresponding to Ser2P was detected when the same membrane was incubated with an antibody against Ser2P. No signal was detected in the IPs using IgG-coated beads (-).

Supplementary figure 5. **Pol II Ser2P distribution in normal human lymphoblastoid cells**. Binding of elongating Pol II represented as percentage of sites relative to promoter, gene body and downstream region.

Supplementary figure 6. **Analysis of the phosphorylated Pol II occupancy using anti-Pol II Ser5P-Ser2P antibody**. (**A**) Elongating form of Pol II decreases in the CdLS cell lines compared to control cell lines at the promoter of downregulated genes and (**B**) upregulated genes. I: genes virtually active in all cell types, II: genes preferentially expressed in lymphoblastoid cells, and III: our best targets based on ChIP-Seq enrichment. Since no difference was found, control cell line data were pooled. IgG was used as negative control**.** Results represent three independent ChIP assays normalized to genomic regions without Pol II enrichment. The average values of the experiments and the relative standard errors are shown.* $p < 0.05$

Supplementary figure 7. Analysis of occupancy of the phosphorylated Pol II at the promoter regions using anti-Pol II Ser2P antibody. (**A**) Pol II Ser2 decreases in CdLS cell lines with respect to control cell lines in downregulated genes and (**B**) upregulated genes. I: genes virtually active in all cell types, II: genes preferentially expressed in lymphoblastoid cells, and III: our best targets based on ChIP-Seq enrichment. Since no difference was found, control cell line data were pooled. IgG was used as negative control**.** Results represent three independent ChIP assays normalized to genomic regions without Pol II enrichment. The average values of the experiments and the relative standard errors are shown.* $p < 0.05$.

Supplementary figure 8. Analysis of cohesin occupancy at the promoter regions. (**A**) Cohesin binding increases in the CdLS cell lines compared to control cell lines at the promoter of downregulated genes and (**B**) upregulated genes. I: genes virtually active in all cell types, II: genes preferentially expressed in lymphoblastoid cells, and III: our best targets based on ChIP-Seq enrichment. Since no difference was found, control cell line data were pooled. IgG was used as negative control. Results represent three independent ChIP assays and the average values of the experiments and the relative standard errors are shown.* $p < 0.05$.

Supplementary figure 1

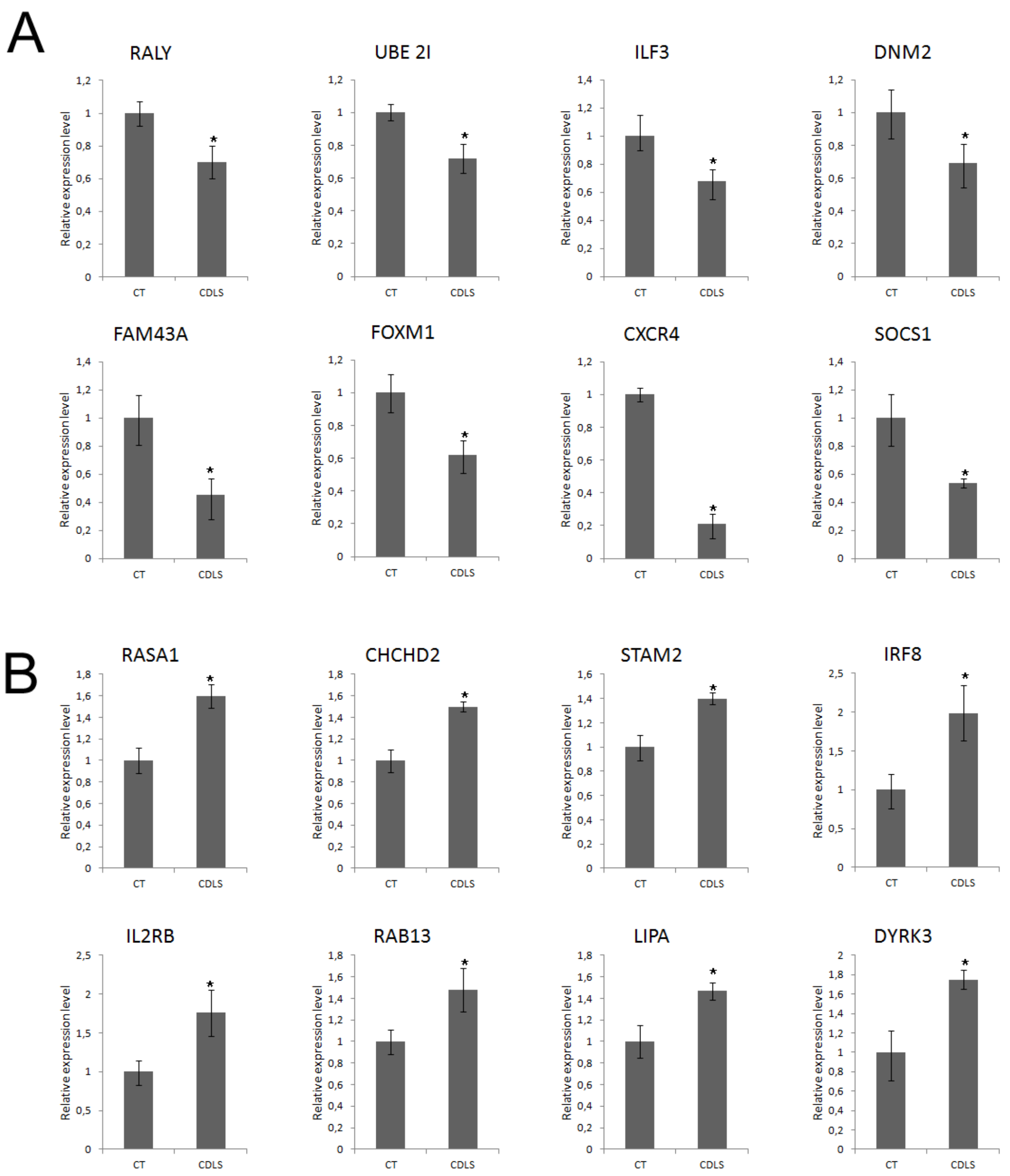

Supplementary figure 2

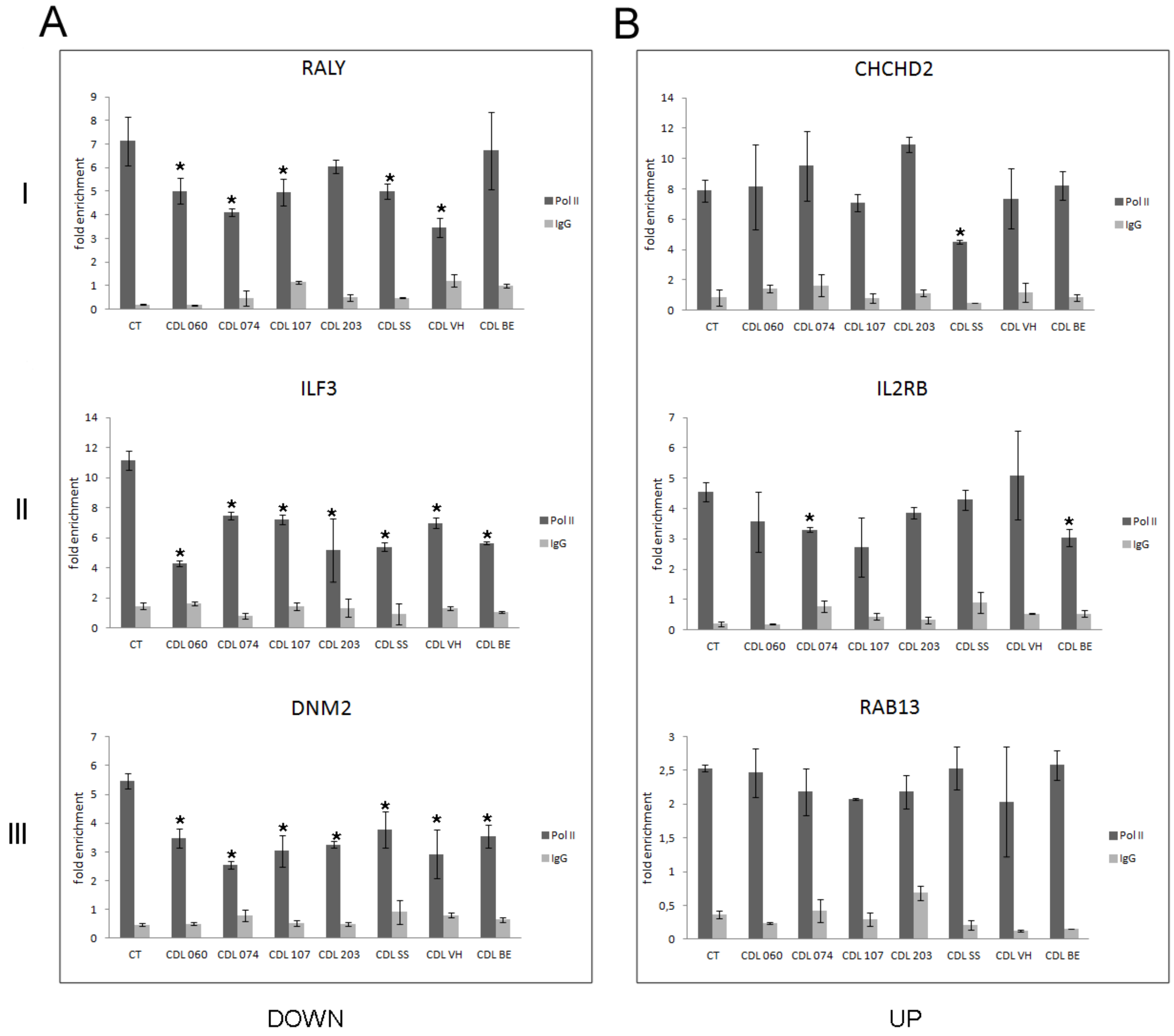

Supplementary figure 3

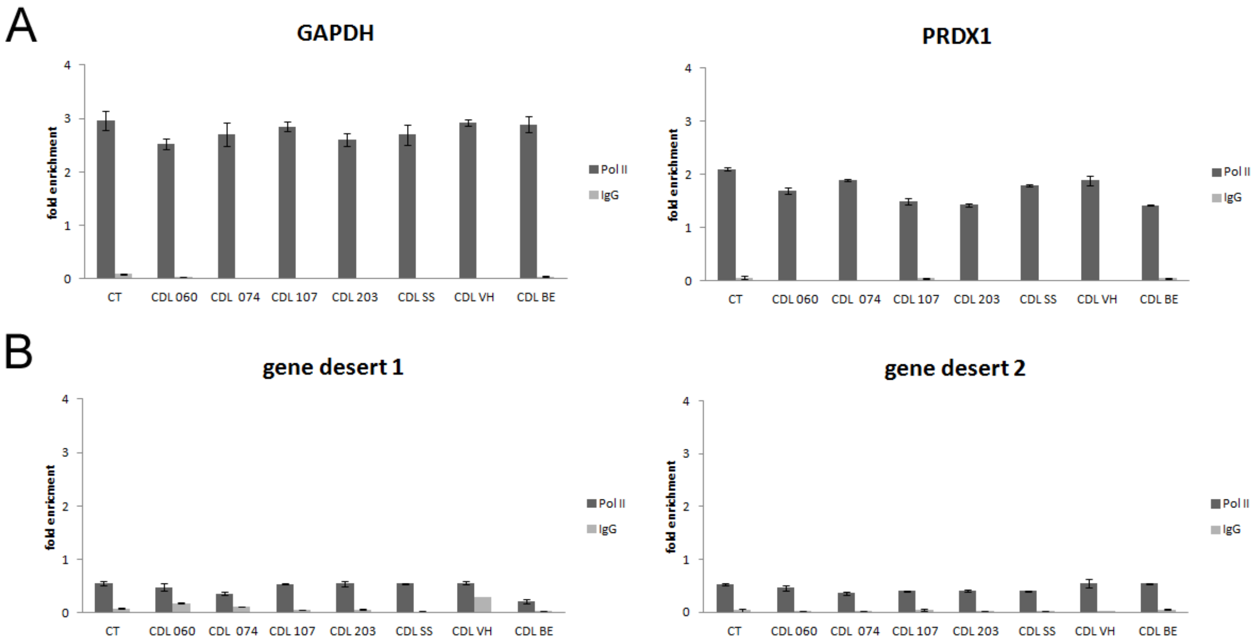

Supplementary figure 4

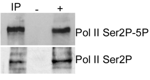

Supplementary figure 5

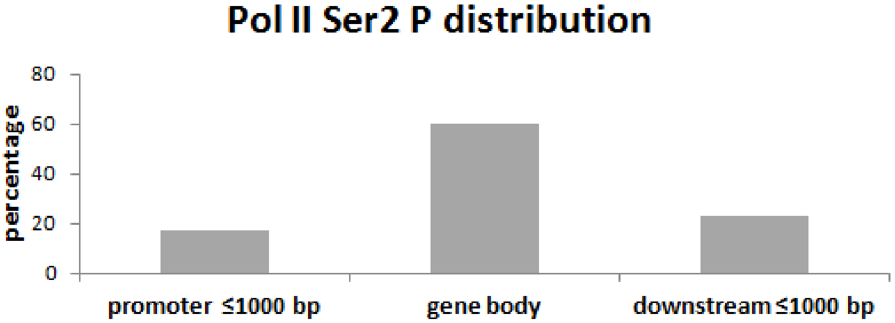

Supplementary figure 6

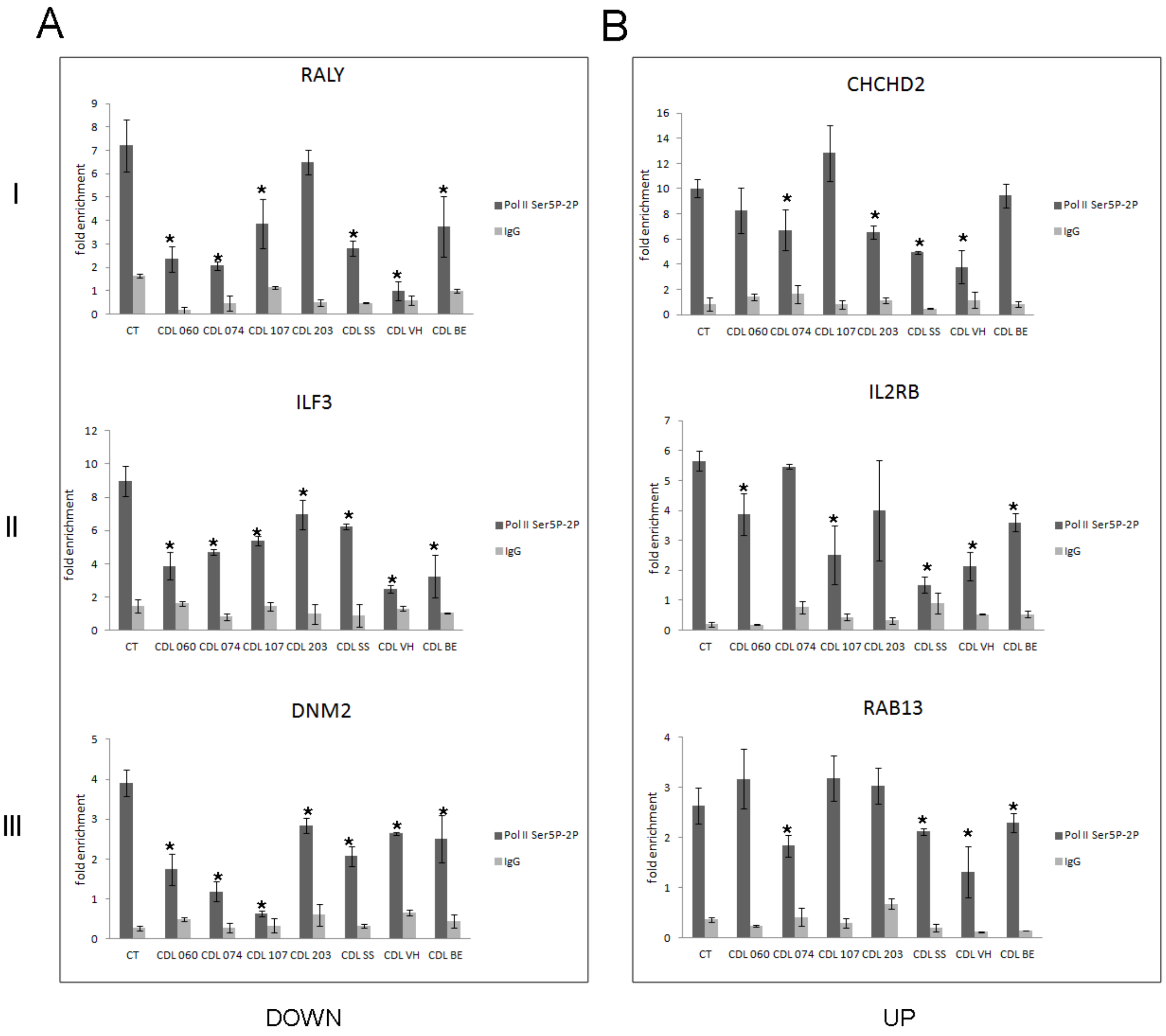

Supplementary figure 7

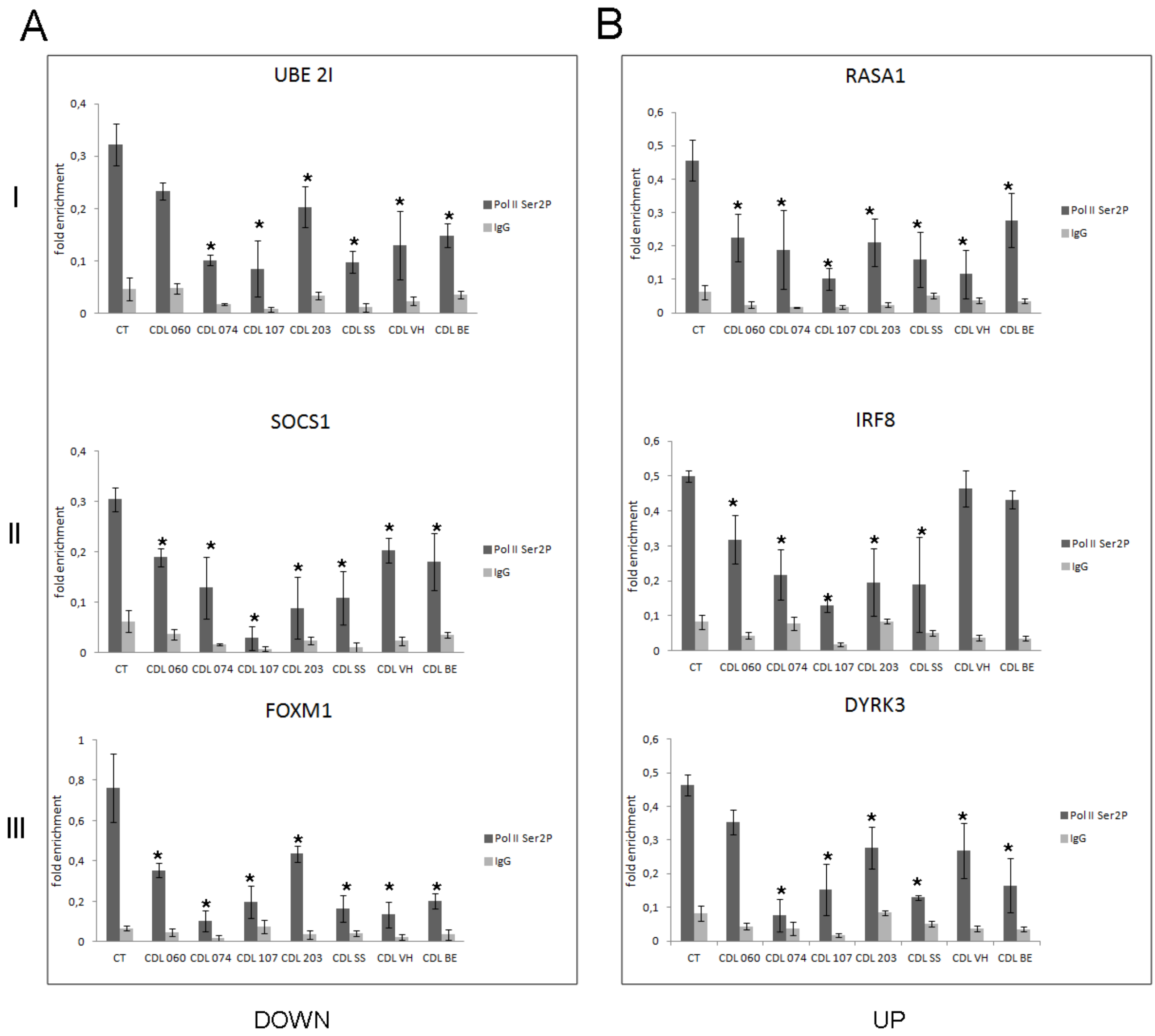

Supplementary figure 8

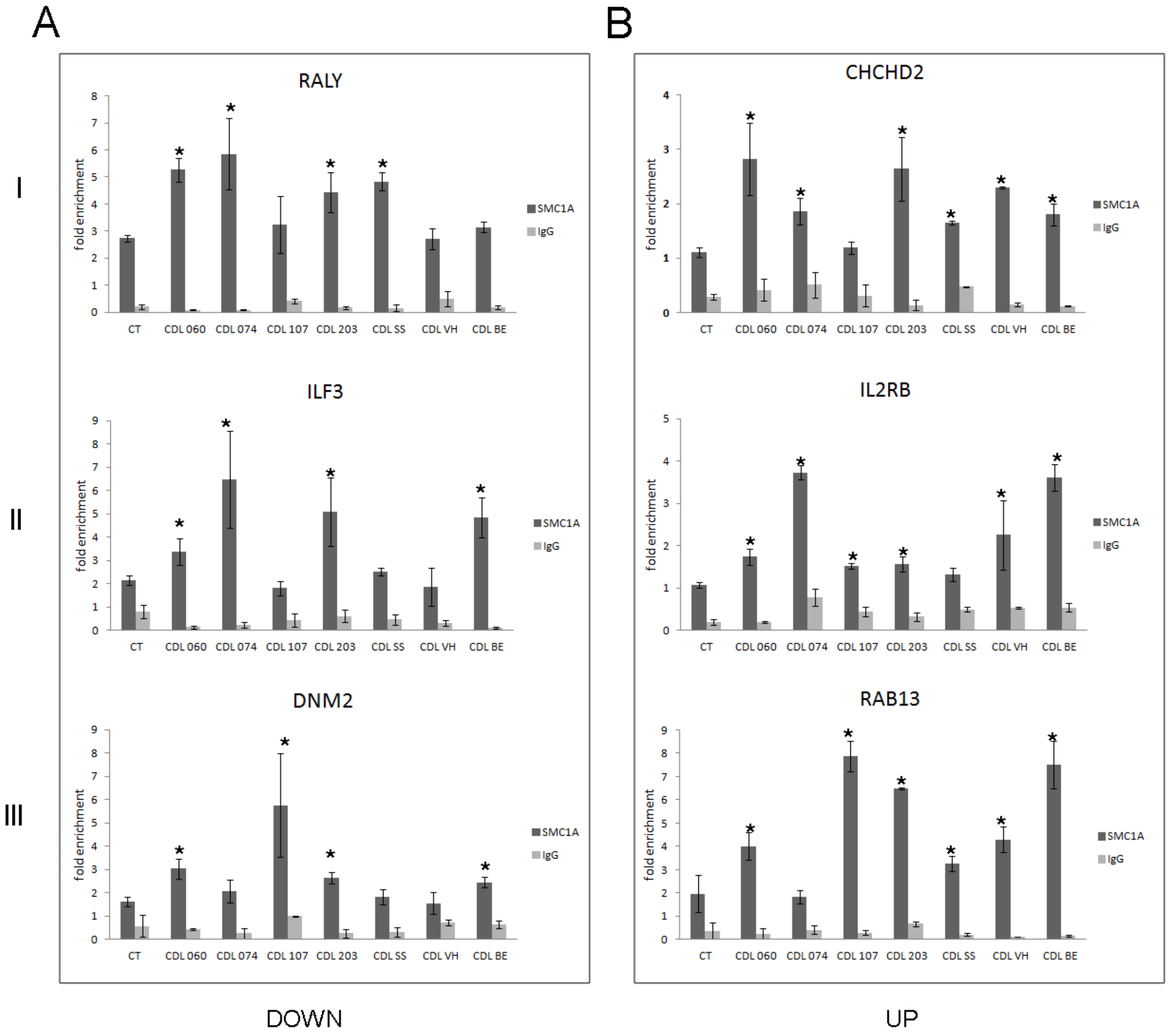

Supplement: Supplementary Information [file srep16803-s1.pdf]
